# More Than One-to-Four via 2R: Evidence of an Independent Amphioxus Expansion and Two-Gene Ancestral Vertebrate State for MyoD-Related Myogenic Regulatory Factors (MRFs)

**DOI:** 10.1093/molbev/msaa147

**Published:** 2020-06-10

**Authors:** Madeleine E Aase-Remedios, Clara Coll-Lladó, David E K Ferrier

**Affiliations:** Gatty Marine Laboratory, The Scottish Oceans Institute, School of Biology, University of St Andrews, St Andrews, Fife, United Kingdom

**Keywords:** myogenesis, chordate evolution, gene duplication

## Abstract

The evolutionary transition from invertebrates to vertebrates involved extensive gene duplication, but understanding precisely how such duplications contributed to this transition requires more detailed knowledge of specific cases of genes and gene families. Myogenic differentiation (*MyoD*) has long been recognized as a master developmental control gene and member of the *MyoD* family of bHLH transcription factors (myogenic regulatory factors [MRFs]) that drive myogenesis across the bilaterians. Phylogenetic reconstructions within this gene family are complicated by multiple instances of gene duplication and loss in several lineages. Following two rounds of whole-genome duplication (2R WGD) at the origin of the vertebrates, the ancestral function of MRFs is thought to have become partitioned among the daughter genes, so that *MyoD* and *Myf5* act early in myogenic determination, whereas *Myog* and *Myf6* are expressed later, in differentiating myoblasts. Comparing chordate MRFs, we find an independent expansion of MRFs in the invertebrate chordate amphioxus, with evidence for a parallel instance of subfunctionalization relative to that of vertebrates. Conserved synteny between chordate MRF loci supports the 2R WGD events as a major force in shaping the evolution of vertebrate MRFs. We also resolve vertebrate MRF complements and organization, finding a new type of vertebrate MRF gene in the process, which allowed us to infer an ancestral two-gene state in the vertebrates corresponding to the early- and late-acting types of MRFs. This necessitates a revision of previous conclusions about the simple one-to-four origin of vertebrate MRFs.

## Introduction

Gene duplication is widespread, but various evolutionary routes are possible for the postduplicate daughter genes. Studying which of these evolutionary routes is taken in specific cases is required to better understand the frequency, significance, and precise nature of these modes of evolution. In addition, duplication can have functional consequences and has, for example, been implicated in the evolution of vertebrate novelties (e.g., neural crest cells; Blomme et al. 2006; MacKintosh and Ferrier 2018) and the high species diversity among teleost fish (Hoegg et al. 2004).

Myogenic differentiation (*MyoD*) was first discovered as a cell-fate determinant that could convert undifferentiated cells into myoblasts (Davis et al. 1987). It has since become apparent that it is an archetypal developmental regulator of myogenesis. *MyoD* and its orthologs (e.g., fruitfly *Nautilus*, sea urchin *SUM1*, tunicate *Ci-MRF*) constitute a family of bHLH transcription factors involved in myogenesis across the bilaterians, exemplifying the ancient homology and high level of conservation of significant components of the developmental toolkit. This level of conservation allows for comparative studies of *MyoD* orthologs across the bilaterians. Throughout the evolution of bilaterians, duplication has shaped this gene family, both on the small scale via segmental duplications (e.g., sea urchin, see below) and the large, as in the two rounds of whole-genome duplication (2R WGD) at the origin of the vertebrates or 3R WGD at the origin of teleost fish.

Following WGD and rediploidization, redundant duplicate genes can be either nonfunctionalized, subfunctionalized, or neofunctionalized (reviewed by Wagner 1998; Shimeld 1999; Lynch and Conery 2000; Prince and Pickett 2002; Innan and Kondrashov 2010; MacKintosh and Ferrier 2018). Gene paralogs that arise from WGD are often referred to as ohnologs (Wolfe 2000). For housekeeping genes, one of the daughter genes tends to be nonfunctionalized (Nakatani et al. 2007), whereas developmental genes with higher regulatory complexity, especially those involved in signaling and transcription regulation like the myogenic regulatory factors (MRFs) are often retained and undergo subfunctionalization. The Duplication–Degeneration–Complementation (DDC) hypothesis explains how mutations in the regulatory regions of duplicated genes eliminates components of their expression so that both genes are expressed as a subset of the ancestral gene’s expression, and both are then required to retain the total ancestral functionality (Force et al. 1999). Complex regulatory elements provide greater opportunity for partitioning of the ancestral gene’s functions among its daughters (Blomme et al. 2006; Jimenez-Delgado et al. 2009). Neofunctionalization can arise when a novel beneficial mutation occurs in one of the daughter genes or its regulatory regions, though this seems to be far rarer than subfunctionalization (MacCarthy and Bergman 2007). Subfunctionalization consistent with the DDC hypothesis, as well as via alternative routes involving dosage sensitivity, is widely observed among genes retained in duplicate following WGDs (Lynch and Conery 2000; Zhang 2003; Kleinjan et al. 2008; Innan and Kondrashov 2010; Braasch et al. 2016). To detect subfunctionalization, preduplicate genomes are essential for comparison.

Amphioxus provide important points of comparison for the assessment of the effect of WGDs since they diverged from the rest of the chordates before the 2R WGD events at the origin of vertebrates, and can be used to infer the ancestral vertebrate preduplicate state more directly than other more distantly related invertebrates. Large-scale comparisons of gene linkage revealed the general one-to-four pattern of synteny between amphioxus and human genomes, thus supporting the role of 2R WGD in the evolution of vertebrates (Putnam et al. 2008). This has resulted in many gene families showing one to four (or fewer due to losses) paralogous relationships (Meyer and Schartl 1999), as has previously been assumed for the vertebrate MRFs. Furthermore, whole-genome-scale comparisons between the expression domains of single amphioxus genes and their vertebrate ohnologs revealed that vertebrate genes retained in duplicate had subfunctionalized and their combined distinct expression domains broadly equated to the single amphioxus genes’ expression, whereas there was no obvious difference in expression between the genes that had reverted to single copy in vertebrates (Marlétaz et al. 2018). Use of a chordate comparison allowed gene expression in more similar homologous structures to be compared between invertebrate chordates and vertebrates, and directly addressed the 2R WGD that occurred on the vertebrate stem. From this recent example, and others, there is clear evidence of subfunctionalization via DDC, but the extent to which this model applies to the MRF family is uncertain.

In vertebrates, there are four types of MRF generally recognized, which fall into two overarching functional groups: *MyoD* and *Myf5* are involved in muscle cell determination, an “early” function, whereas *Myog* and *Myf6* are principally involved in differentiation, a “late” function that is activated by the “early” MRFs (Megeney and Rudnicki 1995; reviewed by Perry and Rudnicki 2000; Moncaut et al. 2013; Buckingham and Rigby 2014; Zammit 2017), possibly as a result of postduplication subfunctionalization. Although each of the four MRFs does have a distinct role in myogenesis, there is some amount of functional redundancy between family members. For instance, knockouts of *MyoD* or *Myf5* result in fairly normal mice, whereas double-knockout of *MyoD* and *Myf5* are lethal and prevent any skeletal muscle forming in the embryo (Rudnicki et al. 1993). In the differentiation phase, *Myog* is required to activate downstream muscle genes, since *Myog* knockouts specify the muscle lineage but fail to form muscle fibers, and this cannot be rescued by *Myf6* (Buckingham and Rigby 2014). *Myf6* seems to act both “early” and “late” in mammals, but its function is redundant to *MyoD* and *Myf5* in the “early” phase, and *Myog* in the “late,” since *Myf6* knockouts have a normal phenotype, though *Myf6–MyoD* double mutants show a similar phenotype to *Myog* mutants (Rawls et al. 1995). Although *Myf6* can activate the same genes as *Myog*, *Myf6* has been implicated in downregulation of *Myog* in fully differentiated myoblasts, a distinct role from any of the other MRFs, and one which defines its function as “late” (Buckingham 2017).

In zebrafish, a 3R teleost, there are the same MRFs as in tetrapods, but their expression patterns are somewhat different. As in mice, zebrafish double mutants of *Myf5* and *MyoD* have no muscle, although either single mutant is normal, but for *Myf6* and *Myog*, both are only expressed “late,” downstream of *MyoD* and *Myf5*, and expression of either of them is only affected by *MyoD/Myf5* double mutants, not single ones (Schnapp et al. 2009). In teleosts with the typical five-gene arrangement (*MyoD1*, *MyoD2*, *Myog*, *Myf5*, and *Myf6*), the two paralogs of *MyoD* have overlapping but distinct expression profiles, consistent with subfunctionalization (Tan and Du 2002). In the 4R salmon, the three reported *MyoD* paralogs showed two distinct expression patterns (Bower and Johnston 2010), which is consistent with a partitioning of *MyoD* expression following 3R and 4R WGDs. In *Xenopus laevis*, the 3R frog, although there are two *Myog* genes with some common targets, only one activates the expression of the predominant isoform of the myosin heavy chain gene in adult muscle (Charbonnier et al. 2002). These examples show that MRFs have different, though overlapping functions following the various duplication events in different vertebrate lineages. Across all the vertebrates, we see the conservation of this general “early”–“late” distinction, where *MyoD* and *Myf5* act in determination, whereas *Myog* and *Myf6* control differentiation.

In invertebrate bilaterians, MRFs also regulate myogenesis, though most invertebrates have only one MRF gene (e.g., *Drosophila Nautilus* [*Nau*], Misquitta and Paterson 1999 and *Caenorhabditis elegans hlh-1*, Chen et al. 1994). In the echinoderms, the sea urchin has undergone an independent duplication of its MRFs, such that only *MyoD2* is required for muscle development, whereas *MyoD1* appears to have been co-opted to the skeletogenic lineage (Andrikou et al. 2013). The invertebrate chordate tunicates *Ciona* spp. have one MRF which activates expression of muscle genes in the tailbud (Izzi et al. 2013), whereas the earliest-branching chordate lineage, the cephalochordates (amphioxus or lancelets), have been reported to have varying numbers of MRFs, from one to three and with confusing nomenclature reflecting ambiguous orthology relationships among these amphioxus MRFs (supplementary information 1, Supplementary Material online; Schubert et al. 2003; Urano et al. 2003; Yuan et al. 2003; Somorjai et al. 2008; Bertrand et al. 2011; Tan et al. 2014). The amphioxus genes for which there is in situ hybridization data show expression in the myotomal component of the somites through development, and distinct patterns for the different genes (Schubert et al. 2003; Urano et al. 2003; Bertrand et al. 2011). However, because these amphioxus data are incomplete and somewhat confusing, the complement of MRFs for the chordates remains unclear.

Aside from the independent duplications in the urchin and amphioxus, invertebrates tend to have one MRF gene that is thought to be pro-orthologous to the four vertebrate MRF ohnolog clades. This might suggest a relationship marked by the vertebrate 2R WGD, where the four vertebrate MRFs are assumed to have arisen from a single invertebrate ancestral gene and duplicated twice in the 2R event, whereas invertebrate lineages typically retained a descendant of the single ancestral gene. This typical view is complicated by the fact that there are multiple amphioxus MRFs, which potentially diverged before the vertebrate expansion (Araki et al. 1996; Yuan et al. 2003). Further complicating the one-to-four assumption, the four vertebrate MRFs lie in only three distinct genomic loci; *Myf5* and *Myf6* reside in the same locus with extensively overlapping shared regulatory elements (Carvajal et al. 2008), whereas *MyoD* and *Myog* each exist as single loci on distinct chromosomes. A straightforward 2R relationship, however, would be expected to generate four paralogous loci in distinct regions of the genome, each containing one MRF.

It appears that both gene duplications and losses have obscured the expected pattern resulting from 2R WGD, though the evolution of the chordate MRFs requires more detailed phylogenetic and synteny analyses to determine the nature of their origins around, and their roles in, the invertebrate-to-vertebrate transition. Here, we show that the ancestral reconstruction for the vertebrate condition for MRFs was misled by an incomplete coverage of the vertebrate MRF gene complement and that the four-gene state of nonteleost vertebrates did not arise from a single ancestral gene undergoing two rounds of duplication. Instead, the pre-2R vertebrate ancestor had two clustered MRFs that, after the 2R events, resulted in a larger number of MRFs that then experienced secondary losses resulting in just the four ohnologs in humans, for example. Independently from this vertebrate MRF expansion, we show that amphioxus tandemly duplicated the MRF gene to produce a five-gene cluster, which has in-turn experienced subfunctionalization of the constituent genes, such that distinct amphioxus MRFs have differing expression patterns during myogenesis in the somites.

## Results

### Finding MRF Genes: New Genes and Clusters across the Bilaterians

A thorough gene search revealed several sequences previously unannotated or missing from the literature, especially in a few species with interesting duplication histories (supplementary table 1, Supplementary Material online). Although the echinoderm duplication is well-characterized for the urchin *Strongylocentrotus purpuratus*, we also found orthologs of *MyoD2* in *Lytechinus variegatus*, *Acanthaster planci*, and *Patiria miniata*, and confirmed the clustered arrangement for the first three of these species (the low-quality assembly around this locus in the *P. miniata* genome prevented confirmation of these genes’ arrangement). The clustered arrangement of these genes in two species of urchin and one sea star suggests they originated via an echinoderm-specific tandem duplication.

In the salmon genome, which we included to assess the effect of a 4R WGD, previous reports describe single *Myf5* and *Myf6* genes, located on separate chromosomes (Bower and Johnston 2010). However, we found that there are in fact two *Myf5–Myf6* clusters at two distinct chromosomal locations. Furthermore, we identified a fourth *MyoD* ortholog, which brings the total number of MRFs up to nine, when previously only six had been identified (Macqueen and Johnston 2006; Bower and Johnston 2010) (supplementary table 1, Supplementary Material online). This arrangement appears to be consistent with a duplication of the typical teleost arrangement (2× [*MyoD1*, *MyoD2*, *Myog*, *Myf5*, and *Myf6*]) followed by loss of one of the *Myog* paralogs.

Searches through representative vertebrate genomes revealed the existence of a novel MRF, which we here name *Myf7*, in the genomes of the coelacanth, spotted gar, and sterlet (supplementary information 2, Supplementary Material online; though the sterlet genome assembly available at the time was not good enough for its MRFs to be included in our subsequent analyses). This gene is located upstream of *MyoD* (10 kb in gar, 32 kb in coelacanth), with a gene related to ferritin in-between (zgc:172145; GO:0008199).

**Fig. 1.**
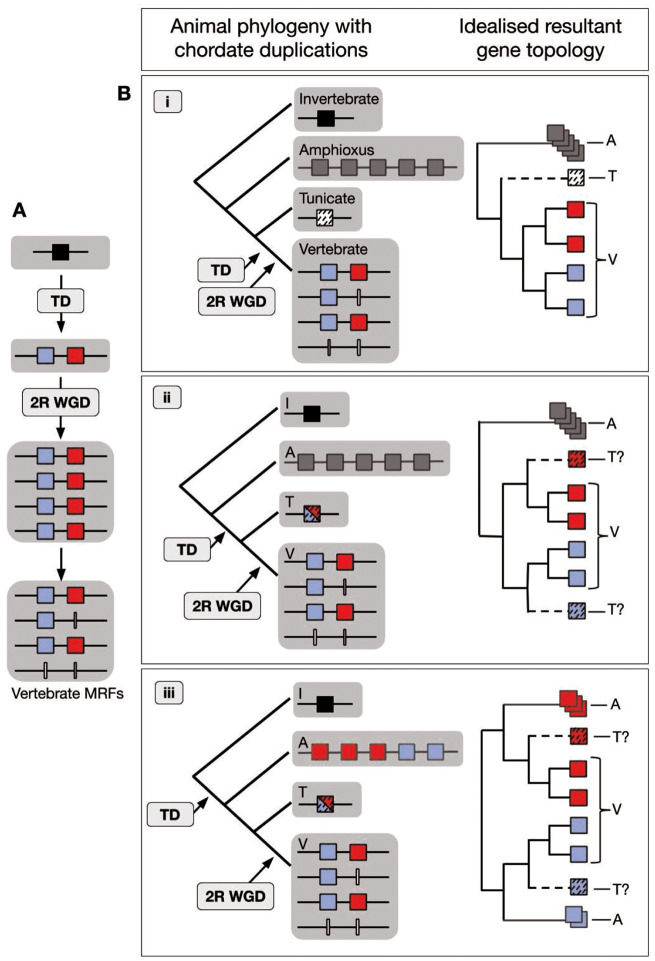
Schematic of chordate MRF evolution based on two-gene ancestral state hypothesis. (*A*) The tandem duplication (TD) predates the 2R WGD at the base of the vertebrates to generate the current vertebrate MRF gene complement. One ancestral MRF (black square) is duplicated in tandem to generate the two vertebrate MRF groups, (blue: *Myog*/*Myf6* type; red: *MyoD*/*Myf5* type), which via 2R WGD and subsequent gene losses (white lines), result in the vertebrate arrangement. (*B*) Three potential scenarios of the timing of the TD relative to the divergence of the three chordate phyla and the resultant topology of the MRF phylogeny. (i) Both amphioxus (A) and tunicates (T) diverge before the TD. (ii) The TD is after the divergence of A, so the A MRFs are an outgroup to the vertebrate (V) and T MRFs, but the TD predates the divergence of T from V so the single T MRF (red or blue, dashed) evolved from either the *Myog*/*Myf6* or *MyoD*/*Myf5* precursor, depending on which was retained. (iii) The TD predates the divergence of both the A and the T lineages. The T gene clusters either with the *Myog*/*Myf6* or *MyoD*/*Myf5* vertebrate groups depending on which precursor was retained. The five A *MRFs* similarly cluster with “either” or “both” vertebrate groups in any arrangement (shown here as three with red and two with blue), depending on which of the two post-TD genes were retained and/or duplicated.

Building on the various numbers of MRFs reported for amphioxus (see Introduction; supplementary information 1, Supplementary Material online), we found a five-gene cluster of MRFs in the amphioxus species *Branchiostoma floridae*, *B. belcheri*, and *B. lanceolatum* (supplementary table 1, Supplementary Material online). We also found incomplete sequences of all five MRFs in the genome of *Asymmetron lucayanum*, a sister lineage to the *Branchiostoma* genus, but the quality of the assembly prevented confirmation of the cluster in this species and the sequences were not complete enough to include in our phylogenies (supplementary table 1, Supplementary Material online). Nevertheless, the presence of five MRFs in the four amphioxus species surveyed suggests that the expanded cluster may be typical for lancelets as a whole. In addition, these genes share a similar exon structure to other MRFs in both invertebrates and vertebrates. Taken together, the similar exon structure and clustered arrangement of the amphioxus MRFs led us to conclude that the genes originated via tandem duplications of a prototypical chordate MRF to produce a five-gene cluster in the ancestral cephalochordate. This expansion raises multiple possibilities for the relationships among the chordate MRFs, and the timing of the various duplications relative to the divergence of the different chordate groups. The three principal possibilities are illustrated in figure 1 and discussed below. Each of these three scenarios could generate a different phylogenetic topology. Therefore, from the phylogeny, the history of the genes could potentially be determined.

### Multiple Instances of Duplication and Loss and a Two-Gene State for the Vertebrate Ancestor

**Fig. 2.**
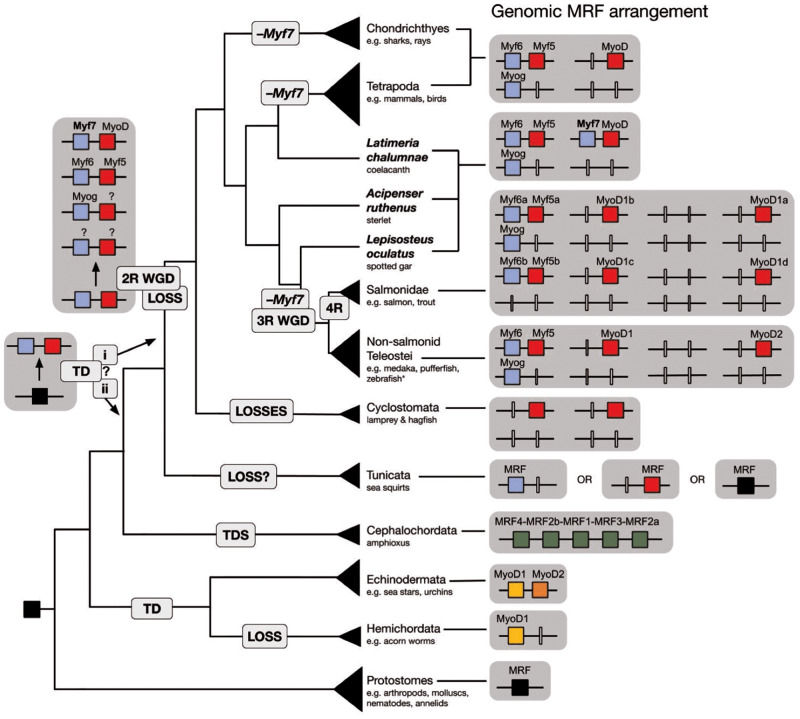
Schematic of bilaterian MRF evolution. Genomic MRF arrangement (right) for taxonomic groups (species cladogram, left) included in this study highlighting the vertebrate species that have retained *Myf7* (coelacanth, sterlet, and spotted gar, bold text), as well as duplications and losses in several lineages. Retained MRFs are represented by colored boxes, whereas genes inferred to have been lost are represented by white vertical lines. Genes clustered in the genome are joined by black lines. The uncertainty as to the timing of the vertebrate early–late tandem duplication in the vertebrate (i) or Olfactores (ii) ancestor is denoted by the “?.” *Zebrafish has lost its *MyoD2* paralog but is otherwise like the rest of nonsalmonid teleosts. TD, tandem duplication; 2R WGD, two rounds of whole-genome duplication; 3R WGD, teleost-specific third round of whole-genome duplication; 4R, salmonid-specific fourth round of whole-genome duplication; –*Myf7*, inferred loss of *Myf7*.

**Fig. 3.**
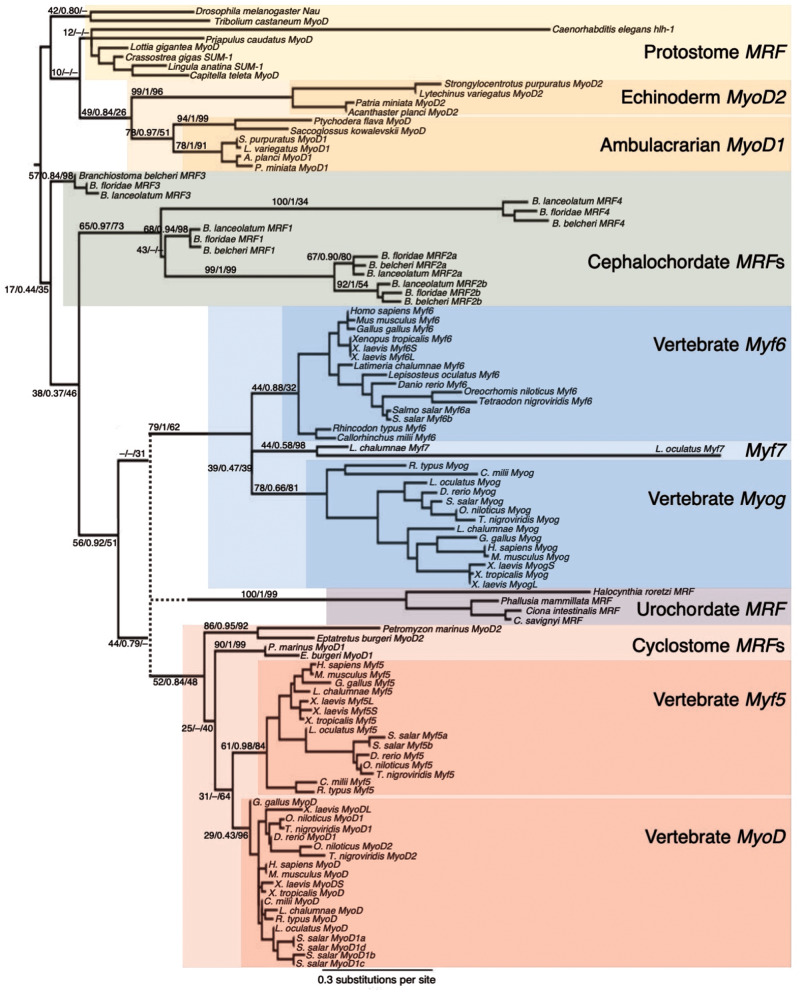
Maximum likelihood phylogeny of bilaterian MRFs. Major branches defining certain gene types, (e.g., the urochordate MRFs) are labeled with support values from three phylogenetic methods: ML (% 1,000 bootstraps)/BI (posterior probability)/NJ (% 1,000 bootstraps); values are represented by dashes when a certain topology was not present in that phylogeny. The tree is rooted on the branch separating the chordates from other bilaterians. The dotted line and corresponding support values represent the uncertainty of the placement of the urochordate sequences with support in ML and BI (44/0.79/–) and NJ (–/–/31). Colored boxes indicate gene types. The scale bar represents substitutions per site for the consensus ML tree (though not for the ambiguous urochordate branch; its node was manually drawn).

The phylogeny of MRFs from representative bilaterian taxa is consistent with several instances of duplication and subsequent gene loss (fig. 2). Our phylogenetic analyses are consistent with a single ancestral bilaterian MRF, since all the protostomes surveyed have one MRF orthologous to fruit fly *Nautilus* (fig. 3 and supplementary information 3*a–c*, Supplementary Material online). However, we infer that the two-gene state in echinoderms, where *MyoD1* and *MyoD2* are clustered in tandem is ancestral to the ambulacrarians. The hemichordates included in this study have an ortholog of *MyoD1*, but have likely lost their *MyoD2* ortholog, as evidenced by the closer relationship between the hemichordate *MyoD* genes and the echinoderm *MyoD1* than with the echinoderm *MyoD2* (fig. 3). Furthermore, although this two-gene state for the ambulacrarians might indicate a possibility for a two-gene ancestral state for the deuterostomes as a whole, we consider this less likely than a single-gene ancestral state. This is due to the topology separating the ambulacrarian genes (ambulacrarian clade support: 49/0.84/26) from the chordate genes (17/0.44/35) and the amphioxus genes from those of the Olfactores (56/0.92/51). Had the duplication occurred earlier, we would expect a topology where the duplication node separates two clades with invertebrate deuterostome and vertebrate genes distributed in both, and the single protostome MRF as a sister outgroup. Though our node support values are low, our phylogeny is more consistent with a single-gene ancestral state that independently expanded by duplications in each of the ambulacrarian, amphioxus, and Olfactores clades (fig. 2). In the echinoderms, evidence of subfunctionalization, or potentially neofunctionalization, following duplication has been documented, as of the two MRF paralogs in the urchin, *MyoD1* is co-opted to the skeletogenic lineage, whereas *MyoD2* has retained the ancestral myogenic function (Andrikou et al. 2013). The hemichordates have retained just the *MyoD1* paralog since the hypothesized ancestral ambulacrarian duplication, though it is not known if this gene has retained a myogenic function. Andrikou et al. (2013) report a third MRF in the urchin *S. purpuratus*, however, this gene showed no clear relationship to the other MRFs in our early phylogenetic analyses, so this gene was excluded from the final trees.

**Fig. 4.**
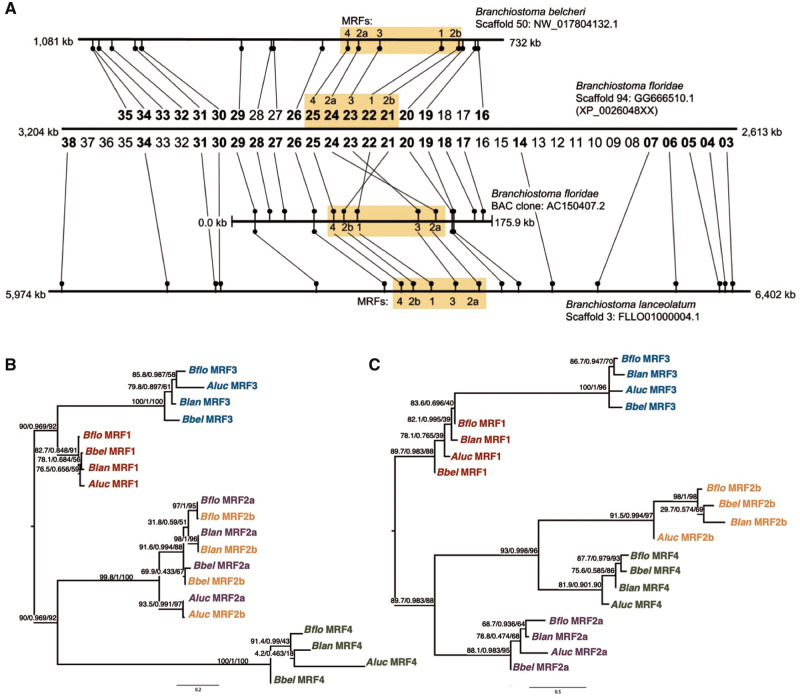
Physical and phylogenetic relationships of the five amphioxus MRFs. (*A*) Genomic arrangement of five-gene MRF cluster in three *Branchiostoma* spp. genomes. The *MRF* scaffolds from the *B. belcheri*, *B. floridae*, and *B. lanceolatum* genome with orientation of the MRF clusters compared with the orientation of flanking genes. Genes are from predicted *B. floridae* models with corresponding protein accession numbers in the format XP_00206048–.1 (e.g., *MRF4* is XP_0020604825.1). Bold numbers are genes with orthologs confirmed on the other scaffolds using TBlastN. (*B*) Phylogeny of MRF exon one. IQ-TREE ML phylogenies with ultrafast bootstrap support, SH-aLRT support, and approximate Bayes posterior probability values. (*C*) Phylogeny of MRF exons two plus three. As in (*B*), separate exon alignments were made from sections of the full sequence of all five amphioxus MRFs (supplementary information 4*a*, Supplementary Material online).

Within the chordates, the phylogeny supports the independence of the amphioxus expansion as first proposed by Araki et al. (1996), since all five amphioxus MRFs branch within the chordate clade, albeit with low support (chordate clade support: 17/0.44/35), but outside the Olfactores clade (Olfactores [vertebrates and urochordates] clade support: 56/0.92/51) (fig. 3). Thus, we infer that this five-gene amphioxus cluster diverged from a one-gene ancestral state, that is, before the duplication that gave rise to the two vertebrate MRF types (fig. 2). Two of the amphioxus MRFs share a first exon sequence (supplementary information 4, Supplementary Material online) and this exon is more similar within species than within genes (fig. 4*B*). Although a phylogeny of the second and third exons results in a topology where each gene forms a distinct clade (fig. 4*C*), the phylogeny of the first exon sequence has each species’ *MRF2a* and *MRF2b* clustering together within a larger overall *MRF2a*/*MRF2b* clade (fig. 4*B*).

The placement of the urochordate MRF gene could not be reliably determined by the three phylogenetic methods, probably because of the lack of conservation outside of the bHLH domain and its long, divergent sequence, especially that of the N-terminal domain (fig. 3, Ratcliffe et al. 2019). Even editing the alignment to reduce the inclusion of divergent urochordate sequence did not significantly improve resolution. Nevertheless, all three phylogenetic methods placed the urochordate sequences as sister to one of the vertebrate clades, and there is moderately high support for the branch that groups the Olfactores sequences together (support: 56/0.92/51) (fig. 3). From this, we tentatively conclude that the urochordate *MRF* represents an ortholog of one of the ancestral vertebrates’ two pre-2R genes, though to which one it is more similar could not be determined (supplementary information 3*d* and *e*, Supplementary Material online; clustered with *MyoD*/*Myf5*: BI: 0.79 and ML [with cyclostomes]: 44; with *Myog*/*Myf6*: ML [without cyclostomes]: 52 and NJ: 31), and this topology, albeit uncertain, supports the scenario where the urochordate sequence diverged from the ancestral two-gene state. However, the low node support values and differing topologies between the phylogenetic methods mean that we cannot confidently exclude the possibility that the urochordate *MRF* is instead directly descended from the ancestral single-copy state of the chordate ancestor.

In the vertebrates, we infer an ancestral two-gene state, originating from a tandem duplication of the ancestral chordate *MRF*. It was previously thought that the four vertebrate MRFs arose from a single gene, duplicated twice via 2R WGD, but this hypothesis would require a highly specific translocation to produce the *Myf5–Myf6* cluster. This now seems unlikely because of our discovery of *Myf7* (fig. 2). This new MRF is adjacent to *MyoD* in a two-gene cluster in the coelacanth, spotted gar, and sterlet, which share an ancestor at the origin of all bony fish (Osteichthyes) (fig. 2). Therefore, in three independent cases: at the base of the tetrapods, within the ray-finned fish (between the origins of the Neopterygii and Teleostei), and within the cartilaginous fish (Chondrichthyes), we infer losses of the ortholog of the *Myf7* gene (fig. 2). *Myf7* is located in the *Myog*/*Myf6* clade in the phylogeny, which means the *MyoD–Myf7* cluster with one gene of each type mirrors the *Myf5–Myf6* cluster throughout the vertebrates. Based on the topology of the phylogeny (fig. 3), and the grouping of the urochordate MRFs within the vertebrate clade(s) (figs. 1*B*.ii and 2), we now tentatively infer that a tandem duplication at the base of the Olfactores generated two types of MRF, “early” like *MyoD* and *Myf5*, and “late” like *Myog* and *Myf6*. After the divergence of the urochordates, 2R WGD then duplicated this cluster twice, followed by several losses to generate the vertebrate MRF gene complement: the *MyoD–Myf7* cluster, the *Myf5–Myf6* cluster, and *Myog*. The fact that each of these clusters has one gene of each type of MRF, “early” and “late,” makes our “two-gene state followed by 2R” hypothesis the most straightforward explanation for the origin of the vertebrate MRF condition and suggests that the role of gene loss in the evolution of this gene family had previously been underestimated.

In addition to the urochordate *MRF* proving difficult to place in the phylogenetic tree, the cyclostome sequences also have a poorly resolved location in the phylogeny. Nevertheless, we can reliably infer that the two genes in the lamprey and hagfish genomes, named *MyoD1* and *MyoD2* (not corresponding to teleost or echinoderm paralogs), belong to the vertebrate *MyoD/Myf5* clade (fig. 3). However, whether these genes represent divergent orthologs of *MyoD* and *Myf5* themselves, or orthologs of the *MyoD/Myf5*-type genes lost from other vertebrates is not clear.

We also observe the effect of lineage-specific duplications on the MRF family, particularly following the 3R teleost-specific and the 4R salmonid-specific WGDs (fig. 2). Following the 3R teleost-specific WGD, the teleosts retained only one of the MRFs in duplicate (paralogs *MyoD1* and *MyoD2*), whereas the other genes returned to the single-copy state. This is an instance of secondary gene loss (nonfunctionalization), which is even more pronounced in the zebrafish that have lost the ortholog of *MyoD2* as well. In the salmon, we find nine MRFs: two orthologs each of *Myf5* and *Myf6*, one of *Myog*, and four of *MyoD*. This appears to be a direct duplication of the typical teleost arrangement consistent with the 4R salmon-specific WGD, followed by the loss of a second *Myog* ortholog. However, interestingly the four salmon *MyoD* orthologs do not separate along the *MyoD1/MyoD2* divide in the phylogeny (fig. 3), and previously, the three known *MyoD* genes were named *MyoD1a*, *MyoD1b*, and *MyoD1c* and were hypothesized to have originated independently of the 4R WGD. This intriguing salmon pattern is clarified by an examination of gene synteny and conservation across the vertebrate MRF loci (see below).

### Conserved Synteny and Ghost Loci

Across the vertebrate phylogeny, we see evidence of gene losses, including the inferred loss of all MRFs from the fourth 2R WGD-generated locus, leaving a ghost MRF locus that is widespread across vertebrates. Investigation of the gene neighborhoods of the three retained MRF loci reveals a pattern of 4-fold synteny in the vertebrates, linking the three MRF-bearing chromosomes and another chromosome in certain species, an MRF ghost locus (supplementary information 5*a–d*, Supplementary Material online). In the human genome, the MRFs are located on chromosomes 1 (*Myog*), 11 (*MyoD*), and 12 (*Myf5* and *Myf6*), whereas paralogs of MRF gene neighbors in gene families with four or fewer genes are also found on chromosome 19 (supplementary table 2 and information 5*a*–*d*, Supplementary Material online). Other studies highlighting 4-fold paralogy of the human genome also group chromosomes 1, 11, 12, and 19, (Dehal and Boore 2005; Putnam et al. 2008; Srivastava et al. 2008; Craxton 2010; Bertrand and Escriva 2011). From this network of paralogous genes linking these human chromosomes, we found the orthologs of these genes in other species, which revealed a pattern of conserved 4-fold synteny across the subset of vertebrate genomes analyzed (though the ghost locus was not intact in the chicken genome and it is uncertain if there is one in the lamprey given the state of the assembly) (supplementary table 2*a–c*, Supplementary Material online). There is also evidence for instances of rearrangement, including chromosomal fusion and fission in the mouse–human comparison, consistent with high levels of rearrangement in the mouse genome (Bourque et al. 2004).

Despite several instances of rearrangement, we were still able to find evidence of four conserved neighborhoods linked by ohnologous gene families, supporting a 2R history of the vertebrate MRF loci. Furthermore, 8-fold synteny was revealed in the zebrafish genome, as a result of the 3R WGD, which builds on Macqueen and Johnston’s (2008) identification of the *MyoD2* ghost locus (supplementary table 2*a–c*, Supplementary Material online). In the case of the salmon *MyoD* gene phylogeny mentioned above, we find evidence that suggests a history consistent with direct duplication via 4R: the four salmon *MyoD* loci reside on two pairs of homologous chromosomes (Lien et al. 2016), and two of the loci appear to have homology to the *MyoD1* locus in 3R teleosts (*MyoD1b* and *MyoD1c*), whereas the other two match more closely to the *MyoD2* locus (*MyoD1a* and *MyoD1d)*, including the ghost *MyoD2* locus in zebrafish identified by Macqueen and Johnston (2008) (supplementary table 2*d*, Supplementary Material online). This then indicates that the four *MyoD* genes in salmon secondarily evolved to converge on a distinct *MyoD* sequence without particular similarity to the teleost *MyoD1* versus *MyoD2* genes, as evidenced by the phylogeny.

Conserved synteny can also be traced back to the base of the chordates, where we find significant conservation of orthologs shared between the amphioxus MRF locus and two of the four 2R ohnologous chromosomes in human. There was a strong signal between the *B. lanceolatum* MRF neighborhood and human chromosomes 11 and 12 (binomial test, 11: *P *<* *2 × 10^−3^, 12: *P *<* *1 × 10^−2^; Barnard’s exact test, 11: *P *<* *1 × 10^−10^, 12: *P *<* *2 × 10^−8^; supplementary information 6*c*–*d*, Supplementary Material online), whereas there was no significant association observed with chromosome 1, and only a marginally significant association with 19 in one of the tests (Barnard’s exact test, *P *<* *0.05). Nevertheless, our findings are consistent with previous comparisons of human and amphioxus orthology. Putnam et al. (2008) found significant conserved synteny between several *B. floridae* scaffolds (which we found are homologous to the larger MRF scaffold in *B. lanceolatum*) and the ancestral linkage group #14, which corresponds to sections of human chromosomes 11 and 19. The homology between the reconstructed ancestral linkage groups and the human genome was updated by Srivastava et al. (2008), who link the same regions of chromosomes 11 and 19 found by Putnam et al. (2008), as well as regions of chromosomes 1 and 12 in an ancestral linkage group renamed as #2 (supplementary information 6*e*, Supplementary Material online). Many of the human orthologs of *B. lanceolatum* MRF neighbors that we assigned are located in regions of the human chromosomes corresponding to this ancestral linkage group, suggesting that this linkage group contains the ancestral chordate MRF locus. This further supports the 2R history of the vertebrate MRF arrangement and illustrates the one-to-four relationship of the MRF loci in amphioxus and human (supplementary information 6, Supplementary Material online).

### Subfunctionalization of Amphioxus MRFs

**Fig. 5.**
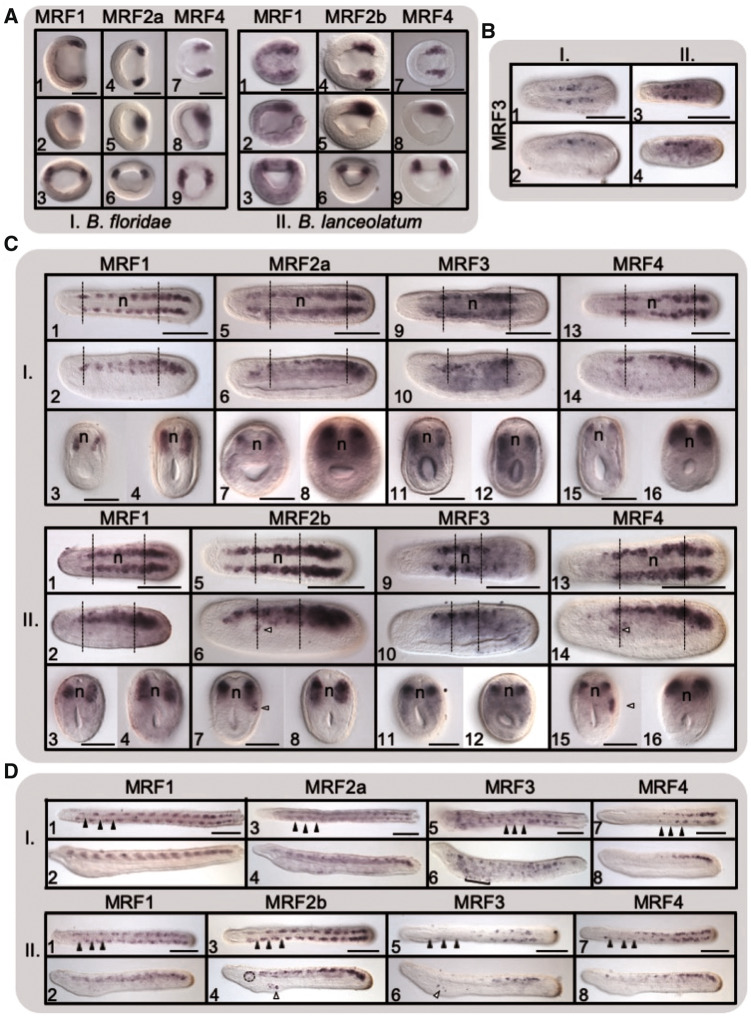
Expression time-course of amphioxus MRFs. WMISH of (*A*) late gastrula, (*B*) early neurula (up to 8 somites), (*C*) mid-late neurula (10–12 somites), and (*D*) early larva (12–15 somites) stages of (I) *Branchiostoma floridae* and (II) *B. lanceolatum* embryos. For both species at all stages, the top rows show dorsal views and the second rows show lateral views (anterior is to the left, scale bars represent 100 µm). For (*A*.I.) and (*A*.II.), the bottom row is the posterior view from the blastopore (dorsal to top). For (*C*.I.) and (*C*.II.), the bottom rows are cross-sections through the dotted lines in the anterior (left) and posterior (right) somites (scale bars represent 50 µm). Full arrowheads in (*D*.I.) and (*D*.II.) represent somite boundaries. Nonmyotomal expression is denoted in the neurulae with the open arrows (*C*.II.6 and II.7: expression of *MRF2b* in the somatic [parietal] layer left of the third somite and *C*.II.14 and II.15: expression of *MRF4* in the somatic [parietal] layer of mesoderm of the third anterior left somite) and in the larvae with the square bracket (*D*.I.6: pharyngeal expression of *MRF3*), open arrowheads (*D*.II.4: expression of *MRF2b* in pharyngeal gill slits and *D*.II.6: expression of MRF3 in the mouth rudiment) and the circle (*D*.II.4: expression of *MRF2b* in the wall of the preoral pit).

The distinct roles of the two daughter types of MRF in vertebrates, as well as the functional distinction between the two *MyoD* genes in the urchin, have been well documented (see Introduction). Similarly, early studies characterized distinct roles for the few then-identified MRFs in amphioxus (see Introduction). Now, with the complete set of amphioxus genes, we generally see that the amphioxus MRFs are expressed in the mesodermal cells surrounding the blastopore in the late gastrula stage, and later in the somites continuing through the early larval stage (fig. 5 and details supplementary information 7, Supplementary Material online), consistent with previous descriptions of expression (Schubert et al. 2003; Urano et al. 2003), though there are clear differences in expression of these five genes indicating that they have undergone subfunctionalization. There are also several differences in expression between the two amphioxus species we studied, though some may be due to different developmental rates.

The amphioxus MRFs are expressed in distinct regions of the somites at different stages of development. In *B. floridae*, *MRF1*, *MRF2a*, and *MRF4* are first detected in the gastrula in two regions at the lip of the blastopore that are consistent with locations for presomitic mesoderm (fig. 5*A*;*MRF2b* expression shown in supplementary information 7, Supplementary Material online). *MRF3*, however, is first detected in the early neurula, expressed only in the central somites (fig. 5*B*.I). In the late neurula stage, *MRF1* and *MRF2a* are expressed in all the somites, with a stronger expression signal extending further ventrally in the more posterior somites (fig. 5*C*.I). At this stage, *MRF3* expression pattern expands from just the central somites to all the somites except the most anterior pair and those in the tailbud (fig. 5*C*.I). In the late neurula, *MRF4* expression is similar to that of *MRF1* and *MRF2a*, but it is not detected in the central somites, leaving a gap between the anterior and posterior regions of expression (fig. 5*C*.I.13 and I.14). Within the somites, *MRF1*, *MRF2a*, and *MRF4* expression extends ventrally in more posterior somites but is restricted dorsally in anterior somites (fig. 5*C*.I), whereas *MRF3* expression extends ventrally in all the somites in which it is expressed (fig. 5*C*.I.12). The expression patterns of *MRF1*, *MRF2a*, and *MRF3* continue as before along the anterior–posterior array of somites into the late larval stage (fig. 5*D*.I), but *MRF4* is detected solely in the posterior somites (fig. 5*D*.I.7 and I.8), and by the late larval stage, only *MRF1* is still detected (supplementary information 7, Supplementary Material online).

The *MRF* expression pattern is generally similar in *B. lanceolatum*, however it is *MRF2b* that is detected throughout most of the anterior–posterior extent of the somites just as *MRF2a* is in *B. floridae* (*B. lanceolatum MRF2a* expression in supplementary information 7, Supplementary Material online). *MRF1*, *MRF2b*, and *MRF4* follow the pattern of *B. floridae MRF1*, *MRF2a*, and *MRF4*, respectively (fig. 5*A–D*.II). *MRF4* remains expressed in the central somites in the late neurula stage, that is, there is no gap (fig. 5*C*.II.13 and II.14), but in the larva, we see the same restriction of *MRF4* to the more posterior somites as in *B. floridae* (fig. 5*D*.II.7 and II.8). Within the more posterior somites, expression is expanded further ventrally than in the anterior somites for *MRF1*, *MRF2b*, and *MRF4*, as in *B. floridae* (fig. 5*C*.II). *MRF3* is again first detected in the early neurula and is only seen in the central somites (fig. 5*B*.II), but it remains in a more restricted anterior–posterior array of somites than the more extensive expression seen in *B. floridae* (fig. 5*C–D*.II). Overall, we observe some differences in the expression of the MRFs, but for the most part there are similar expression patterns for each gene in the two species, therefore, we see a similar pattern of subfunctionalization of the five amphioxus *MRFs*.

Some of the *MRFs* possibly also exhibited nonmyotomal expression in the presumptive precursors to the orobranchial musculature. This was not detected in all the larvae and differs between the species; therefore, these patterns are reported with caution. *MRF3* was detected in the prospective gill slits of one-third of *B. floridae* larvae (fig. 5*D*.I.6), and in the mouth rudiment of half of the *B. lanceolatum* larvae (fig. 5*D*.II.6). *Branchiostoma lanceolatum MRF2b* was detected in the first primary gill slit (fig. 5*D*.II.4) in all larvae.

## Discussion

Careful manual curation of available genomic sequence data combined with newly available whole-genome sequences has enabled us to produce a picture of MRF evolution at the invertebrate–vertebrate transition that is significantly different from the previous consensus. Automatic or pipeline annotations frequently make errors in predicting gene orthologies, which can drastically mislead evolutionary reconstructions and biological inferences. This is particularly evident in the case of *Myf7* (supplementary information 2, Supplementary Material online), a vertebrate MRF described here for the first time. The way these *Myf7* genes were previously annotated gave the impression that the *Myf5* gene had translocated from adjacent to *Myf6*, to be adjacent to *MyoD*. It is now clear that *Myf7* represents a novel MRF, linked to *MyoD* in the genomes of the coelacanth, sterlet, and gar. Based on our phylogeny, *Myf7* is more similar to the *Myog* and *Myf6* clade than the *MyoD* and *Myf5* clade, so that the *MyoD*–*Myf7* cluster mirrors the *Myf5*–*Myf6* cluster conserved across the vertebrates. The presence of two parallel clusters suggests the origin of the vertebrate MRFs from a single two-gene cluster that predates the 2R WGDs at the base of the vertebrates.

It has long been recognized that the vertebrate MRFs fall into two distinct functional types, which also relates to their paired grouping in phylogenetic trees. *MyoD* and *Myf5* are most closely related to each other in phylogenies and these are the MRFs expressed first during myogenesis, during the determination phase. *Myf6* and *Myog* are then expressed in the next phase of myogenesis controlling the differentiation into muscle fiber cells, and *Myf6* and *Myog* are in-turn most closely related to each other. The grouping of *Myf7* with the *Myog*/*Myf6* group suggests that *Myf7* might also have a “late” rather than “early” role in myogenesis, though its function has yet to be addressed. Our finding that the pre-2R vertebrate ancestor had two rather than one MRF correlates precisely with this expression and functional data and we hypothesize that the vertebrate ancestor had a two-gene cluster with a *MyoD/Myf5* precursor that controlled myogenic determination and a *Myf6/Myog* precursor that directed myogenic differentiation. This suggests that there was a partitioning of the ancestral MRFs role following the duplication that generated the two MRF types, leading to the early- and late expression of the subfunctionalized daughter genes.

The duplication that gave rise to the two functional types of vertebrate MRF, “early” and “late,” predates the 2R WGDs. These large duplication events have been implicated in many vertebrate advancements that also occurred at this time (Holland et al. 1994; Shimeld and Holland 2000; Blomme et al. 2006; MacKintosh and Ferrier 2018). Before that, however, is the duplication followed by subfunctionalization that resulted in the two functional types of MRF. This ancestral vertebrate MRF duplication may be linked to increasing complexity not only in the myogenic regulatory network but also in possible vertebrate-specific muscle innovations like myoblast fusion to produce multinucleate muscle fibers compared with the ancestral chordate, which may have had mononucleate muscle fibers or lamellae as in amphioxus (Peachey 1961; Flood 1967). Furthermore, the four vertebrate MRFs act in distinct but overlapping patterns, inferred to be specific subsets of the expression of an ancestral gene, but they have also subfunctionalized in sequence. The helix-III domain confers target specificity and differs between the two gene types, “early” and “late,” and is linked to the differentiation of their roles in regulating myogenesis (Bergstrom and Tapscott 2001). The pre-2R tandem duplication may have had important functional consequences for myogenesis, as has been also inferred for the 2R WGDs and development as a whole.

Among the vertebrates, further WGDs have shaped the MRFs. Although the instances of non- and subfunctionalization of the MRFs following the teleost 3R are well characterized (e.g., the subfunctionalization of *MyoD1* and *MyoD2*; the losses of paralogs of *Myog*, *Myf5*, and *Myf6* and *MyoD2* in zebrafish), only using the new assembly of the salmon genome from 2016 (Lien et al. 2016) could we identify a complement of nine salmon MRFs, thus extending beyond the six previously described genes. The synteny analyses of Lien et al. (2016) revealed the distribution of paralogous segments of the salmon chromosomes that originated in the 4R salmon-specific-WGD, which in-turn informed our synteny analysis, especially when characterizing the four *MyoD* loci. This new extended complement and classification of the salmon MRFs has implications for the expression studies of these genes, as the high-sequence similarity between *MyoD1a* and the novel *MyoD1d* suggest that previous assays on *MyoD1a* would also detect *MyoD1d* and the biphasic expression observed by Bower and Johnston (2010) could reflect two different or overlapping expression domains of *MyoD1a* and *MyoD1d*, respectively.

The new assembly of the *B. lanceolatum* genome (Marlétaz et al. 2018) was also instrumental in our analyses, as it allowed for a more thorough comparison of a larger contiguous amphioxus locus than were available from the *B. floridae* or *B*. *belcheri* genome assemblies. This MRF scaffold also had many more genes annotated to it than the previous assembly, which allowed us to use it as a starting point for the reciprocal BlastP searches to detect synteny with vertebrate loci. In the comparison between the human and amphioxus loci, we only found a strong signal of orthology for human chromosomes 11 and 12 to the amphioxus MRF locus, but we did not find strong signal associating the amphioxus MRF neighborhood to human chromosomes 1 or 19. These chromosomes (11 and 12) also have a larger component of ancestral linkage group #2 (Srivastava et al. 2008) (supplementary information 6, Supplementary Material online), which is only homologous to small regions on human 19 and 1. Although human chromosome 19 has the ghost locus (supplementary table 2 and information 5, Supplementary Material online), and therefore, we know has lost an MRF and potentially several neighbors, it still retains more orthologs to the *B. lanceolatum* MRF neighborhood than expected by chance, as detected by one of our tests (supplementary information 6, Supplementary Material online). Chromosome 1, despite having the third largest number of 1:1 orthologs with the *B. lanceolatum* MRF scaffold, did not show statistical significance, perhaps because it is the largest, most gene-rich chromosome, and/or has undergone more rearrangements, as this chromosome has homology to more linkage groups than any of the others considered, all of which may have diluted the signal of the 2R homology.

Although there is only one MRF locus in amphioxus, compared with three (and a ghost) in human and other tetrapods, amphioxus underwent an independent expansion of their MRFs at this locus. Because the five-gene cluster was found in three *Branchiostoma* species, and the presence of the five genes was confirmed in *Asymmetron lucayanum*, we can infer that this expansion occurred in their ancestor and, due to the clustered arrangement, the expansion likely arose via a series of tandem duplications. Interestingly, the protein sequence of the first exon of *MRF2a* and *MRF2b* is identical, hence their naming, whereas the sequences for the second and third exons are not more closely related than to other genes, rather *MRF4* is grouped with *MRF2b*, before *MRF2a*. The phylogeny of the first exon amino acid sequences groups the *MRF2a* and *2b* proteins together with a topology that matches the species tree, whereas the phylogeny for the second and third exons separates the two genes into distinct clades (fig. 4*B and C*). Perhaps this is a result of gene conversion between the first exons of the two *MRF2* genes, or it reflects a more recent duplication and more constraint on the sequence of the first exon. However, both of these hypotheses are somewhat compromised by the locations of the genes in the cluster, with *MRF2a* and *2b* being separated by the *MRF3* and *1* genes.

There is some discrepancy between genome assemblies with regards to the arrangement of the amphioxus MRFs (fig. 4*A*). In the *B. floridae* and *B. belcheri* genome assemblies, the order is *MRF4–MRF2a–MRF3–MRF1–MRF2b*, whereas in the *B. floridae* BAC clone and the newer *B. lanceolatum* assembly, the order is *MRF4–MRF2b–MRF1–MRF3–MRF2a*, though the neighboring genes in the *B. floridae* genome are also present around the MRF cluster in the same arrangement in *B. lanceolatum*, such that it is only four of the five MRFs that are differently arranged (fig. 4*A*). Long-range PCR experiments have confirmed the arrangement of the BAC clone (Coll-Lladó C, unpublished data), but have not resolved the gene arrangement in either *B. floridae* or *B. lanceolatum* genomic DNA. Whether this discrepancy is due to an assembly error in one or other amphioxus genome, perhaps conflated by the near-identical nucleotide sequences for the first exon of *MRF2a* and *MRF2b*, which sit at either side of the four genes that are “flipped” (fig. 4*A*), remains to be resolved.

The independence of the amphioxus MRF expansion was first hypothesized by Araki et al. (1996), based on their phylogeny of vertebrate MRFs and the two amphioxus MRF genes known at that time in *B. floridae*. They also were the first to present the question as to whether there was an ancestral one- or two-gene state. Critically, they did not include the occurrence of the 2R WGD on the vertebrate stem in their analysis and inferred instead that the vertebrate arrangement arose via a tandem gene duplication to generate a two-gene cluster (either before or after the divergence of amphioxus from the vertebrates), which then itself duplicated in vertebrate evolution, followed by the separation of the *MyoD* and *Myog* genes onto distinct chromosomes. This was echoed by Yuan et al. (2003) and Schubert et al. (2003). We have updated their possible scenarios to include the five-gene cluster in amphioxus, the one-gene state of the urochordates, and the impact of 2R on the vertebrate arrangement. This gave three possible scenarios (fig. 1*B*). The combination of our phylogeny and synteny analysis allowed us to confirm the independence of the amphioxus expansion, and that the vertebrate arrangement arose from a two-gene state.

The many instances of subfunctionalization, or reductions of the expression of retained semiohnologs, observed between amphioxus and the vertebrates (Marlétaz et al. 2018) could help explain the increased complexity of vertebrates, specifically via their gene regulatory landscapes, and the MRFs contribute to this model. However, the MRFs are a particular case where there has been an independent expansion and subfunctionalization in the preduplicate lineage as well. It has even been suggested that complexity evolved convergently among the chordates since the single *Ciona MRF* gene has two splice variants that are differentially expressed in an early-versus-late pattern which mirrors that of the vertebrates (Meedel et al. 1997). It is unclear whether the *Ciona MRF* splice variants are also expressed in different parts of the embryo, and whether it does indeed have an autoregulatory role in the switch between the short and long variants (Meedel et al. 2002). Still, coupled with the independent amphioxus expansion, this shows that myogenesis in the preduplicate invertebrate chordates is more complex than previously thought.

In the invertebrate chordate amphioxus, we find overlapping but distinct expression patterns of the five MRFs, as well as a few differences in expression in the two species assessed. The pattern of *MRF1* expression was the same for both species as has been previously reported for these species (Somorjai et al. 2008). In addition, *MRF3* is first expressed at a later stage relative to the other *MRFs* in both species, and *MRF4* is restricted to the posterior of the larvae of both species. Between the species, *MRF2a* and *MRF2b* likely show differences in their expression patterns, however, this data must be interpreted with caution due to the similarity of the two sequences. Amid similar patterns of expression between the species, there are a few key differences. Notwithstanding the different rates of development between these two species, *MRF4* expression differed between the species throughout the neurula stage, as it was present in central somites in *B. lanceolatum* but not *B. floridae*. *MRF3* differed between the species in the region of the somite in which it was expressed in the late neurula stage. The different potential nonmyotomal expression of the *MRFs* is not conclusive at this point in time, but may demonstrate differences between the species in MRF expression in the orobranchial musculature. In total, with the conclusion that the amphioxus MRF expansion is ancestral to the cephalochordates, our results suggest that subfunctionalization of these five genes is also ancestral, and any differences between species are more recent, as the pattern of gene expression is generally consistent across the two species throughout the stages of development assessed here.

This expansion is consistent with the DDC hypothesis and also indicates a more complex regulatory network for myogenesis in the amphioxus than previously thought, as the number of MRFs has previously been underestimated. Although distinct expression patterns for *MRF1* and *MRF2a* were characterized previously (Schubert et al. 2003; Urano et al. 2003; Bertrand et al. 2011), now the full extent of the subfunctionalization of the amphioxus MRFs has been revealed. Furthermore, we found instances of possible nonsomitic expression of some of the MRFs which may represent an example of neofunctionalization, or perhaps the conserved use of the myogenic toolkit in nonmyomeric structures, akin to the formation of head musculature in vertebrates (Buckingham and Rigby 2014). The nonsomitic expression observed for *B. floridae MRF3* and *B. lanceolatum MRF3* and *MRF4* is consistent with a role in the developing striated muscle fibers in the orobranchial region (Yasui et al. 2014), which has not been reported in previous analyses of amphioxus MRF expression because these specific MRFs have not been assessed until now. The five-gene cluster of amphioxus illustrates multiple fates of duplicated genes in parallel with the already understood duplication and subfunctionalization in the vertebrates.

In other gene families, there are examples of independent duplications in the various chordate lineages. For instance, the urochordates have one *Pax3/7* gene, the pro-ortholog of vertebrate *Pax3* and *Pax7*, but amphioxus have a cluster of two genes, *Pax3/7a* and *Pax3/7b* (Barton-Owen et al. 2018), indicating that the ancestral *Pax3/7* gene underwent a tandem duplication in the amphioxus ancestor after divergence from the lineage leading to the Olfactores. *Pax3* and *Pax7* are upstream regulators of the MRFs in vertebrate myogenesis (Moncaut et al. 2013; Buckingham and Rigby 2014; Buckingham 2017), which suggests there may be multiple levels of increased complexity driving amphioxus myogenesis that have previously been underestimated. Independent expansions were also observed for the amphioxus opsin genes (Holland et al. 2008), amphioxus globin genes (Storz et al. 2011), and *Ciona* T-box genes (Dehal et al. 2002), among many others (Minguillón et al. 2002). These examples show how the one-to-four expectation can be obscured not only by losses but also by independent duplications, as we also see for the MRF family. Though the amphioxus body plan has remained similar to the presumed ancestral chordate and its genome is considered to exhibit remarkable stasis compared with other chordate groups (Paps et al. 2012), we find higher than expected levels of complexity in the amphioxus MRFs.

*MyoD* and its relatives play an important and highly conserved role in regulating muscle development across bilaterian animals. Through multiple instances of gene duplication in several animal lineages, these genes are linked to increased complexity in the myogenic regulatory network. This is particularly notable in the vertebrates, where the four vertebrate MRFs were thought to display the typical “one-to-four” ohnologous relationship arising from the 2R WGDs. Instead, we find evidence for a tandem duplication that predates 2R and created the two distinct clades of vertebrate MRFs. In parallel, amphioxus, an invertebrate chordate, has undergone an independent expansion of its MRFs into a cluster of five linked and highly similar genes with distinct but overlapping expression patterns in myogenesis. These two events illustrate subfunctionalization of developmental genes following duplication where daughter genes are expressed as subsets of the ancestral gene, as has widely been assumed for most genes retained following duplication. Use of the early-branching chordate amphioxus sheds light on the invertebrate-to-vertebrate transition and demonstrates the role of duplication and subfunctionalization in the increasing complexity of vertebrates. More studies using these early-branching chordate lineages can further elucidate the fate of duplicated genes and their impact on complexity, especially those with important developmental roles and key morphological consequences, like the MRF family and other developmental toolkit genes.

## Materials and Methods

### Bioinformatics

#### Phylogeny

Amphioxus sequences were predicted using TBlastN (Altschul et al. 1990) searches of the genome assemblies of *B. floridae*, *B. belcheri*, and *B. lanceolatum*. The exon boundaries were confirmed by comparison of cDNA against genomic sequences. The genomic organization of the MRFs was determined by TBlastN against genomic assemblies, as well as long-range PCR of the intergenic region between *MRF2b* and *MRF4* of the *B. floridae* BAC clone (F = AGAGGAGGTCAGTAGAGGGGTACAGGTC and R = CTGGGACGACATGATGACAGCCG.).

Aside from the amphioxus sequences, MRF protein sequences from other species were taken from Ensembl (Zerbino et al. 2018), NCBI protein database, or Uniprot (The UniProt Consortium 2019), as well as other databases including Xenbase (Karimi et al. 2018), Echinobase (Cameron et al. 2009; Kudtarkar and Cameron 2017), BeetleBase (Wang et al. 2007), SalmoBase (Samy et al. 2017), FlyBase (Thurmond et al. 2019), WormBase (Harris et al. 2010), and ANISEED (Brozovic et al. 2018). For genomes where the MRFs have not been annotated, a TBlastN search of the transcriptome or whole-genome assembly was used to find MRFs, using the closest relative’s sequences available. Some genes were manually curated, based on the known structure of verified or predicted gene models and transcriptome data, as well as the known structure of MRFs. For instance, there were no annotation records of the *S. purpuratus MyoD2* orthologs in the genomes of *P. miniata* or *L. variegatus*, but these genes were predicted using alignments to sequences from *Acanthaster planci* (XP_022107336.1) and *S. purpuratus* (XP_011672159.1), respectively. Accession numbers and state of assembly/prediction for all sequences are listed in supplementary table 1, Supplementary Material online.

Alignment of the full coding sequence of the 106 MRFs from the 36 species included in the phylogeny was made in Jalview (Waterhouse et al. 2009) using MUSCLE (Edgar 2004) with default settings, and manually curated. A trimmed alignment, spanning human MyoD sequence from positions 78–186 contained the bHLH domain as well as conserved domains flanking it, and 19 residues from the highly conserved helix-III domain (Bergstrom and Tapscott 2001), when removing much of the long nonconserved sequence in between, was made for use in the ML and Bayesian tree inference. Both alignments were submitted to ProtTest (Abascal et al. 2005) to determine the best substitution model, the closest equivalent of which was used for each tree method. The full coding sequence alignment was used for the NJ method, but with a pairwise cut-off of 95%. NJ trees were run on MEGAX (Kumar et al. 2016) (1,000 bootstrap replicates, JTT substitution model, Gamma distribution of rates among sites, shape parameter = 0.77, homogeneous pattern among lineages, pairwise deletion cut-off 95%). ML trees were made using the trimmed alignment and were run with IQ-TREE (Nguyen et al. 2015) on the CIPRES science gateway (Miller et al. 2010), and the model was selected automatically with ModelFinder (Kalyaanamoorthy et al. 2017) (JTT+I+G4) with 1,000 bootstrap replicates (Hoang et al. 2018). Bayesian inference trees were also made using the trimmed alignment and were run using MrBayes 3.2.6 (Ronquist and Huelsenbeck 2003) on CIPRES as well (Miller et al. 2010) (500,000,000 generations, 500,000 print frequency, 50,000 sample frequency, 4 chains, rates invgamma, mixed aamodel, stoprule = yes, when 0.01). Appropriate burn-in was determined in Tracer v.1.7.7 (Rambaut et al. 2018) (5,000,000 generations), and the maximum clade credibility tree made using TreeAnnotator (http://beast.community/treeannotator, last accessed August 9, 2018) (MCC, median node heights). The final tree figure is the ML topology, with shared branch support values listed for major branches (ML/BI/NJ), and missing branch support values represented as dashes.

#### Synteny

A candidate chromosome for the human ghost locus was identified using the human genome paralogon database (McLysaght et al. 2002). This was then confirmed with the since-updated human genome (GrCh38.p12) on Genomicus v94.01 (Nguyen et al. 2018). This was used to generate a list of gene families shared across at least two of the three MRF loci, which was then pared down to those with four or fewer paralogs, in order to include only those with a probable 2R origin. From there, many paralogs of these genes were found on the inferred ghost locus, chromosome 19. This list was also applied to a subset of the vertebrates to compare across species, namely *Mus musculus*, *Gallus gallus*, *Lepisosteus oculatus*, and *Danio rerio*, and identify the 4- and 8-fold paralogy of the MRF and ghost loci in these species.

The synteny of the fish *MyoD1* and *MyoD2* loci was also examined by focusing on nearby neighbors of the respective loci, with both 3R ohnologs shared between the two loci and genes characteristic of each locus. Salmon orthologs of these genes were found using reciprocal BlastP (Altschul et al. 1990) searches against salmon proteins on NCBI using the zebrafish (or if there was not a zebrafish gene, the medaka) protein sequences as the first query. The locations were found using TBlastN searches of the salmon proteins against the salmon genome. To find all salmon-specific paralogs of the genes, the top two hits, rather than the top one hit, were used because of the 4R salmonid duplication.

Human–amphioxus synteny was assessed using reciprocal BLAST searches. The protein sequences of the gene models annotated to the MRF-bearing scaffold, scaffold 3 (FLLO01000004.1) in *B. lanceolatum* (http://amphiencode.github.io/Data/), as this scaffold is longer and better annotated than the other MRF-bearing scaffolds in *B. floridae* (scaffold 94) or *B. belcheri* (scaffold 50), was compiled. These proteins were queried against the NCBI database of all nonredundant reference human genome proteins using BlastP (Altschul et al. 1990). For each of the lancelet scaffold 3 proteins, the top hit human protein was then recorded and queried against a database of all *B. lanceolatum* proteins, and the top *B. lanceolatum* hit for each human protein was recorded. The two sets of results were compared, and reciprocal best hits were considered orthologs. The locations of these human proteins were downloaded using BIOMART (Smedley et al. 2015).

Counts of orthologs of lancelet MRF neighborhood genes located on the different human chromosomes were tested for significance using a cumulative probability binomial test (probabilities with replacement) and a one-tailed Barnard’s exact test (probabilities without replacement) (Barnard 1945). The total number of direct human orthologs of amphioxus genes on the MRF-bearing scaffold 3 found was 135, the total number of human protein-coding genes (PCG) is 20,313 (NCBI). For each chromosome, the ratio of the number of orthologs found on that chromosome (a) relative to the total number of orthologs (b = 135) was compared with the number of PCGs on that chromosome (c) relative to the total number of PCGs in the genome (d = 20,313), and *P* values were calculated using the Excel formula: 1-Binom.Dist(a, b,(c/d),TRUE), where TRUE determines the cumulative probability (left-tailed test), that is, the probability of there being at most that many successes (a) given that number of trials (b) and the probability of success (c/d); and the inverse (1-Binom.Dist()) gives the *P* value, or the probability that there could be more successes relative to the number of trials and the probability of success.

For the Barnard’s exact test, a 2×2 contingency table for each of the chromosomes with the number of orthologs on that chromosome, the number of orthologs on the other chromosomes, the number of nonorthologous PCGs on that chromosome and the number of nonorthologous PCGs on other chromosomes was used. The Barnard’s exact test was implemented in R (code downloaded from https://www.r-statistics.com/2010/02/barnards-exact-test-a-powerful-alternative-for-fishers-exact-test-implemented-in-r/, last accessed February 6, 2019).

The orthology of the MRF-bearing scaffolds in the three *Branchiostoma* spp. was assessed using reciprocal BlastP searches with the same approach as above. Due to the longer size of the *B. lanceolatum* scaffold (FLLO01000004.1: 8.7 Mb), we identified several scaffolds in each of the other species that had significantly more than expected orthologs to *B. lanceolatum* scaffold 3. These scaffolds in *B. floridae*, were namely 256 (0.2 Mb), 243 (3.1 Mb), 190 (2.3 Mb), 41 (4.4 Mb), 172 (1.6 Mb), 138 (2.6 Mb), and 94 (MRF, 3.5 Mb), which we then compared with the ancestral linkage groups associated with *B. floridae* scaffolds by Srivastava et al. (2008). The microsynteny of the amphioxus MRF loci was also assessed with the aim to determine the directionality of the MRF arrangement by comparing the immediate neighbors of the MRFs in *B. floridae* to the unannotated scaffolds in *B. lanceolatum* (FLLO01000004.1) and *B. belcheri* (NW_017804132.1), as well as the BAC clone from *B. floridae* (AC150407.2). We used the protein sequences of the predicted *B. floridae* genes neighboring the MRFs as queries in TBlastN searches against each of the different genomic sequences, with a score cut-off of 200.

### Expression

#### Amphioxus WMISH

We used the amphioxus protocol for whole-mount in situ hybridization (WMISH) described by Holland et al. (1994). All Florida amphioxus (*B. floridae*) used in this thesis were collected from Tampa Bay (FL) by Dr Tom Butts and Dr Peter Osborne in July–August 2006. Spawning and embryo collection were performed as described in Holland and Yu (2004) for in situ hybridization. Embryos at different developmental stages (ranging from 7-h postfertilization to 3-day larvae) were fixed either for 1 h at room temperature or overnight at 4 °C with 4% (m/v) paraformaldehyde in MOPS buffer (0.5 M NaCl, 2 mM MgSO_4_, 1 mM EGTA, 0.1 M morpholinopropanesulfonic acid buffer, pH 7.5). After fixation, embryos were washed several times in 70% ethanol and stored at −20 °C.

Embryos, larvae, juveniles, and adults of European amphioxus (*B. lanceolatum*) were collected at the Laboratoire Aragó in Banyuls-sur-mèr (France) from May 31 to June 5, 2010. Spawning of ripe amphioxus was induced by heat stimulation (Fuentes et al. 2007) and different embryonic developmental stages (gastrula, early neurula, midneurula, late neurula, and early larval stage) were collected at regular intervals and fixed in 4% (m/v) paraformaldehyde in MOPS buffer for 1 h at room temperature or at 4 °C overnight. After fixation, embryos for WMISH were washed three times in 70% ethanol and stored in 70% ethanol at −20 °C. Larvae (first gill slit stage) and juvenile amphioxus from previous spawnings were kindly provided by Dr Héctor Escrivà and Dr Stéphanie Bertrand. Both developmental stages were fixed and stored in 70% ethanol or PBT following the same procedure described for the embryonic stages. Late developmental stages (late neurula and early larval stage) were kindly donated by Dr Ildikó Somorjai to complete the amphioxus developmental series.

Note added in proof.—During the final revision stages of this manuscript a new assembly of the *Branchiostoma floridae* genome was published (Simakov et al. 2020). The organisation of the *B. floridae* MRF gene cluster in this new assembly is the same as that in the earlier assembly, thus requiring no change to our figure 4. Also, our preliminary synteny analyses, using this new *B. floridae* assembly in place of the *B. lanceolatum* assembly, require no changes to our conclusions. The *B. lanceolatum* scaffold used in the synteny analyses described here was 8.74Mb in length with approximately 595 automatically annotated genes, and so the new *B. floridae* chromosome-scale assembly, with the MRFs on a 17.1Mb chromosome, provides little in the way of extra resolution. 

## Supplementary Material

msaa147_supplementary_data

## References

[msaa147-B1] Abascal F, Zardoya R, Posada D. 2005 ProtTest: selection of best-fit models of protein evolution. Bioinformatics 21(9):2104–2105. 15647292 10.1093/bioinformatics/bti263

[msaa147-B2] Altschul SF, Gish W, Miller W, Myers EW, Lipman DJ. 1990 Basic local alignment search tool. J Mol Biol. 215(3):403–410. 2231712 10.1016/S0022-2836(05)80360-2

[msaa147-B3] Andrikou C, Iovene E, Rizzo F, Oliveri P, Arnone MI. 2013 Myogenesis in the sea urchin embryo: the molecular fingerprint of the myoblast precursors. Evodevo 4(1):33. 10.1186/2041-9139-4-33 PMC4175510 24295205

[msaa147-B4] Araki I, Terazawa K, Satoh N. 1996 Duplication of an amphioxus myogenic bHLH gene is independent of vertebrate myogenic bHLH gene duplication. Gene 171(2):231–236. 8666278 10.1016/0378-1119(96)00174-6

[msaa147-B5] Barnard GA. 1945 A new test for 2 × 2 tables. Nature 156(3954):177–177.

[msaa147-B6] Barton-Owen TB, Ferrier DEK, Somorjai I. 2018 Pax3/7 duplicated and diverged independently in amphioxus, the basal chordate lineage. Sci Rep. 8(1):1–11. 29925900 10.1038/s41598-018-27700-x PMC6010424

[msaa147-B7] Bergstrom DA, Tapscott SJ. 2001 Molecular distinction between specification and differentiation in the myogenic basic helix-loop-helix transcription factor family. Mol Cell Biol. 21(7):2404–2412. 11259589 10.1128/MCB.21.7.2404-2412.2001 PMC86873

[msaa147-B8] Bertrand S, Camasses A, Somorjai I, Belgacem MR, Chabrol O, Escande M-L, Pontarotti P, Escriva H. 2011 Amphioxus FGF signaling predicts the acquisition of vertebrate morphological traits. Proc Natl Acad Sci U S A. 108(22):9160–9165. 21571634 10.1073/pnas.1014235108 PMC3107284

[msaa147-B9] Bertrand S, Escriva H. 2011 Evolutionary crossroads in developmental biology: amphioxus. Development 138(22):4819–4830. 22028023 10.1242/dev.066720

[msaa147-B10] Blomme T, Vandepoele K, De Bodt S, Simillion C, Maere S, Van de Peer Y. 2006 The gain and loss of genes during 600 million years of vertebrate evolution. Genome Biol. 7(5):R43. 16723033 10.1186/gb-2006-7-5-r43 PMC1779523

[msaa147-B11] Bourque G, Pevzner PA, Tesler G. 2004 Reconstructing the genomic architecture of ancestral mammals: lessons from human, mouse, and rat genomes. Genome Res. 14(4):507–516. 15059991 10.1101/gr.1975204 PMC383294

[msaa147-B12] Bower NI, Johnston IA. 2010 Paralogs of Atlantic salmon myoblast determination factor genes are distinctly regulated in proliferating and differentiating myogenic cells. Am J Physiol Integr Comp Physiol. 298(6):R1615–R1626. 10.1152/ajpregu.00114.2010 20375265

[msaa147-B13] Braasch I, Gehrke AR, Smith JJ, Kawasaki K, Manousaki T, Pasquier J, Amores A, Desvignes T, Batzel P, Catchen J, et al 2016 The spotted gar genome illuminates vertebrate evolution and facilitates human-teleost comparisons. Nat Genet. 48(4):427–437. 26950095 10.1038/ng.3526 PMC4817229

[msaa147-B14] Brozovic M, Dantec C, Dardaillon J, Dauga D, Faure E, Gineste M, Louis A, Naville M, Nitta KR, Piette J, et al 2018 ANISEED 2017: extending the integrated ascidian database to the exploration and evolutionary comparison of genome-scale datasets. Nucleic Acids Res. 46(D1):D718–D725. 29149270 10.1093/nar/gkx1108 PMC5753386

[msaa147-B15] Buckingham M. 2017 Gene regulatory networks and cell lineages that underlie the formation of skeletal muscle. Proc Natl Acad Sci U S A. 114(23):5830–5837. 28584083 10.1073/pnas.1610605114 PMC5468682

[msaa147-B16] Buckingham M, Rigby P. 2014 Gene regulatory networks and transcriptional mechanisms that control myogenesis. Dev Cell. 28(3):225–238. 24525185 10.1016/j.devcel.2013.12.020

[msaa147-B0009117] Cameron RA Samanta M Yuan A He D Davidson E. 2009 SpBase: the sea urchin genome database and web site. *Nucleic Acids Res*. 37(Suppl 1):D750–D754. 19010966 10.1093/nar/gkn887 PMC2686435

[msaa147-B17] Carvajal JJ, Keith A, Rigby P. 2008 Global transcriptional regulation of the locus encoding the skeletal muscle determination genes *Mrf4* and *Myf5* Genes Dev. 22(2):265–276. 18198342 10.1101/gad.442408 PMC2192759

[msaa147-B18] Charbonnier F, Gaspera B, Della Armand A-S, Van der Laarse WJ, Launay T, Becker C, Gallien C-L, Chanoine C. 2002 Two myogenin-related genes are differentially expressed in *Xenopus laevis* myogenesis and differ in their ability to transactivate muscle structural genes. J Biol Chem. 277(2):1139–1147. 11684685 10.1074/jbc.M107018200

[msaa147-B19] Chen L, Krause M, Sepanski M, Fire A. 1994 The *Caenorhabditis elegans* MYOD homologue HLH-1 is essential for proper muscle function and complete morphogenesis. Development 120(6):1631–1641. 8050369 10.1242/dev.120.6.1631

[msaa147-B20] Craxton M. 2010 A manual collection of *Syt*, *Esyt, Rph3a, Rph3al, Doc2*, and *Dblc2* genes from 46 metazoan genomes – an open access resource for neuroscience and evolutionary biology. BMC Genomics 11(1):37. 20078875 10.1186/1471-2164-11-37 PMC2823689

[msaa147-B21] Davis RL, Weintraub H, Lassar AB. 1987 Expression of a single transfected cDNA converts fibroblasts to myoblasts. Cell 51(6):987–1000. 3690668 10.1016/0092-8674(87)90585-x

[msaa147-B22] Dehal P, Boore JL. 2005 Two rounds of whole genome duplication in the ancestral vertebrate. PLoS Biol. 3(10):e314. 16128622 10.1371/journal.pbio.0030314 PMC1197285

[msaa147-B23] Dehal P, Satou Y, Campbell RK, Chapman J, Degnan B, Tomaso A, De DB, Gregorio A, Di GM, Goodstein DM, et al 2002 The draft genome of *Ciona intestinalis*: insights into chordate and vertebrate origins. Science 298(5601):2157–2167. 12481130 10.1126/science.1080049

[msaa147-B24] Edgar RC. 2004 MUSCLE: multiple sequence alignment with high accuracy and high throughput. Nucleic Acids Res. 32(5):1792–1797. 15034147 10.1093/nar/gkh340 PMC390337

[msaa147-B25] Flood PR. 1967 Structure of the segmental trunk muscle in amphioxus – with notes on the course and “endings” of the so-called ventral root fibres. Z Zellforsch. 84(3):389–416. 4881202

[msaa147-B26] Force A, Lynch M, Pickett FB, Amores A, Yan Y, Postlethwait J. 1999 Preservation of duplicate genes by complementary, degenerative mutations. Genetics 151(4):1531–1545. 10101175 10.1093/genetics/151.4.1531 PMC1460548

[msaa147-B27] Fuentes M, Benito E, Bertrand S, Paris M, Mignardot A, Godoy L, Jimenez-Delgado S, Oliveri D, Candiani S, Hirsinger E, et al 2007 Insights into spawning behavior and development of the European amphioxus (*Branchiostoma lanceolatum*). J Exp Zool B Mol Dev Evol. 308B(4):484–493. 10.1002/jez.b.21179 17520703

[msaa147-B28] Harris TW, Antoshechkin I, Bieri T, Blasiar D, Chan J, Chen WJ, De La Cruz N, Davis P, Duesbury M, Fang R, et al 2010 WormBase: a comprehensive resource for nematode research. Nucleic Acids Res. 38(Suppl 1):D463–D467. 19910365 10.1093/nar/gkp952 PMC2808986

[msaa147-B29] Hoang DT, Chernomor O, von Haeseler A, Minh BQ, Vinh LS. 2018 UFBoot2: improving the ultrafast bootstrap approximation. Mol Biol Evol. 35(2):518–522. 29077904 10.1093/molbev/msx281 PMC5850222

[msaa147-B30] Hoegg S, Brinkmann H, Taylor JS, Meyer A. 2004 Phylogenetic timing of the fish-specific genome duplication correlates with the diversification of teleost fish. J Mol Evol. 59(2):190–203. 15486693 10.1007/s00239-004-2613-z

[msaa147-B31] Holland LZ, Albalat R, Azumi K, Benito-Gutiérrez È, Blow MJ, Bronner-Fraser M, Brunet F, Butts T, Candiani S, Dishaw LJ, et al 2008 The amphioxus genome illuminates vertebrate origins and cephalochordate biology. Genome Res. 18(7):1100–1111. 18562680 10.1101/gr.073676.107 PMC2493399

[msaa147-B32] Holland LZ, Yu J-K. 2004 Cephalochordate (Amphioxus) embryos: procurement, culture, and basic methods In: Ettensohn CA, Wray GA, Wessel GM, editors. 1st ed Development of sea urchins, Ascidians, and other invertebrate Deuterostomes: experimental approaches. San Diego: Elsevier Academic Press p. 195–215. 10.1016/s0091-679x(04)74009-1 15575608

[msaa147-B33] Holland PWH, Garcia-Fernàndez J, Williams NA, Sidow A. 1994 Gene duplications and the origins of vertebrate development. Dev Suppl. 1994:125–133. 7579513

[msaa147-B34] Innan H, Kondrashov F. 2010 The evolution of gene duplications: classifying and distinguishing between models. Nat Rev Genet. 11(2):97–108. 20051986 10.1038/nrg2689

[msaa147-B35] Izzi SA, Colantuono BJ, Sullivan K, Khare P, Meedel TH, Khare P, Colantuono BJ, Sullivan K, Izzi SA. 2013 Functional studies of the *Ciona intestinalis* myogenic regulatory factor reveal conserved features of chordate myogenesis. Dev Biol. 376(2):213–223. 23391688 10.1016/j.ydbio.2013.01.033 PMC3673305

[msaa147-B36] Jimenez-Delgado S, Pascual-Anaya J, Garcia-Fernandez J. 2009 Implications of duplicated cis-regulatory elements in the evolution of metazoans: the DDI model or how simplicity begets novelty. Brief Funct Genomics Proteomics. 8(4):266–275. 10.1093/bfgp/elp029 19651705

[msaa147-B37] Kalyaanamoorthy S, Minh BQ, Wong TKF, von Haeseler A, Jermiin LS. 2017 ModelFinder: fast model selection for accurate phylogenetic estimates. Nat Methods. 14(6):587–589. 28481363 10.1038/nmeth.4285 PMC5453245

[msaa147-B38] Karimi K, Fortriede JD, Lotay VS, Burns KA, Wang DZ, Fisher ME, Pells TJ, James-Zorn C, Wang Y, Ponferrada VG, et al 2018 Xenbase: a genomic, epigenomic and transcriptomic model organism database. Nucleic Acids Res. 46(D1):D861–D868. 29059324 10.1093/nar/gkx936 PMC5753396

[msaa147-B39] Kleinjan DA, Bancewicz RM, Gautier P, Dahm R, Schonthaler HB, Damante G, Seawright A, Hever AM, Yeyati PL, van Heyningen V, et al 2008 Subfunctionalization of duplicated zebrafish *pax6* genes by cis-regulatory divergence. PLoS Genet. 4(2):e29. 18282108 10.1371/journal.pgen.0040029 PMC2242813

[msaa147-B40] Kudtarkar P, Cameron RA. 2017 Echinobase: an expanding resource for echinoderm genomic information. Database 2017:1–9. 10.1093/database/bax074 PMC5737241 29220460

[msaa147-B41] Kumar S, Stecher G, Tamura K. 2016 MEGA7: molecular evolutionary genetics analysis version 7.0 for bigger datasets. Mol Biol Evol. 33(7):1870–1874. 27004904 10.1093/molbev/msw054 PMC8210823

[msaa147-B42] Lien S, Koop BF, Sandve SR, Miller JR, Kent MP, Nome T, Hvidsten TR, Leong JS, Minkley DR, Zimin A, et al 2016 The Atlantic salmon genome provides insights into rediploidization. Nature 533(7602):200–205. 27088604 10.1038/nature17164 PMC8127823

[msaa147-B43] Lynch M, Conery JS. 2000 The evolutionary fate and consequences of duplicate genes. Science 290(5494):1151–1155. 11073452 10.1126/science.290.5494.1151

[msaa147-B44] MacCarthy T, Bergman A. 2007 The limits of subfunctionalization. BMC Evol Biol. 7(1):213. 17988397 10.1186/1471-2148-7-213 PMC2213666

[msaa147-B45] MacKintosh C, Ferrier DEK. 2018 Recent advances in understanding the roles of whole genome duplications in evolution. F1000Res. 6:1623. 10.12688/f1000research.11792.1 PMC5590085 28928963

[msaa147-B46] Macqueen DJ, Johnston IA. 2006 A novel salmonid *myoD* gene is distinctly regulated during development and probably arose by duplication after the genome tetraploidization. FEBS Lett. 580(21):4996–5002. 16930594 10.1016/j.febslet.2006.08.016

[msaa147-B47] Macqueen DJ, Johnston IA. 2008 An update on *MyoD* evolution in teleosts and a proposed consensus nomenclature to accommodate the tetraploidization of different vertebrate genomes. PLoS One 3(2):e1567. 18253507 10.1371/journal.pone.0001567 PMC2215776

[msaa147-B48] Marlétaz F, Firbas PN, Maeso I, Tena JJ, Bogdanovic O, Perry M, Wyatt CDR, de la Calle-Mustienes E, Bertrand S, Burguera D, et al 2018 Amphioxus functional genomics and the origins of vertebrate gene regulation. Nature 564(7734):64–70. 30464347 10.1038/s41586-018-0734-6 PMC6292497

[msaa147-B49] McLysaght A, Hokamp K, Wolfe KH. 2002 Extensive genomic duplication during early chordate evolution. Nat Genet. 31(2):200–204. 12032567 10.1038/ng884

[msaa147-B50] Meedel TH, Farmer SC, Lee JJ. 1997 The single *MyoD* family gene of *Ciona intestinalis* encodes two differentially expressed proteins: implications for the evolution of chordate muscle gene regulation. Development 1721:1711–1721. 10.1242/dev.124.9.1711 9165119

[msaa147-B51] Meedel TH, Lee JJ, Whittaker JR. 2002 Muscle development and lineage-specific expression of *CiMDF*, the *MyoD*-family gene of *Ciona intestinalis*. Dev Biol. 241(2):238–246. 11784108 10.1006/dbio.2001.0511

[msaa147-B52] Megeney LA, Rudnicki MA. 1995 Determination versus differentiation and the MyoD family of transcription factors. Biochem Cell Biol. 73(9–10):723–732. 8714693 10.1139/o95-080

[msaa147-B53] Meyer A, Schartl M. 1999 Gene and genome duplications in vertebrates: the one-to-four (-to-eight in fish) rule and the evolution of novel gene functions. Curr Opin Cell Biol. 11(6):699–704. 10600714 10.1016/s0955-0674(99)00039-3

[msaa147-B54] Miller MA, Pfeiffer W, Schwartz T. 2010 Creating the CIPRES Science Gateway for inference of large phylogenetic trees. In: 2010 Gateway Computing Environments Workshop (GCE). New Orleans (LA): IEEE. p. 1–8.

[msaa147-B55] Minguillón C, Ferrier DEK, Cebrián C, Garcia-Fernàndez J. 2002 Gene duplications in the prototypical cephalochordate amphioxus. Gene 287(1–2):121–128. 11992730 10.1016/s0378-1119(01)00828-9

[msaa147-B56] Misquitta L, Paterson BM. 1999 Targeted disruption of gene function in Drosophila by RNA interference (RNA-i): a role for nautilus in embryonic somatic muscle formation. Proc Natl Acad Sci U S A. 96(4):1451–1456. 9990044 10.1073/pnas.96.4.1451 PMC15483

[msaa147-B57] Moncaut N, Rigby PWJ, Carvajal JJ. 2013 Dial M(RF) for myogenesis. FEBS J. 280(17):3980–3990. 23751110 10.1111/febs.12379

[msaa147-B58] Nakatani Y, Takeda H, Kohara Y, Morishita S. 2007 Reconstruction of the vertebrate ancestral genome reveals dynamic genome reorganization in early vertebrates. Genome Res. 17(9):1254–1265. 17652425 10.1101/gr.6316407 PMC1950894

[msaa147-B59] Nguyen L-T, Schmidt HA, von Haeseler A, Minh BQ. 2015 IQ-TREE: a fast and effective stochastic algorithm for estimating maximum-likelihood phylogenies. Mol Biol Evol. 32(1):268–274. 25371430 10.1093/molbev/msu300 PMC4271533

[msaa147-B60] Nguyen NTT, Vincens P, Roest Crollius H, Louis A. 2018 Genomicus 2018: karyotype evolutionary trees and on-the-fly synteny computing. Nucleic Acids Res. 46(D1):D816–D822. 29087490 10.1093/nar/gkx1003 PMC5753199

[msaa147-B61] Paps J, Holland PWH, Shimeld SM. 2012 A genome-wide view of transcription factor gene diversity in chordate evolution: less gene loss in amphioxus? Brief Funct Genomics. 11(2):177–186. 22441554 10.1093/bfgp/els012

[msaa147-B62] Peachey LD. 1961 Structure of the longitudinal body muscles of amphioxus. J Cell Biol. 10(4):159–176. 10.1083/jcb.10.4.159 PMC2225103 13733733

[msaa147-B63] Perry RLS, Rudnicki MA. 2000 Molecular mechanisms regulating myogenic determination and differentiation Front Biosci. 5(3):D750–D767. 10966875 10.2741/perry

[msaa147-B64] Prince VE, Pickett FB. 2002 Splitting pairs: the diverging fates of duplicated genes. Nat Rev Genet. 3(11):827–837. 12415313 10.1038/nrg928

[msaa147-B65] Putnam NH, Butts T, Ferrier DEK, Furlong RF, Hellsten U, Kawashima T, Robinson-Rechavi M, Shoguchi E, Terry A, Yu J-K, et al 2008 The amphioxus genome and the evolution of the chordate karyotype. Nature 453(7198):1064–1071. 18563158 10.1038/nature06967

[msaa147-B11290617] Rambaut A Drummond AJ Xie D Baele G Suchard MA. 2018 Posterior Summarization in Bayesian Phylogenetics Using Tracer 1.7. *Syst Biol*. 67(5):901–904. 29718447 10.1093/sysbio/syy032 PMC6101584

[msaa147-B66] Ratcliffe LE, Asiedu EK, Pickett CJJ, Warburton MA, Izzi SA, Meedel TH. 2019 The Ciona myogenic regulatory factor functions as a typical MRF but possesses a novel N-terminus that is essential for activity. Dev Biol. 448(2):210–225. 30365920 10.1016/j.ydbio.2018.10.010 PMC6478573

[msaa147-B67] Rawls A, Morris JH, Rudnicki M, Braun T, Arnold HH, Klein WH, Olson EN. 1995 Myogenin’s functions do not overlap with those of MyoD or Myf-5 during mouse embryogenesis. Dev Biol. 172(1):37–50. 7589813 10.1006/dbio.1995.0004

[msaa147-B68] Ronquist F, Huelsenbeck JP. 2003 MrBayes 3: Bayesian phylogenetic inference under mixed models. Bioinformatics 19(12):1572–1574. 12912839 10.1093/bioinformatics/btg180

[msaa147-B69] Rudnicki MA, Braun T, Arnold H-H, Jaenisch R. 1993 Targeted inactivation of the muscle regulatory genes *Myf-5* and *MyoD*: effect on muscle and skeletal development In: Wagner EF, Theuring F, editors. Transgenic animals as model systems for human diseases. Heidelberg (Berlin): Springer p. 143–151.

[msaa147-B70] Samy JKA, Mulugeta TD, Nome T, Sandve SR, Grammes F, Kent MP, Lien S, Våge DI. 2017 SalmoBase: an integrated molecular data resource for Salmonid species. BMC Genomics 18(1):482. 28651544 10.1186/s12864-017-3877-1 PMC5485693

[msaa147-B71] Schnapp E, Pistocchi AS, Karampetsou E, Foglia E, Lamia CL, Cotelli F, Cossu G. 2009 Induced early expression of *mrf4* but not *myog* rescues myogenesis in the *myod/myf5* double-morphant zebrafish embryo. J Cell Sci. 122(4):481–488. 19193870 10.1242/jcs.038356

[msaa147-B72] Schubert M, Meulemans D, Bronner-Fraser M, Holland LZ, Holland ND. 2003 Differential mesodermal expression of two amphioxus *MyoD* family members (*AmphiMRF1* and *AmphiMRF2*). Gene Expr Patt. 3(2):199–202. 10.1016/s1567-133x(02)00099-6 12711549

[msaa147-B73] Shimeld SM. 1999 Gene function, gene networks and the fate of duplicated genes. Semin Cell Dev Biol. 10(5):549–553. 10597639 10.1006/scdb.1999.0336

[msaa147-B74] Shimeld SM, Holland P. 2000 Vertebrate innovations. Proc Natl Acad Sci U S A. 97(9):4449–4452. 10781042 10.1073/pnas.97.9.4449 PMC34320

[msaa147-B4798688] Simakov O Marlétaz F Yue J-X O’Connell B Jenkins J Brandt A Calef R Tung C-H Huang T-K Schmutz J, et al. 2020 Deeply conserved synteny resolves early events in vertebrate evolution. *Nat Ecol Evol*. 4(6):820–830. 32313176 10.1038/s41559-020-1156-z PMC7269912

[msaa147-B75] Smedley D, Haider S, Durinck S, Pandini L, Provero P, Allen J, Arnaiz O, Awedh MH, Baldock R, Barbiera G, et al 2015 The BioMart community portal: an innovative alternative to large, centralized data repositories. Nucleic Acids Res. 43(W1):W589–W598. 25897122 10.1093/nar/gkv350 PMC4489294

[msaa147-B76] Somorjai IML, Bertrand S, Camasses A, Haguenauer A, Escriva H. 2008 Evidence for stasis and not genetic piracy in developmental expression patterns of *Branchiostoma lanceolatum* and *Branchiostoma floridae*, two amphioxus species that have evolved independently over the course of 200 Myr. Dev Genes Evol. 218(11–12):703–713. 18843503 10.1007/s00427-008-0256-6

[msaa147-B77] Srivastava M, Begovic E, Chapman J, Putnam NH, Hellsten U, Kawashima T, Kuo A, Mitros T, Salamov A, Carpenter ML, et al 2008 The Trichoplax genome and the nature of placozoans. Nature 454(7207):955–960. 18719581 10.1038/nature07191

[msaa147-B78] Storz JF, Opazo JC, Hoffmann FG. 2011 Phylogenetic diversification of the globin gene superfamily in chordates. IUBMB Life 63(5):313–322. 21557448 10.1002/iub.482 PMC4399706

[msaa147-B79] Tan X, Du SJ. 2002 Differential expression of two *MyoD* genes in fast and slow muscles of gilthead seabream (*Sparus aurata*). Dev Genes Evol. 212(5):207–217. 12070611 10.1007/s00427-002-0224-5

[msaa147-B80] Tan X, Zhang PJ, Du SJ. 2014 Evolutionary aspects of a new MyoD gene in amphioxus (*Branchiostoma belcheri*) and its promoter specificity in skeletal and cardiac muscles. Cell Mol Biol. 69(9):1210–1221.

[msaa147-B81] The UniProt Consortium. 2019 UniProt: a worldwide hub of protein knowledge. Nucleic Acids Res. 47(D1):D506–D515. 30395287 10.1093/nar/gky1049 PMC6323992

[msaa147-B82] Thurmond J, Goodman JL, Strelets VB, Attrill H, Gramates LS, Marygold SJ, Matthews BB, Millburn G, Antonazzo G, Trovisco V, the FlyBase Consortium, et al 2019 FlyBase 2.0: the next generation. Nucleic Acids Res. 47(D1):D759–D765. 30364959 10.1093/nar/gky1003 PMC6323960

[msaa147-B83] Urano A, Suzuki MM, Zhang P, Satoh N, Satoh G. 2003 Expression of muscle-related genes and two *MyoD* genes during amphioxus notochord development. Evol Dev. 5(5):447–458. 12950624 10.1046/j.1525-142x.2003.03051.x

[msaa147-B84] Wagner A. 1998 The fate of duplicated genes: loss or new function? BioEssays 20(10):785–788. 10200118 10.1002/(SICI)1521-1878(199810)20:10<785::AID-BIES2>3.0.CO;2-M

[msaa147-B85] Wang L, Wang S, Li Y, Paradesi MSR, Brown SJ. 2007 BeetleBase: the model organism database for *Tribolium castaneum*. Nucleic Acids Res. 35(Database):D476–D479. 17090595 10.1093/nar/gkl776 PMC1669707

[msaa147-B86] Waterhouse AM, Procter JB, Martin DMA, Clamp M, Barton GJ. 2009 Jalview Version 2-A multiple sequence alignment editor and analysis workbench. Bioinformatics 25(9):1189–1191. 19151095 10.1093/bioinformatics/btp033 PMC2672624

[msaa147-B87] Wolfe K. 2000 Robustness – it’s not where you think it is. Nat Genet. 25(1):3–4. 10802639 10.1038/75560

[msaa147-B88] Yasui K, Kaji T, Morov AR, Yonemura S. 2014 Development of oral and branchial muscles in lancelet larvae of *Branchiostoma japonicum*. J Morphol. 275(4):465–477. 24301696 10.1002/jmor.20228

[msaa147-B89] Yuan J, Zhang S, Liu Z, Luan Z, Hu G. 2003 Cloning and phylogenetic analysis of an amphioxus myogenic bHLH gene *AmphiMDF* Biochem Biophys Res Commun. 301(4):960–967. 12589806 10.1016/s0006-291x(03)00081-0

[msaa147-B90] Zammit PS. 2017 Function of the myogenic regulatory factors Myf5, MyoD, Myogenin and MRF4 in skeletal muscle, satellite cells and regenerative myogenesis. Semin Cell Dev Biol. 72:19–32. 29127046 10.1016/j.semcdb.2017.11.011

[msaa147-B91] Zerbino DR, Achuthan P, Akanni W, Amode MR, Barrell D, Bhai J, Billis K, Cummins C, Gall A, Girón CG, et al 2018 Ensembl 2018. Nucleic Acids Res. 46(D1):D754–D761. 29155950 10.1093/nar/gkx1098 PMC5753206

[msaa147-B92] Zhang J. 2003 Evolution by gene duplication: an update. Trends Ecol Evol. 18(6):292–298.

