# Supplementary material for: More Than One-to-Four via 2R: Evidence of an Independent Amphioxus Expansion and Two-Gene Ancestral Vertebrate State for MyoD-Related Myogenic Regulatory Factors (MRFs)
Source: Mol Biol Evol. 2020 Jun 10;37(10):2966–82. doi: 10.1093/molbev/msaa147 (PMC7530620; doi:10.1093/molbev/msaa147)
Supplement: msaa147_supplementary_data [file msaa147_supplementary_data.zip › supplementary_information.pdf]

**SUPPLEMENTARY INFORMATION**

Contents


1. Amphioxus *MRF* ambiguities

2. *Myf7* annotation

3. Phylogenies
   a. Bayesian Inference
   b. Maximum likelihood
   c. Neighbour-joining
   d. Maximum likelihood with cyclostomes
   e. Maximum likelihood without cyclostomes

4. Alignments
   a. Full-length *MRF* alignment
   b. Trimmed *MRF* alignment

5. Synteny of *MRF* and ghost loci
   a. *MyoD*
   b. *Myog*
   c. *Myf5-Myf6*
   d. ghost *MRF*

6. Synteny between human and amphioxus
   a. Human *MRF*-linked orthologues of amphioxus *MRF*-linked genes peaks at the *MRF* locus
   b. Chromosome locations of 1:1 orthologues of amphioxus *MRF*-linked genes
   c. Barnard's exact test
   d. Binomial test
   e. Ancestral linkage group 2
   f. Ancestral linkage groups on chromosomes 1, 11, 12, and 19

7. WMISH of amphioxus MRFs in *B. floridae* and *B. lanceolatum* embryos
   a. Gastrulae
   b. Early-mid neurulae
   c. Late neurulae
   d. Early larvae
   e. Late larvae
   f. Probes
   g. Extended description

1. Amphioxus *MRF* ambiguities

| Author, year | Species | 1 | 2a | 2b | 3 | 4 | Exp | New | Total |
|---|---|---|---|---|---|---|---|---|---|
| Araki et al. 1996 | *B. floridae* | ● | ● (red) ? | | | | – | 2 | 2 |
| Schubert et al. 2003 | | ● | ● | | | | + | 0 | 2 |
| Putnam et al. 2008 | | ● | ● | ○ | ○ | ○ (red) | – | 3 | 5 |
| Current authors, 2020 | | ● | ● | ● | ● | ● | + | 0 | 5 |
| Yuan et al. 2003 | *B. belcheri* | | | ● | | | – | 1 | 1 |
| Urano et al. 2003 | | ● | ● | | | | + | 2 | 3 |
| Tan et al. 2014 | | | | | ● | | – | 1 | 4 |
| Somorjai et al. 2008 | *B. lanceolatum* | ● (red) | | | | | + | 1 | 1 |
| Bertrand et al. 2011 | | ● (red) | | | | | + | 1 | 1 |
| Aldea et al. 2019 | | | ● | ● | | ● | – | 3 | 4 |
| Marlétaz et al. 2018 | | ○* (red) | ○ | ○* (red) | ○ | ○ | – | 4 | 4 |
| Current authors, 2020 | | ● | ● | ● | ● | ● | + | 1 | 5 |

Published *MRF* sequences for the three *Branchiostoma* spp., which genes were described, whether their expression was analysed, the number of genes described in that study, and the total number of genes described at that point. Filled circles denote experimentally confirmed sequences, while empty circles are automatically predicted from the genome sequence; red-coloured circles are partial sequences. The question mark denotes a partial sequence that is the bHLH domain from either *MRF2a* or *MRF2b*, but which of these genes it is cannot be determined because they are identical in this region. The star highlights two genes that have been combined into one gene model in the *B. lanceolatum* annotation (Marlétaz *et al.* 2018).

Accession numbers and sources:

*BfloMRF1*: D50101 & *BfloMRF2*: D50102 (Araki et al. 1996)
*BfloMRF1*: AY154744 & *BfloMRF2*: AY154745 (Schubert et al. 2003)
*BfloMRF1*: XP_002604822.1, *BfloMRF2a*: XP_002604824.1, *BfloMRF2b*: XP_002604821.1, *BfloMRF3*: XP_002604823.1, & *BfloMRF4*: XP_002604825.1 (Putnam et al. 2008)
*BbelMRF2b*: AY066009 (Yuan et al. 2003)
*BbelMRF1*: AB092415 & *BbelMRF2a*: AB092416; *BbelMRF3*: AY313170.1 (Tan et al. 2014)
*BlanMRF1*: ACE79716.1 (Somorjai et al. 2008)
*BlanMRF2a*: MF287281.1, *BlanMRF2b*: MF287279.1, & *BlanMRF4*: MF287280.1 (called MRF3) (Aldea et al. 2019)
BlanMRF1/BlanMRF2b: BL15168_cuf4/cuf0, *BlanMRF2a*: BL21563, *BlanMRF3*: BL24962, *BlanMRF4*: BL08615 (Marlétaz et al. 2018).

**References**

Aldea D, Subirana L, Keime C, Meister L, Maeso I, Marcellini S, Gomez-Skarmeta JL, Bertrand S, Escriva H. 2019. Genetic regulation of amphioxus somitogenesis informs the evolution of the vertebrate head mesoderm. Nat Ecol Evol. 3(8):1233–1240. doi:10.1038/s41559-019-0933-z.

Araki I, Terazawa K, Satoh N. 1996. Duplication of an amphioxus myogenic bHLH gene is independent of vertebrate myogenic bHLH gene duplication. Gene. 171(2):231–236. doi:10.1016/0378-1119(96)00174-6.

Bertrand S, Camasses A, Somorjai I, Belgacem MR, Chabrol O, Escande M-L, Pontarotti P, Escriva H. 2011. Amphioxus FGF signaling predicts the acquisition of vertebrate morphological traits. Proc Natl Acad Sci U S A. 108(22):9160–5. doi:10.1073/pnas.1014235108.

Marlétaz F, Firbas PN, Maeso I, Tena JJ, Bogdanovic O, Perry M, Wyatt CDR, de la Calle-Mustienes E, Bertrand S, Burguera D, et al. 2018. Amphioxus functional genomics and the origins of vertebrate gene regulation. Nature. 564(7734):64–70. doi:10.1038/s41586-018-0734-6.

Putnam NH, Butts T, Ferrier DEK, Furlong RF, Hellsten U, Kawashima T, Robinson-Rechavi M, Shoguchi E, Terry A, Yu J-K, et al. 2008. The amphioxus genome and the evolution of the chordate karyotype. Nature. 453(7198):1064–1071. doi:10.1038/nature06967.

Schubert M, Meulemans D, Bronner-Fraser M, Holland LZ, Holland ND. 2003. Differential mesodermal expression of two amphioxus MyoD family members (AmphiMRF1 and AmphiMRF2). Gene Expr Patterns. 3(2):199–202. doi:10.1016/S1567-133X(02)00099-6.

Somorjai I, Bertrand S, Camasses A, Haguenauer A, Escriva H. 2008. Evidence for stasis and not genetic piracy in developmental expression patterns of Branchiostoma lanceolatum and Branchiostoma floridae, two amphioxus species that have evolved independently over the course of 200 Myr. Dev Genes Evol. 218(11–12):703–713. doi:10.1007/s00427-008-0256-6.

Tan X, Zhang PJ, Du SJ. 2014. Evolutionary aspects of a new MyoD gene in amphioxus (Branchiostoma belcheri) and its promoter specificity in skeletal and cardiac muscles. Biol. 69(9):1210–1221. doi:10.2478/s11756-014-0427-z.

Urano A, Suzuki MM, Zhang P, Satoh N, Satoh G. 2003. Expression of muscle-related genes and two MyoD genes during amphioxus notochord development. Evol Dev. 5(5):447–458. doi:10.1046/j.1525-142X.2003.03051.x.

Yuan J, Zhang S, Liu Z, Luan Z, Hu G. 2003. Cloning and phylogenetic analysis of an amphioxus myogenic bHLH gene AmphiMDF. Biochem Biophys Res Commun. 301(4):960–967. doi:10.1016/S0006-291X(03)00081-0.

2. *Myf7* annotation

| Species | Gene | NCBI GenBank | Ensembl | UniProt |
|---|---|---|---|---|
| *Latimeria chalumnae* | *Myf7* | "myogenic factor 6-like" XP_005990555.1 | "MYF5" ENSLACG00000014612 | "Myogenic factor" H3B3V9 |
| | *Myf5* | "MYF5" XP_005996064.1 | -none- | -none- |
| | *Myf6* | "MYF6" XP_005996063.1 | "MYF6" ENSLACG00000010731 | "MYF6" H3ARC3 |
| *Lepisosteus oculatus* | *Myf7* | "myogenic factor 6-like" XP_015193831.1 | "Myogenic factor 6 like" ENSLOCG00000002142 | "Uncharacterized" W5M281 |
| | *Myf5* | "MYF5" XP_006633605.1 | "MYF6" ENSLOCG00000016302 | -none- |
| | *Myf6* | "MYF6" XP_006633604.1 | | "Myogenic factor" W5NHL0 |
| *Acipenser ruthenus* | *Myf7* | "MYF6" RXM31768.1; EOD39_6691 | -none- | -none- |
| | *Myf5* | -none- sc1291: 227,344-229,226 | -none- | -none- |
| | *Myf6* | -none- sc1291: 218,425-227,748 | -none- | -none- |

Protein sequences of the three *Myf7* genes; exons separated by dashes (-), bHLH domain in bold.

- *Latimeria chalumnae Myf7* protein sequence:

MELYEANSCFQDQNYFNNENTSVLTNYDDFIPLEEGKDSEESLKASSVCTAIEEHVFAPPGFHHTTGQCLLWACKICKRKS**VTMDRR**<br>**KAATLRERRRLKRVNEAFETLKRKTVPNPNQQLPKVEILRSAIQYIARLQSLLKS**LNEQNAVSVNRNVSCQSNSQ-<br>HGNDCPWGSSSVTNWEQEENKHSSFAYSGHKEA-GTKDSTGAASLQCLSSIVDSISLQE*

Location: JH126627.1: 1,061,960-1,066,281<br>Confirmed with cDNA sequences from three muscle transcriptome tissue samples:<br>GAAA01063793, GAAA01079678, and GAAA01054457.

- *Lepisosteus oculatus Myf7* protein sequence:

MQHPHFVCANERHVTTSKDSGDDEAEPQVCSRMNSPTEEHIGPSPRPGLSERQCMVWACGVYKRRPS**QTDRRRAATLRERKRLK**<br>**RVNEAFEALKQKTVPNPKRRLSKVEILRHAIQHIMRLQSLLGA**AREQGAVPGSRIATCHCGPQPECCADQSHHHSVPY-*EXON3?*-<br>*

Location: LG27: 5,111,582-5,113,950

- *Acipenser ruthenus Myf7* protein sequence:

MELSEPLGYYFRDQEYLGNENVTPVFINYDEQVVSEDDGRLPKEGPKAFSNPAVPEEHVFAPPGFHHRAGPCLLWACTVCKRKS**VT**<br>**MDRRKAATQRERRRLKRINEGFEALKRKSVPNPNQRLPKVEILRSAIQYITRLQTLLRT**LDDQEAVPIDRKDTHHSSLQVGD-<br>*EXON2?*-PVVLISVSSVYPAGTPGSSGTASHQGLSSIVDSISVEDTDST*

Location: sc347: 537,345-539,610 (SCEB01215015.1)

## 3. Phylogenies

### a. BI

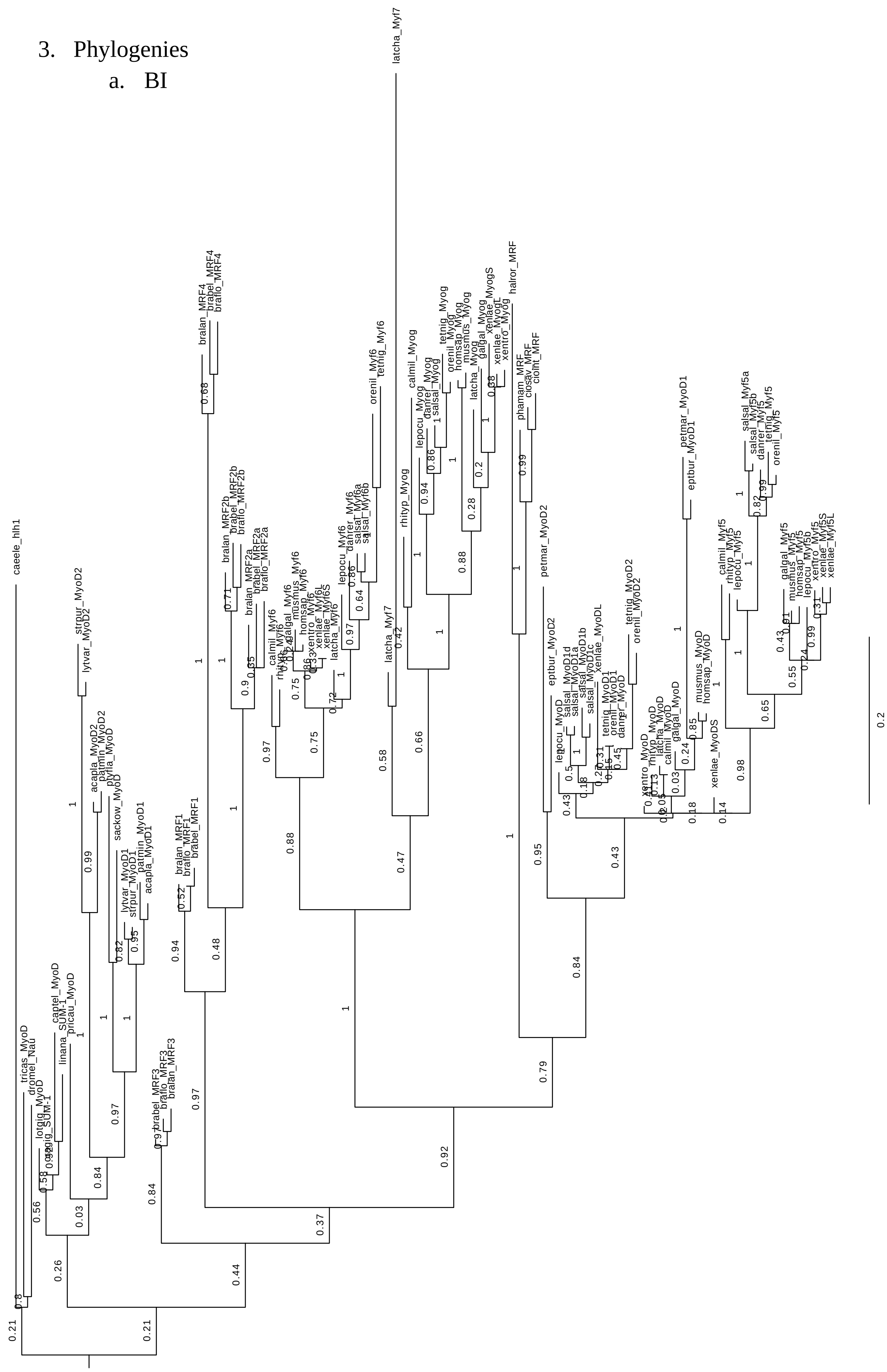

b. ML

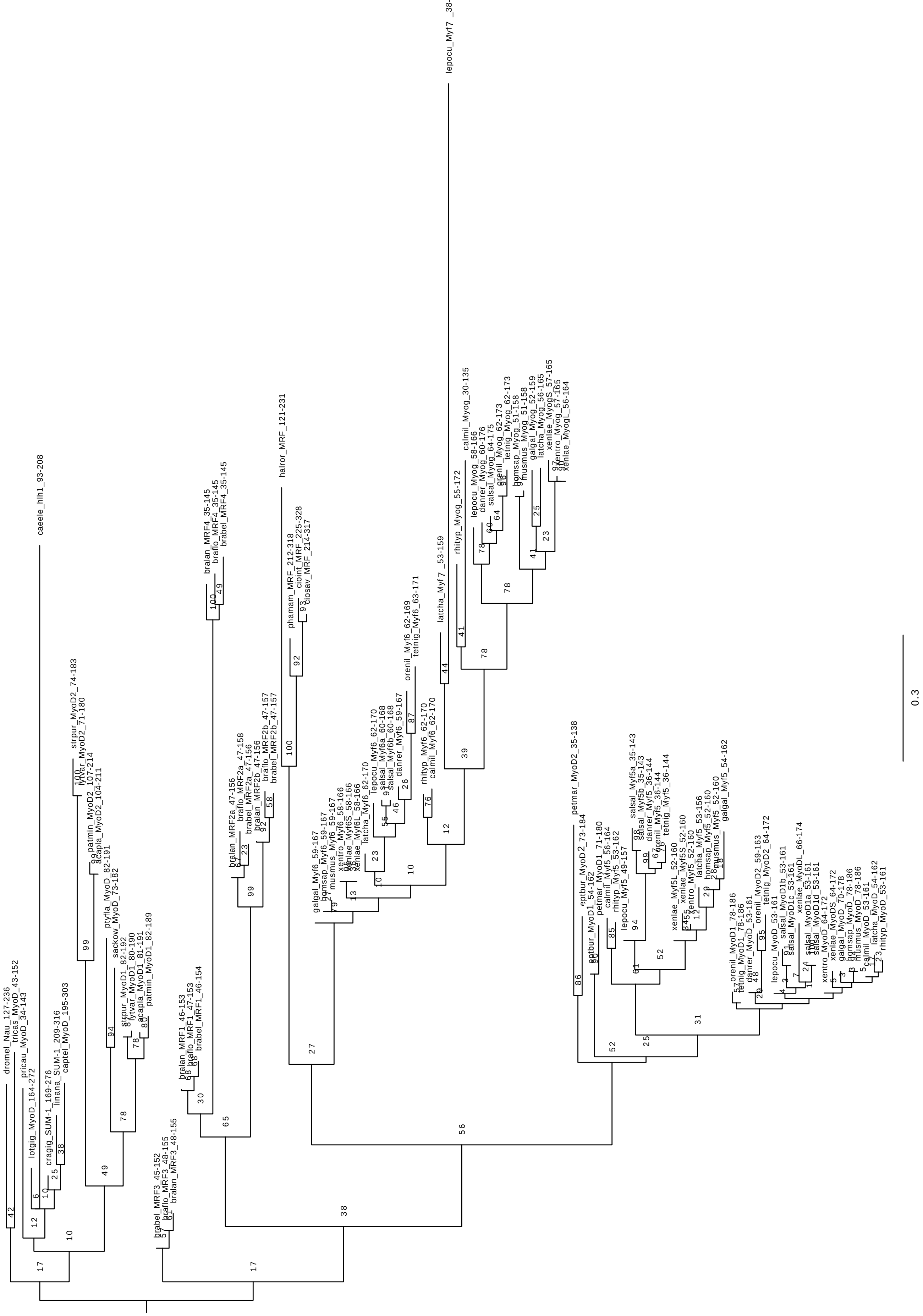

lepocu_Myf7 _38-127
caeele_hlh1_93-208
bralan_MRF4_35-145
braflo_MRF4_35-145
brabel_MRF4_35-145
halror_MRF_121-231
phamam_MRF_212-318
cioint_MRF_225-328
ciosav_MRF_214-317
calmil_Myog_30-135
lepocu_Myog_58-166
danrer_Myog_60-176
salsal_Myog_64-175
orenil_Myog_62-173
tetnig_Myog_62-173
homsap_Myog_51-158
musmus_Myog_51-158
galgal_Myog_52-159
latcha_Myog_56-165
xenlae_MyogS_57-165
xentro_Myog_57-165
xenlae_MyogL_56-164
rhityp_Myog_55-172
latcha_Myf7_53-159
orenil_Myf6_62-169
tetnig_Myf6_63-171
rhityp_Myf6_62-170
calmil_Myf6_62-170
strpur_MyoD2_74-183
lytvar_MyoD2_71-180
patmin_MyoD2_107-214
acapla_MyoD2_104-211
ptyfla_MyoD_82-191
sackow_MyoD_73-182
strpur_MyoD1_82-192
lytvar_MyoD1_80-190
acapla_MyoD1_81-191
patmin_MyoD1_82-189
dromel_Nau_127-236
tricas_MyoD_43-152
pricau_MyoD_34-143
lotgig_MyoD_164-272
cragig_SUM-1_169-276
linana_SUM-1_209-316
captel_MyoD_195-303
brabel_MRF3_45-152
braflo_MRF3_48-155
bralan_MRF3_48-155
bralan_MRF1_46-153
braflo_MRF1_47-153
brabel_MRF1_46-154
bralan_MRF2a_47-156
braflo_MRF2a_47-158
brabel_MRF2a_47-156
bralan_MRF2b_47-156
braflo_MRF2b_47-157
brabel_MRF2b_47-157
galgal_Myf6_59-167
homsap_Myf6_59-167
musmus_Myf6_59-167
xentro_Myf6_58-166
xenlae_Myf6S_58-166
xenlae_Myf6L_58-166
latcha_Myf6_62-170
lepocu_Myf6_62-170
salsal_Myf6a_60-168
salsal_Myf6b_60-168
danrer_Myf6_59-167
petmar_MyoD2_35-138
eptbur_MyoD2_73-184
eptbur_MyoD1_54-162
petmar_MyoD1_71-180
calmil_Myf5_56-164
rhityp_Myf5_53-162
lepocu_Myf5_49-157
salsal_Myf5a_35-143
salsal_Myf5b_35-143
danrer_Myf5_36-144
orenil_Myf5_36-144
tetnig_Myf5_36-144
xenlae_Myf5L_52-160
xenlae_Myf5S_52-160
xentro_Myf5_52-160
latcha_Myf5_53-156
homsap_Myf5_52-160
musmus_Myf5_52-160
galgal_Myf5_54-162
orenil_MyoD1_78-186
tetnig_MyoD1_78-186
danrer_MyoD_53-161
orenil_MyoD2_59-163
tetnig_MyoD2_64-172
lepocu_MyoD_53-161
salsal_MyoD1b_53-161
salsal_MyoD1c_53-161
xenlae_MyoDL_66-174
salsal_MyoD1a_53-161
salsal_MyoD1d_53-161
xentro_MyoD_64-172
xenlae_MyoDS_64-172
galgal_MyoD_70-178
homsap_MyoD_78-186
musmus_MyoD_78-186
calmil_MyoD_53-161
latcha_MyoD_54-162
rhityp_MyoD_53-161
0.3

c. NJ

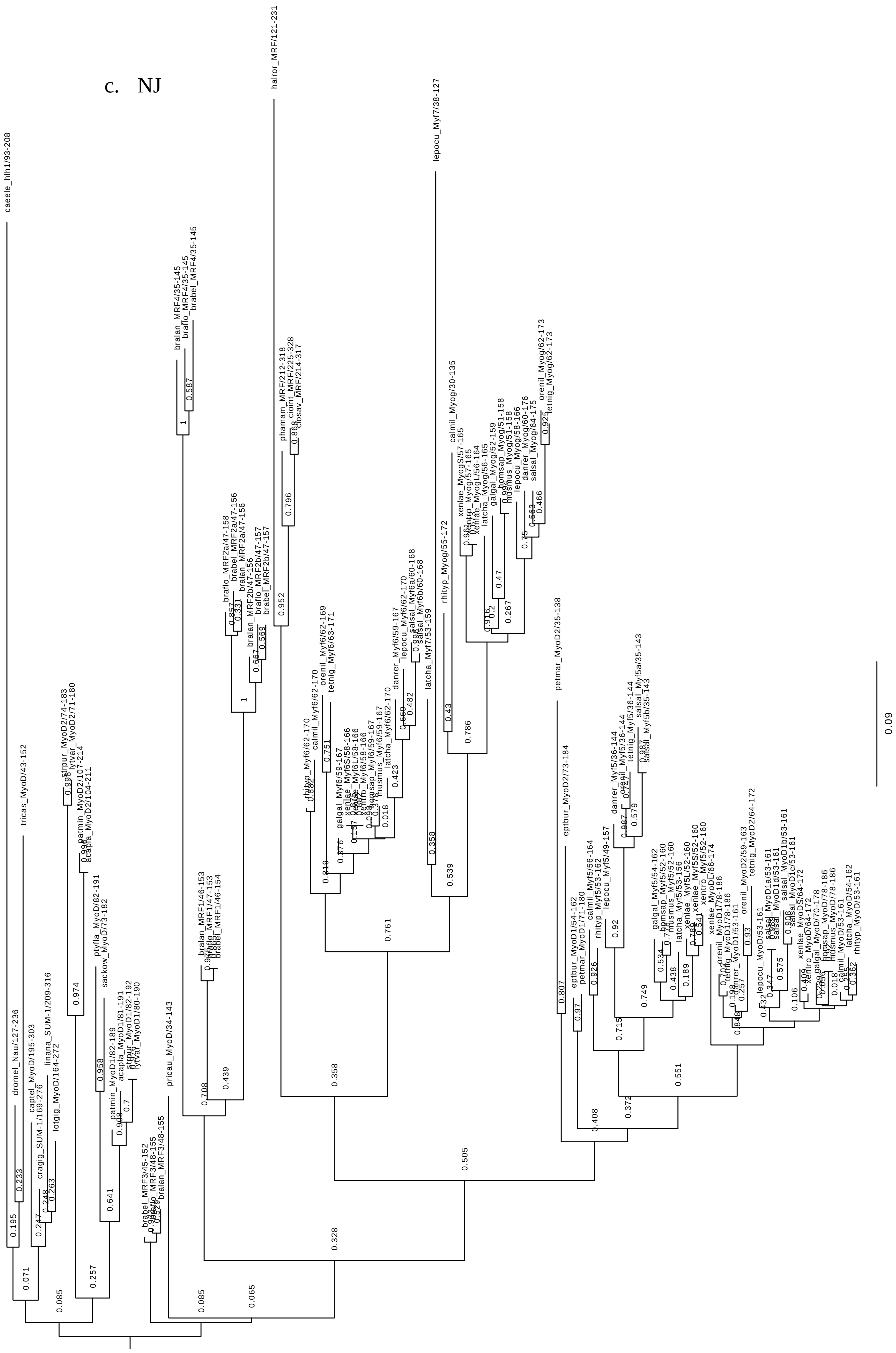

caeele_hlh1/93-208
halror_MRF/121-231
lepocu_Myf7/38-127
tricas_MyoD/43-152
dromel_Nau/127-236
captel_MyoD/195-303
cragig_SUM-1/169-276
linana_SUM-1/209-316
lotgig_MyoD/164-272
strpur_MyoD2/74-183
lytvar_MyoD2/71-180
patmin_MyoD2/107-214
acapla_MyoD2/104-211
ptyfla_MyoD/82-191
sackow_MyoD/73-182
patmin_MyoD1/82-189
acapla_MyoD1/81-191
lytvar_MyoD1/80-190
brabel_MRF3/45-152
bralan_MRF3/48-155
pricau_MyoD/34-143
bralan_MRF4/35-145
braflo_MRF4/35-145
brabel_MRF4/35-145
bralan_MRF1/46-153
braflo_MRF1/47-153
brabel_MRF1/46-154
braflo_MRF2a/47-158
brabel_MRF2a/47-156
bralan_MRF2a/47-156
bralan_MRF2b/47-156
braflo_MRF2b/47-157
brabel_MRF2b/47-157
phamam_MRF/212-318
cioint_MRF/225-328
ciosav_MRF/214-317
rhityp_Myf6/62-170
calmil_Myf6/62-170
orenil_Myf6/62-169
tetnig_Myf6/63-171
galgal_Myf6/59-167
homsap_Myf6/59-167
musmus_Myf6/59-167
latcha_Myf6/62-170
danrer_Myf6/59-167
lepocu_Myf6/62-170
salsal_Myf6a/60-168
salsal_Myf6b/60-168
latcha_Myf7/53-159
rhityp_Myog/55-172
calmil_Myog/30-135
xenlae_MyogS/57-165
galgal_Myog/52-159
homsap_Myog/51-158
musmus_Myog/51-158
lepocu_Myog/58-166
danrer_Myog/60-176
salsal_Myog/64-175
orenil_Myog/62-173
tetnig_Myog/62-173
petmar_MyoD2/35-138
eptbur_MyoD2/73-184
eptbur_MyoD1/54-162
petmar_MyoD1/71-180
calmil_Myf5/56-164
rhityp_Myf5/53-162
lepocu_Myf5/49-157
danrer_Myf5/36-144
orenil_Myf5/36-144
tetnig_Myf5/36-144
salsal_Myf5a/35-143
salsal_Myf5b/35-143
galgal_Myf5/54-162
homsap_Myf5/52-160
musmus_Myf5/52-160
latcha_Myf5/53-156
xenlae_Myf5L/52-160
xenlae_Myf5S/52-160
xentro_Myf5/52-160
xenlae_MyoDL/66-174
orenil_MyoD1/78-186
tetnig_MyoD1/78-186
danrer_MyoD/53-161
orenil_MyoD2/59-163
tetnig_MyoD2/64-172
lepocu_MyoD/53-161
salsal_MyoD1a/53-161
salsal_MyoD1d/53-161
salsal_MyoD1b/53-161
salsal_MyoD1c/53-161
xenlae_MyoDS/64-172
xentro_MyoD/64-172
galgal_MyoD/70-178
homsap_MyoD/78-186
musmus_MyoD/78-186
calmil_MyoD/53-161
latcha_MyoD/54-162
rhityp_MyoD/53-161
0.09

d. ML phylogeny without cyclostomes

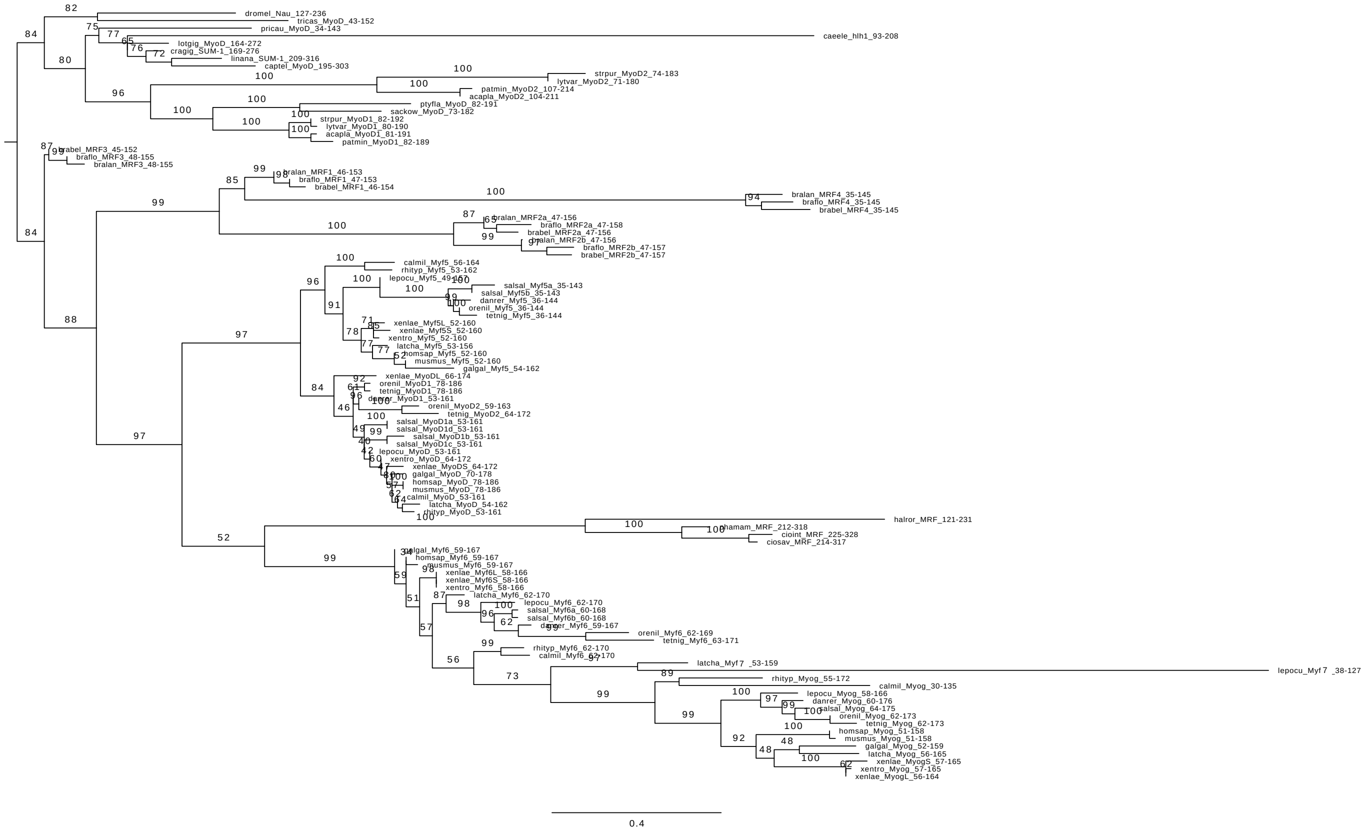

e. ML phylogeny with cyclostomes

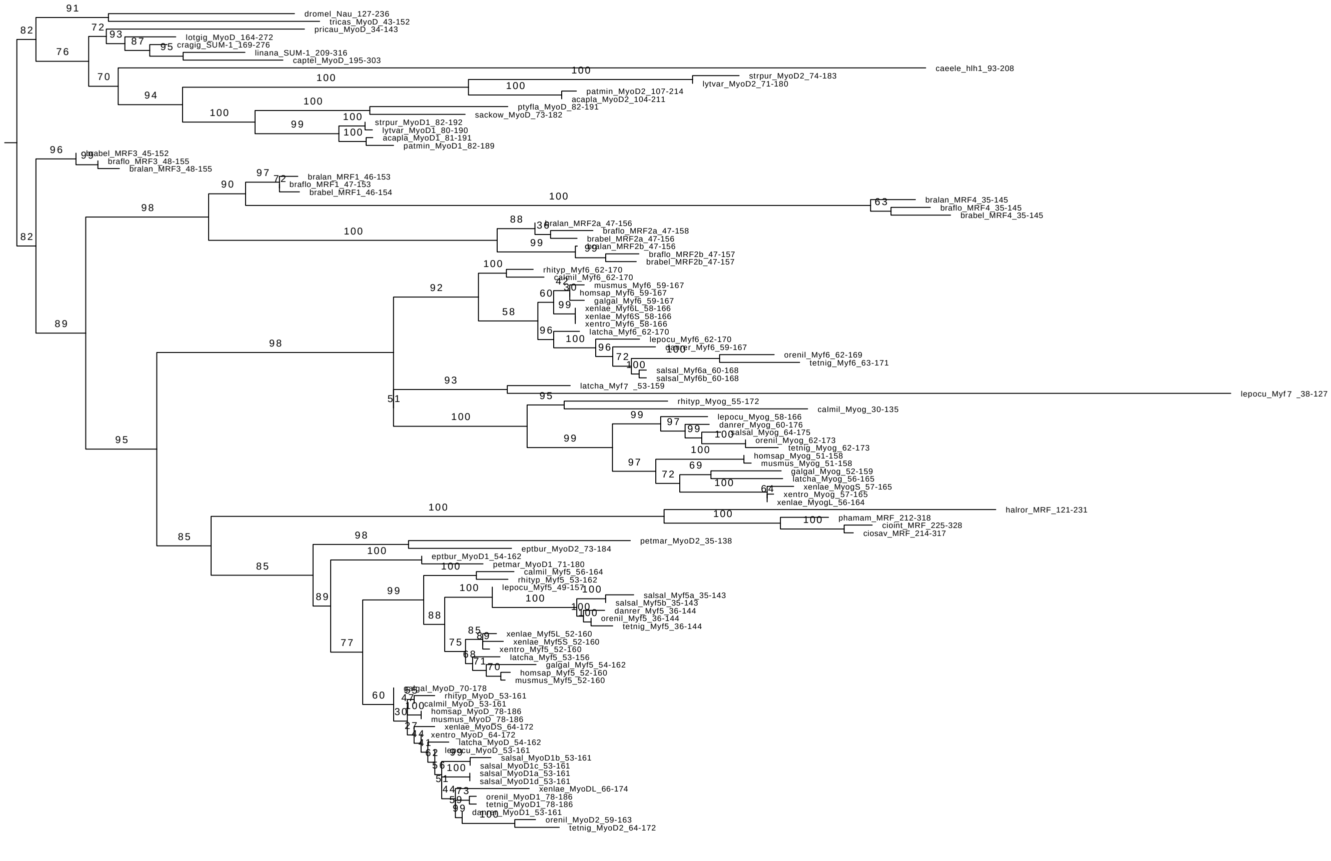

## 4. Alignments

### a. Full length *MRF* alignment

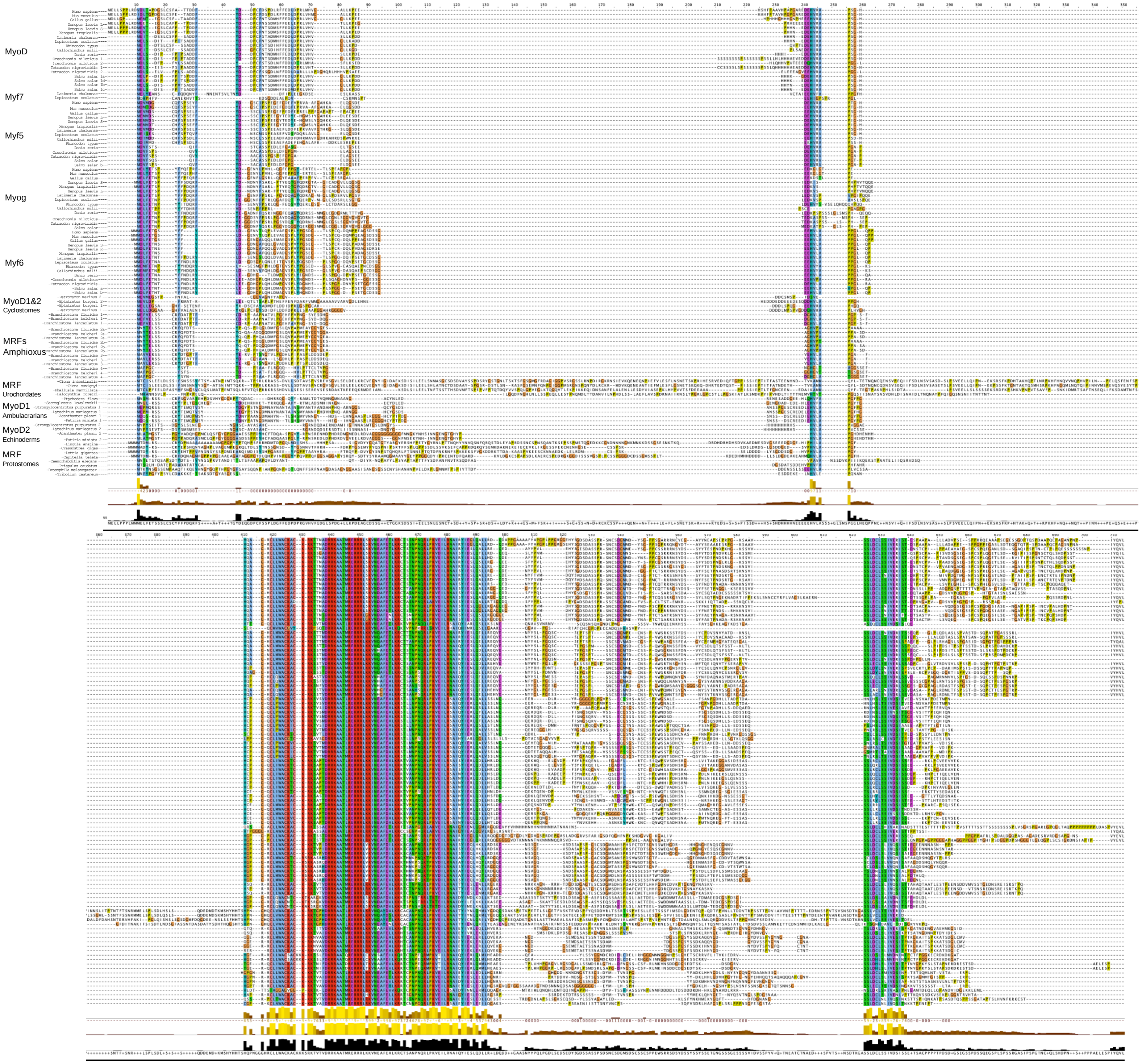

b. Trimmed *MRF* alignment

homsap_MyoD DEHVRA---------PSG-HHQA---G-RCLLWACKAC--K-RKTTNADRRKAATMRERRRLSKVNEAFETLKRCTSSNPNQRLPKVEILRNAIRYIEGLQALLRDSSLDCLSSIVERISTIYQVL-
musmus_MyoD DEHVRA---------PSG-HHQA---G-RCLLWACKAC--K-RKTTNADRRKAATMRERRRLSKVNEAFETLKRCTSSNPNQRLPKVEILRNAIRYIEGLQALLRDSSLDCLSSIVERISTIYQVL-
galgal_MyoD EEHVRA---------PSG-HHQA---G-RCLLWACKAC--K-RKTTNADRRKAATMRERRRLSKVNEAFETLKRCTSTNPNQRLPKVEILRNAIRYIESLQALLRESSLDCLSSIVERISTIYQVL-
xenlae_MyoDL EEHVRA---------PSG-HHQA---G-RCLLWACKAC--K-RKSSGADRRRAATMRERRRLSKVNDAFETLKRCTSTNPNQRLPKVDILRNAISYIDSLQTLLRDSSLDCLSSIVERISTIYQIL-
xenlae_MyoDS DEHVRA---------PSG-HHQA---G-RCLLWACKAC--K-RKTTNADRRKAATMRERRRLSKVNEAFETLKRYTSTNPNQRLPKVEILRNAIRYIESLQSLLHDSSLDCLSSIVERISTIYHVL-
xentro_MyoD DEHVRA---------PSG-HHQA---G-RCLLWACKAC--K-RKTTNADRRKAATMRERRRLSKVNEAFETLKRCTSTNPNQRLPKVEILRNAIRYIESLQSLLRGSSLDCLSSIVERISTIYQVL-
latcha_MyoD DEHIRV---------PSG-HYQA---G-RCLLWACKAC--K-RKTTNADRRKAATMRERRRLSKVNEAFETLKRCTSTNPNQRLPKVEILRNAIRYIESLQSLLRESSLDCLSSIVERISTIYQVL-
lepocu_MyoD DEHIRA---------PSG-HHQA---G-RCLLWACKAC--K-RKTTNADRRKAATMRERRRLSKVNEAFETLKRCTSTNPNQRLPKVEILRNAISYIESLQSLLRGSSLDCLSSIVERISTIYQVL-
rhityp_MyoD DEHIRA---------PSG-HHQA---G-RCLLWACKAC--K-RKTTNADRRKAATMRERRRLSKVNEAFETLKRCTSTNPNQRLPKVEILRNAIRYIESLQALLRESSLDCLTNIVERISTIYQVL-
calmil_MyoD DEHIRA---------PSG-HHQA---G-RCLLWACKAC--K-RKTTNADRRKAATMRERRRLSKVNEAFETLKRCTSTNPNQRLPKVEILRNAIRYIESLQALLRDSSLDCLSSIVERISTIYQVL-
danrer_MyoD DEHVRA---------PSG-HHQA---G-RCLLWACKAC--K-RKTTNADRRKAATMRERRRLSKVNDAFETLKRCTSTNPNQRLPKVEILRNAISYIESLQALLRSSSLDCLSSIVERISTIYQVL-
orenil_MyoD1 DEHVRA---------PSG-HHQA---G-RCLLWACKAC--K-RKTTNADRRKAATLRERRRLSKVNDAFETLKRCTTANPNQRLPKVEILRNAISYIESLQALLRGSSLDCLSSIVERISTIYQVL-
orenil_MyoD2 DQHVRA---------PGG-LHQA---G-HCLLWACKAC--K-RKTTHADRRKAATMRERRRLSKVNDAFETLKRCTASNPNQRLPKVEILRNAISYIESLQALLRNSSLDCLSSIVERISTI-----
tetnig_MyoD1 DEHVRA---------PSG-HHQA---G-RCLLWACKAC--K-RKTTNADRRKAATLRERRRLSKVNEAFETLKRCTSANPNQRLPKVEILRNAISYIESLQALLRGSSLDCLSSIVERISTIYQVL-
tetnig_MyoD2 EEHVRA---------PGG-LHQA---G-RCLLWACKAD--K-RKTTHADRRKAATMRERRRLSKVNDAFETLKRCTASNPNQRLAKVEILRNAISYIESLQALLRTSSLECLSSIVERISTIYEPL-
salsal_MyoD1a DEHIRA---------PSG-HHQA---G-RCLLWACKAC--K-RKTTNADRRKAATMRERRRLSKVNDAFETLKRCTSTNPNQRLPKVDILRNAISYIESLQGLLRGSSLDCLSNIVERITTIYQVL-
salsal_MyoD1d DEHIRA---------PSG-HHQA---G-RCLLWACKAC--K-RKTTNADRRKAATMRERRRLSKVNDAFETLKRCTSTNPNQRLPKVDILRNAISYIESLQGLLRGSSLDCLSNIVERITTIYQVL-
salsal_MyoD1b DEHIRA---------PSG-HHQA---G-RCLLWACKAC--K-RKTTNSDRRKAATMRERRRLGKVNDAFENLKRCTSNNPNQRLPKVEILRNAISYIESLQSLLRGSSLDCLSNIVERIFTIYQVL-
salsal_MyoD1c DEHIRA---------PSG-HHQA---G-RCLLWACKAC--K-RKTTNTDRRKAATMRERRRLGKVNDAFENLKRCTSNNPNQRLPKVEILRNAISYIESLQSLLRGSSLDCLSSIVERISTIYQVL-
homsap_Myf5 DEHVRA---------PTG-HHQA---G-HCLMWACKAC--K-RKSTTMDRRKAATMRERRRLKKVNQAFETLKRCTTTNPNQRLPKVEILRNAIRYIESLQELLRESSLDCLSNIVDRITSIYHVL-
musmus_Myf5 EEHVRA---------PTG-HHQA---G-HCLMWACKAC--K-RKSTTMDRRKAATMRERRRLKKVNQAFETLKRCTTTNPNQRLPKVEILRNAIRYIESLQELLRESSLDCLSSIVDRITSIYHVL-
galgal_Myf5 EEHVRA---------PSG-HHQA---G-HCLMWACKAC--K-RKSTTMDRRKAATMRERRRLKKVNQAFETLKRCTTANPNQRLPKVEILRNAIRYIESLQELLRESSLDCLSSIVDRLSPTYQAL-
xenlae_Myf5L DEHVRA---------PIG-HHQA---G-NCLMWACKAC--K-RKSSTTDRRKAATMRERRRLKKVNQAFETLKRCTTTNPNQRLPKVEILRNAIQYIESLQDLLRESSLDCLSSIVDRISSIYHVL-
xenlae_Myf5S DEHVRA---------PIG-HHQA---G-NCLMWACKAC--K-RKSSTMDRRKAATMRERRRLKKVNHAFETLKRCTSTNPNQRLPKVDILRNAIKYIESLQDLLRESSLDCLSSIVDRISSIYHVL-
xentro_Myf5 DEHVRA---------PIG-HHQA---G-NCLMWACKAC--K-RKSSTMDRRKAATMRERRRLKKVNQAFETLKRCTTTNPNQRLPKVEILRNAIKYIESLQDLLQESSLDCLSSIVDRISSIYHVL-
latcha_Myf7 EEHVFA---------PPGFHHTT---G-QCLLWACKIC--K-RKSVTMDRRKAATLRERRRLKRVNEAFETLKRKTVPNPNQQLPKVEILRSAIQYIARLQSLLKSASLQCLSSIVDSISLQE----
lepocu_Myf7 EEHIGPSPR------PGL--SER-----QCMVWACGVY--K-RRPSQTDRRRAATLRERKRLKRVNEAFEALKQKTVPNPKRRLSKVEILRHAIQHIMRLQSLLGA--------------------
latcha_Myf5 DEHIRA---------PSG-HHQA---G-HCLMWACKAC--K-RKSTTMDRRKAATMRERRRLKKVNQAFETLKRCTTSNPNQRLPKVEILRNAIKYIESLQDLLRESSLDCLSNIVDRISS------
lepocu_Myf5 DEHVRV---------PSG-HHQA---G-HCLMWACKAC--K-RKSSTVDRRKAATMRERRRLKKVNQAFETLRRCTSANPNQRLPKVEILRNAIQYIESLQDLLRESSLECLSSIVERLSSIYHVL-calmil_Myf5 EEHIRA---------PSD-HHQP---
G-HCLLWACKAC--K-RKSSTTDRRKAATMRERRRLKKVNQAFETLKRCTSSNPNQRLPKVEILRNAISYIENLQDVLRESSLDYLSNIVERISPVYHVL-
rhityp_Myf5 EKHVRA---------PSS-HHQAG--G-HCLLWACKAC--K-RKSSSTDRRKAATMRERRRLKKVNQAFETLKRCTSSNPSQRLPKVEILRNAISYIESLQELLRESSLDYLSNIVERISPVYHVL-
danrer_Myf5 DEHVRA---------PGA-PHQP---G-HCLQWACKAC--K-RKASTVDRRRAATMRERRRLKKVNHAFEALRRCTSANPSQRLPKVEILRNAIQYIESLQELLRESSLQCLSSIVDRLSSVYHVL-
orenil_Myf5 DEHVRV---------PGA-PHQP---G-HCLQWACKAC--K-RKSSFVDRRRAATMRERRRLKKVNHAFEALRRCTSANPSQRLPKVEILRNAIQYIESLQELLRESSLECLSSIVDRLSSVYHVL-
tetnig_Myf5 DEHVRV---------PGA-PHQP---G-HCLPWACKAC--K-RKSNFVDRRRAATMRERRRLKKVNHAFEALRRCTSANSSQRLPKVEILRNAIQYIESLQELLRESSLECLSSIVDRLSSVYHVL-
salsal_Myf5a DEHVRV---------PGT-PHQA---G-HCLQWACKAC--K-RKSSTVDRRRAATMRERRRLRKVNHGFEALRRCTSANHSQRLPKVEILRNAIQYIESLQELLHESSLARLSNIVDRLSSVYHVL-
salsal_Myf5b DEHVRV---------PGA-PHQA---G-HCLQWACKAC--K-RKSSTVDRRRAATMRERRRLKKVNHGFEALRRCTSANPSQRLPKVEILRNAIQYIESLQELLHESSLACLSNIVDRLSSVYHVL-
homsap_Myog EDKGLGT--------PE---HCP---G-QCLPWACKVC--K-RKSVSVDRRRAATLREKRRLKKVNEAFEALKRSTLLNPNQRLPKVEILRSAIQYIERLQALLSSHNLHSLTSIVDSITVETMPN-
musmus_Myog EEKGLGT--------PE---HCP---G-QCLPWACKVC--K-RKSVSVDRRRAATLREKRRLKKVNEAFEALKRSTLLNPNQRLPKVEILRSAIQYIERLQALLSSHNLHSLTSIVDSITVETMPN-
galgal_Myog EEKDSTL--------PE---HCP---G-QCLPWACKIC--K-RKTVSIDRRRAATLREKRRLKKVNEAFEALKRSTLLNPNQRLPKVEILRSAIQYIERLQSLLSSRNLHSLSSIVESIAVERVQN-
xenlae_MyogS EDKIS----------PHPNVHCP---G-QCLPWACKVC--K-RKTVSMDRRKAATLREKRRLKKVNEAFEALKRSTLLNPNQRLPKVEILRSAIQYIERLQTLLASRDINSLSSIVNSITSQHIQN-
xentro_Myog EDKVS----------PHPTVHCP---G-QCLPWACKVC--K-RKTVSMDRRRAATLREKRRLKKVNEAFEALKRSTLLNPNQRLPKVEILRSAIQYIERLQTLLASRDINSLSSIVDSITSQHIQH-
xenlae_MyogL EDKVS----------PHPTVHCP---G-QCLPWACKVC--K-RKTVSMDRRKAATLREKRRLKKVNEAFEALKRSTLLNPNQRLPKVEILRSAIQYIERLQTLLASRDINSLSSIVDSITSQHIQN-
latcha_Myog EDKVSP---------VPGIPHCP---G-QCLPWACKIC--K-RKTVSIDRRRAATLREKRRLKKVNEAFEALKRSTLLNPNQRLPKVEILRSAIQYIERLQSLLSTPSLHSLSSIVDSIAAGTILN-
lepocu_Myog EDKVSP---------AASLSHCP---G-QCLPWACKIC--K-RKSVTMDRRKAATLREKRRLKKVNEAFEALKRSTLMNPNQRLPKVEILRSAIQYIERLQALVSSPNLRSLSSIVDSITV-DISK-
rhityp_Myog DEHVYS-VSELQHQQQHPQQSCP---G-QCLLWACKSC--K-RKSVTLDRRKAATLREKRRLKKVNEAFEALKRSTLLNPNQRLPKVEILRSAIQYIERLQALLSTTSLCSLSSIVDSITPDNTPN-
calmil_Myog CE-------------PGAGPLPP---G-QCLLWACGTC--K-RKAVSGDRRRAATLREKRRLKKVNEAFEALKRSTLLNPSQRLPKVEILRSAIHYIARLQGLLSLTSLRSLCSIVDTITPESISN-
danrer_Myog EDKPSPSSSLGLSMSPH--QHCP---G-QCLPWACKVC--K-RKSVTMDRRKAATLREKRRLKKVNEAFEALKRSTLMNPNQRLPKVEILRSAIQYIERLQALVSSSNLRSLTSIVDSITGVDISK-
orenil_Myog EDKASPSS-----MSPH--SHCP---G-QCLPWACKLC--K-RKTVTMDRRRAATLREKRRLKKVNEAFDALKRSTLMNPNQRLPKVEILRSAIQYIERLQALVSSGNMRALTSIVDSISAVDIPK-

tetnig_Myog EDKASPSS---LS--PH--SHCP---G-QCLPWACKIC--K-RKTVTMDRRRAATLREKRRLKKVNEAFDALKRSTLMNPNQRLPKVEILRSAIQYIERLQALVSSGSMRTLTAIVDSISAMDIPK-
salsal_Myog EDKATPS---GLS--PH--PHCP---G-QCLPWACKLC--K-RKTVTMDRRKAATMREKRRLKKVNEAFEALKRSTLMNPNQRLPKVEILRSAIQYIERLQALVSSTNLRSLTSIVDSITAVDIPK-
homsap_Myf6 EEHVLA---------PPGL-HCP---G-QCLIWACKTC--K-RKSAPTDRRKAATLRERRRLKKINEAFEALKRRTVANPNQRLPKVEILRSAISYIERLQDLLHRSSLRCLSSIVDSISSEVVEK-
musmus_Myf6 EEHVLA---------PPGL-HCP---G-QCLIWACKTC--K-RKSAPTDRRKAATLRERRRLKKINEAFEALKRRTVANPNQRLPKVEILRSAISYIERLQDLLHRSSLQRLSSIVDSISSEVVEK-
galgal_Myf6 EEHVLA---------PPGL-HCP---G-QCLIWACKTC--K-RKSAPTDRRKAATLRERRRLKKVNEAFEALKRRTVANPNQRLPKVEILRSAISYIERLQDLLHRSSLRCLSSIVDSISSEAVEK-
xenlae_Myf6S EEHVLA---------PPGL-HCP---G-QCLIWACKTC--K-RKSAPTDRRKAATLRERRRLKKINEAFEALKRRTVANPNQRLPKVEILRSAINYIERLQDLLHSSSLQCLSSIVDSISSELVEN-
xenlae_Myf6L EEHVLA---------PPGL-HCP---G-QCLIWACKTC--K-RKSAPTDRRKAATLRERRRLKKINEAFEALKRRTVANPNQRLPKVEILRSAINYIERLQDLLHSSSLQCLSSIVDSISSELVEN-
xentro_Myf6 EEHVLA---------PPGL-HCP---G-QCLIWACKTC--K-RKSAPTDRRKAATLRERRRLKKINEAFEALKRRTVANPNQRLPKVEILRSAINYIERLQDLLHSSSLQCLSSIVDSISSELVEN-
latcha_Myf6 EEHVLA---------PPGL-HCP---G-QCLIWACKTC--K-RKSAPTDRRKAATLRERRRLKKINEAFEALKRRTVPNPNQRLPKVEILRSAINYIEKLQDLLHTTSLRCLSSIVDSISSEGEEN-
lepocu_Myf6 EEHVLA---------PPGL-HCE---G-QCLIWACKTC--K-RKSAPTDRRKAATLRERRRLKKINEAFEALKKKTVPNPSQRLPKVEILRSAIKYIEKLQDILHNTSLRRLSSIVDSISSDASEK-
rhityp_Myf6 EEHVYA---------PPGL-HCP---G-QCLIWACKAC--K-RKSASTDRRKAATLRERRRLKKINEAFEALKRRTVPNPNQRLPKVEILRSAIHYIEKLQDLLHSSSLHYLSSIVDSISTDSNAK-
calmil_Myf6 EEHVYA---------PPGL-HCP---G-QCLIWACKAC--K-RKSATTDRRKAATLRERRRLKKINEAFDALKRRTVPNPNQRLPKVEILRSAIHYIEKLQDLLHSSSLRYLTSIVDSISTSTITK-
danrer_Myf6 EEHVLA---------PPGL-HCE---G-QCLMWACKIC--K-RKSAPTDRRKAATLRERRRLKKINEAFDALKKKTVPNPNQRLPKVEILRSAINYIEKLQDLLHSSSLRRLSSIVDSISTQISEK-
orenil_Myf6 EEHVPA---------PPGL-HCE---G-QCLMWACKIC--K-RKTAPTDRRKAATLRERRRLKKINEAFDALKRKTVANPNQRLPKVEILRSAISYIERLQELLQTSSLLRLSSIVDSITNDSSH--
tetnig_Myf6 DEHVLA---------PPGL-HCE---G-QCLMWACKVC--K-RKSAPTDRRKAATLRERRRLKKINEAFDALKRKSVANPNQRLPKVEILRSAISYIERLQELLQSSSLLCLSSIVSSISDSVPGN-
salsal_Myf6a EEHVLA---------HPGL-HCE---G-QCLIWACKVC--K-RKSAPTDRRKAATLRERRRLKKINEAFDALKKKTVPNPNQRLPKVEILRSAINYIEQLQDLLHTTSLLRLSSIVDSISSEVSEK-
salsal_Myf6b EEHVLA---------PPGL-HCE---G-QCLIWACKVC--K-RKSAPTDRRKAATLRERRRLKRINEAFDALKKKTVPNPNQRLPKVEILRSAINYIEQLQDLLHTTSLLRLSSIVDSISSEVSEK-
petmar_MyoD2 FDSVE--------------QRS---G-ACLLWACRAC--K-RKTSSAERRRAATVRERRRLRRVNEAFETLKRFTSANPGQRLAKVEILRNAIGYIENLQGMLRGSSLDCLSHIVARINAVYEVL-
eptbur_MyoD2 DDHVLA---------PPGH-HTPGGGG-RCLAWACKAC--K-RKASSAERRRAATLRERRRLRRVNEAFEALKRCCSGNPHQRLAKVEILRNAIGYIEALQGVLRGSSLDCLSSIVERISTIYQAL-
eptbur_MyoD1 EEHVRA---------PHGQ-HGP---G-PCLLWACKAC--K-RKTSSTDRRKAATMRERRRLRKVNEAFETLKRCTSANPSQRLPKVEILRNAIRYIEGLQRLLRQSSLDCLSSIVERISSVYEVL-
petmar_MyoD1 DQHVRA---------PLHVGHGP---G-PCLLWACKAC--K-RKTSTTDRRKAATMRERRRLRKVNEAFETLKRCTSSNPSQRLPKVEILRNAIRYIEGLQRLLRQSSLDCLSSIVERISSVYEVL-
braflo_MRF1 DGHVLA---------PGP-SHGP---G-RCLLWACKAC--K-KKTVPIDRRKAATMRERRRLVKVNEAFDILKKKSCANPNQRLPKVEILRNAISYIEQLHKLLRDSSLDCLSLIVQSIST--PPR-
brabel_MRF1 DGHVLA---------PGP-SHGP---G-RCLLWACKAC--K-KKTVPIDRRKAATMRERRRLVKVNEAFDILKKKSCANPNQRLPKVEILRNAISYIEQLHKLLRDSSLDCLSLIVQSISTSNTAR-
bralan_MRF1 DGHVLA---------PGP-SHGP---G-RCLLWACKAC--K-KKTVPVDRRKAATMRERRRLVKVNEAFDILKKKSCANPNQRLPKVEILRNAISYIEQLHKLLRDSSLDCLSLIVQSISAN-PPR-
braflo_MRF2a AGHVPA---------PAAAAHGP---G-RCLMWACKTC--RSRKASRHDRRKAATMRERRRLVKVNEAFEVLKKKTHMKPNQKTPKVDILRNAIAYIEQLHQTLRDSCLDSLSLVVQNISAYTPLRS
brabel_MRF2a AGHVPV---------PPA-SHGP---G-RCLMWACKTC--RSRKASRQDRRKAATMRERRRLVKVNEAFEVLKRKTHMKPSQKTPKVDILRNAIAYIEQLHQTLRDSCLDSLSLVVQNISAYNQQR-
bralan_MRF2a AGHVPA---------PPA-SHGP---G-RCLMWACKTC--RSRKASRHDRRKAATMRERRRLVKVNEAFEVLKKKTHMKPSQKTPKVDILRNAISYIEQLHQTLRGSCLDSLSLVVQNISAYSQQR-
braflo_MRF2b AGHVPT---------PAAAAHGP---G-RCLMWACKTC--RSRKASRHDRRKAATMRERRRLVKVNEAFEVLKKKTHMKPNQKTPKVDILRNAIAYIEQLHQTLRDTSLDNLSSIVNSIALPDGHM-
brabel_MRF2b AGHVPV---------PPA-SHGP---G-RCLMWACKTC--RSRKASRQDRRKAATMRERRRLVKVNEAFEVLKRKTHMKPSQKTPKVDILRNAIAYIEQLHQTLRDTSLDNLSSIVNSIALPDTHLQ
bralan_MRF2b AGHVPA---------PPA-SHGP---G-RCLMWACKTC--RSRKASRHDRRKAATMRERRRLVKVNEAFEVLKKKTHMKPSQKTPKVDILRNAISYIEQLHQTLRGSSLDNLSSIVNSIALPDGNL-
braflo_MRF3 VEHVLA---------PGA--HSQ---R-RCLLWACKAC--K-RKSVTVDRRKAATMRERRRLRKVNEAFEVLKRRTCTNPNQRLPKVEILRNAITYIESLENLLRGSSLDCLSLIVQSISTRSTPQ-
brabel_MRF3 VEHVLA---------PGA--HSP---R-RCLLWACKAC--K-RKSVTVDRRKAATMRERRRLRKVNEAFEVLKRRTCTNPNQRLPKVEILRNAITYIESLENLLRGSSLDCLSLIVQSISTRTKPQ-
bralan_MRF3 VEHVLA---------PGA--HSQ---R-RCLLWACKAC--K-RKSVTVDRRKAATMRERRRLRKVNEAFEVLKRRTCTNPNQRLPKVEILRNAITYIESLENMLRGSSLDCLSLIVQSISSRNTPQ-
braflo_MRF4 NGHGLT---------AGGSSHGP---G-RCLQWACNTC--KTKARGKVDKRKAATMRERRRLSKVNDAFDVLKKKTSPNSTRRLTKTEILKNAIDYIMDLKDLLKTSSLDSLSAIVDNITSHDLDL-
brabel_MRF4 GGHGVT---------TGSGSHGP---G-RCLQWACNTC--KTKSRGKVDKRKAATMRERRRLSKVNDAFDVLKKKTSPNSTRRLTKTEILRNAIDYILELKKLLKASSLGSLSAIVDNITSHDLDL-
bralan_MRF4 DGHGLA---------TGGGSHGP---G-RCLQWACNTC--KAKSRGKVDKRKAATLRERRRLSRVNDAFDVLKKKTSPNSAQRLTKTDILKNAIDYIMELKNLLKTSSLDSLSAIVDNITSHELDL-
cioint_MRF TVKAMM---------QYL-THPN---GHQCLVWACKAC--K-RKTGPHDRRRAATLRERRRLKRVNQAYDALKRCACANPNQRLPKVEILRNAITYIYNLQHMLYGSSLVCLTSIVERID-------
ciosav_MRF TVKAMM---------QYL-SHPN---GHQCLVWACKAC--K-RKTGPHDRRRAATLRERRRLKRVNQAYDSLKRCACANPNQRLPKVEILRNAITYIYNLQRMLYGSSLVCLSSIVERID-------
phamam_MRF AIKAMM---------QYF-THPN---GHQCLAWACKAC--K-RKSGPHDRRRAATLRERRRLKRVNQAYETLKRCACANPNQRLPKVEILRNAITYICNLQRILYGSSLVCLSSIVESITDEL----
halror_MRF MVHTVT---------PGQSIHPN---GHQCLVWACKAC--K-RKTGPHDRRRAATLRERRRLKRVNEAYENLKRCACSNPNQRLPKVEILRNAITYIENLQRILYEKTSGNHDAVLITDIRYVKPQ-
ptyfla_MyoD LQHVFA---------PGFPGQTQ---R-KCLLWACKAC--K-KKTITVDKRKAATMRERRRLKKVNAAFEVLKRHTTSNPNQRLPKVEILRNAIEYIVRLEQLLHVTSLDRLSLIVERISPKGSID-
sackow_MyoD LEHVFA---------PGYTGQSQ---R-KCLLWACKAC--K-KKTVAIDKRKAATMRERRRLKKVNEAFELLKRHTSANPNQRLPKVEILRNAIEYIVRLEKLLHVSSLDSLSLIVESIKPYIDES-
strpur_MyoD1 LEHVLA---------PGFPGQGE---R-RCLMWACKAC--K-RKNVAVDKRKAATLRERRRLRKVNEAFEALKRHTCANPNQRLPKVEILRNAIEYIEKLERLLQVSSLDCLSLIVESITPDGLCMV
lytvar_MyoD1 LEHVLA---------PGFHGQGE---R-RCLMWACKAC--K-RKNVAVDKRKAATLRERRRLRKVNEAFEALKRHTCANPNQRLPKVEILRNAIEYIEKLERLLQVSSLDCLSLIVESITPDGLCMV
acapla_MyoD1 IEHVLA---------PGYPGGGE---R-RCLLWACKAC--K-RKNVAVDKRKAATMRERRRLRKVNEAFEALKRHTSANPNQRLPKVEILRNAIEYIEKLERLLQVSSLDCLSLIVESITPDGLCMC
patmin_MyoD1 IEHVLA---------PGYPGRER-----RCLLWACKAC--K-RKNVAVDKRKAATMRERRRLRKVNEAFEALKRHTSANPNQRLPKVEILRNAIEYIEKLERLLQVSSLDCLSLIVESITPDGIC--
strpur_MyoD2 --HVLA---------PVGGAHGE---R-RCLLWACSACKKK-KKSSLFDKRRAATQRERRRLCKVNSAFEILKQRTCSNPEQRMPKVTILRNAIQYIERLQLMLHESSLDSLSLMVDNINTDKGAT-
lytvar_MyoD2 --HVLA---------PVCSSHGE---R-RCLLWACSACKKK-KKSSLFDKRRAATQRERRRLCKVNAAFEILKQRTCSNPEQRMPKVTILRNAIQYIERLQLMLHESSLDHLSLMVENINTDKGAT-
patmin_MyoD2 --HVLV---------PGHDHTGE---R-QCLIWACKAC--K-RKTNIFDKRRAATDRERRRLTKVNAAFEVLKRRTCTNPEQRMPKVTILRNAIQYIERLQMMLHESSLDHLSLIVESITPELESP-
acapla_MyoD2 --HVLA---------PGHEHTGE---R-QCLIWACKAC--K-RKTNIFDKRRAATDRERRRLTKVNAAFEVLKRRTCTNPEQRMPKVTILRNAIQYIERLQMMLHESSLDHLSLIVESITPELESP-
linana_SUM-1 IPHILA---------PGF--GPGN--R-RCLLWACKAC--K-KKTVTIDRRKAATLRERRRLRKVNEAFETLKRRTCPNPNQRLPKVEILRNAIEYIESLEDLLHGSSLDCLSMIVDSINP-ERPL-

cragig_SUM-1  VPHVLA---------PGF--HGPN--R-RCLLWACKAC--K-RKTVAIDRRKAATLRERRRLRKVNEAFETLKRRTCPNPNQRLPKVEILRNAIEYIESLEELLHGSSLDCLSLIVESISP-ERPL-
lotgig_MyoD   VPHVLA---------PGY--HGPN--R-RCLLWACKAC--K-KKTVTIDRRKAATMRERRRLRRVNEAFEVLKRRTCPNPNQRLPKVEILRNAIDYIESLEDLLHGSSLDCLSLIVESIAPTDRPL-
captel_MyoD   MQHVLA---------PGFH-HGPT--R-RCLLWACKAC--K-KKTVAVDRRKAATMRERRRLRKVNEAFEHLKRRTCPNPNQRLPKVEILRNAIEYIESLEDLLHGSSLECLTSIVDSING-ERPL-
caeele_hlh1   -VPAVA---NSSDVKPIIIKHEDT--TTSTAGGAGVGG--P-RRTK-LDRRKAATMRERRRLRKVNEAFEVVKQRTCPNPNQRLPKVEILRSAIDYINNLERMLQQNSLDRLSRIVASIPNKLEML-
pricau_MyoD   VPHVFA---------PHFAHHAP---R-RCLLWACKAC--K-RKTVTVDRRKAATMRERRRLHKVNEAFELLKRRTSGNPNQRLPKVEILRNAIGYIESLEQLLRGSSLDCLSLIVENLVPRVHTA-
dromel_Nau    EEHVLA---------PLVCSQSS---R-PCLTWACKAC--K-KKSVTVDRRKAATMRERRRLRKVNEAFEILKRRTSSNPNQRLPKVEILRNAIEYIESLEDLLQESSLDCLNLIVQSINKRKCST-
tricas_MyoD   LNHVLE---------PQNANCGP---R-KCLAWACKAC--K-KKTVAIDRRKAATLRERRRLRKVNEAFEVLKRRTCNNPGQRLPKVEILRSAIEYIEYLEEILQGSSLDCLNLIVQSITNENEPT-
strpur_MyoD3  ---------------------------------------------RQRRVRQNATARERRRLAEVNRAYSNLSNHVPQHLLGNATKLEVLRGAACYIELLTILLSATQVKVADAVLSVDAAMRIRL-

5. Synteny of vertebrate *MRF* and ghost loci

a. *MyoD*

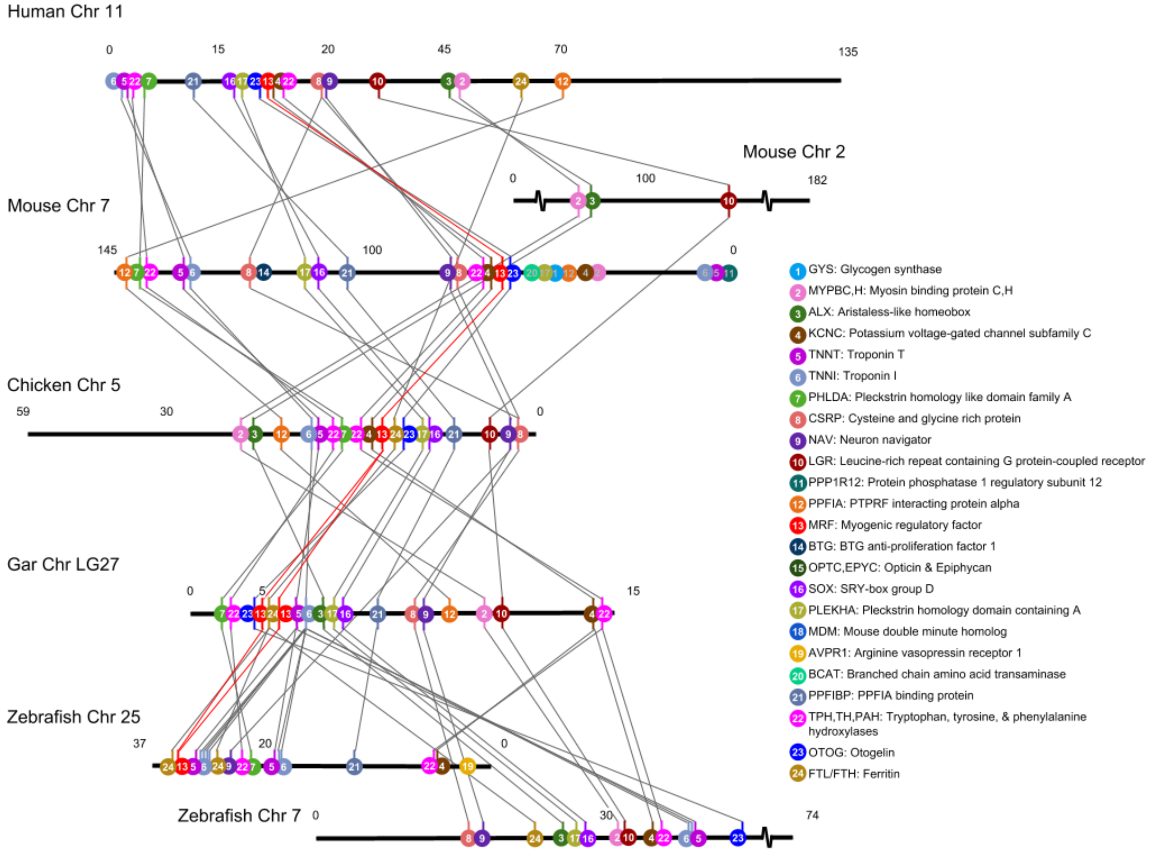


b. *Myog*

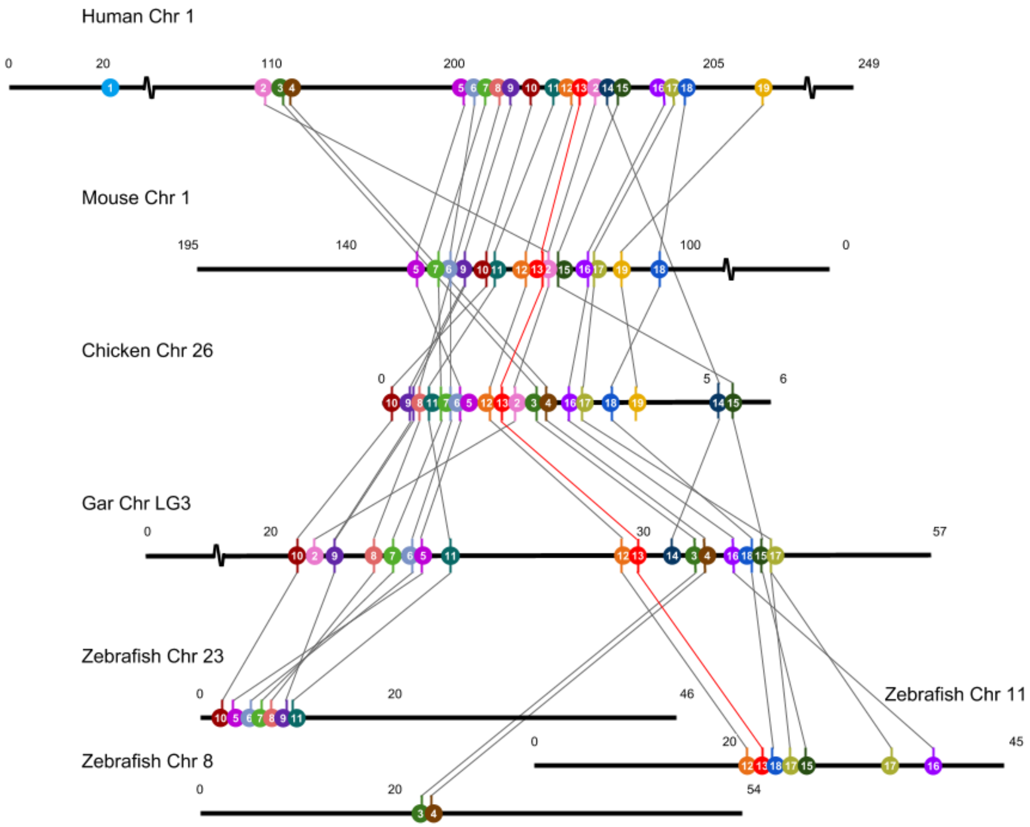

c. *Myf5-Myf6*

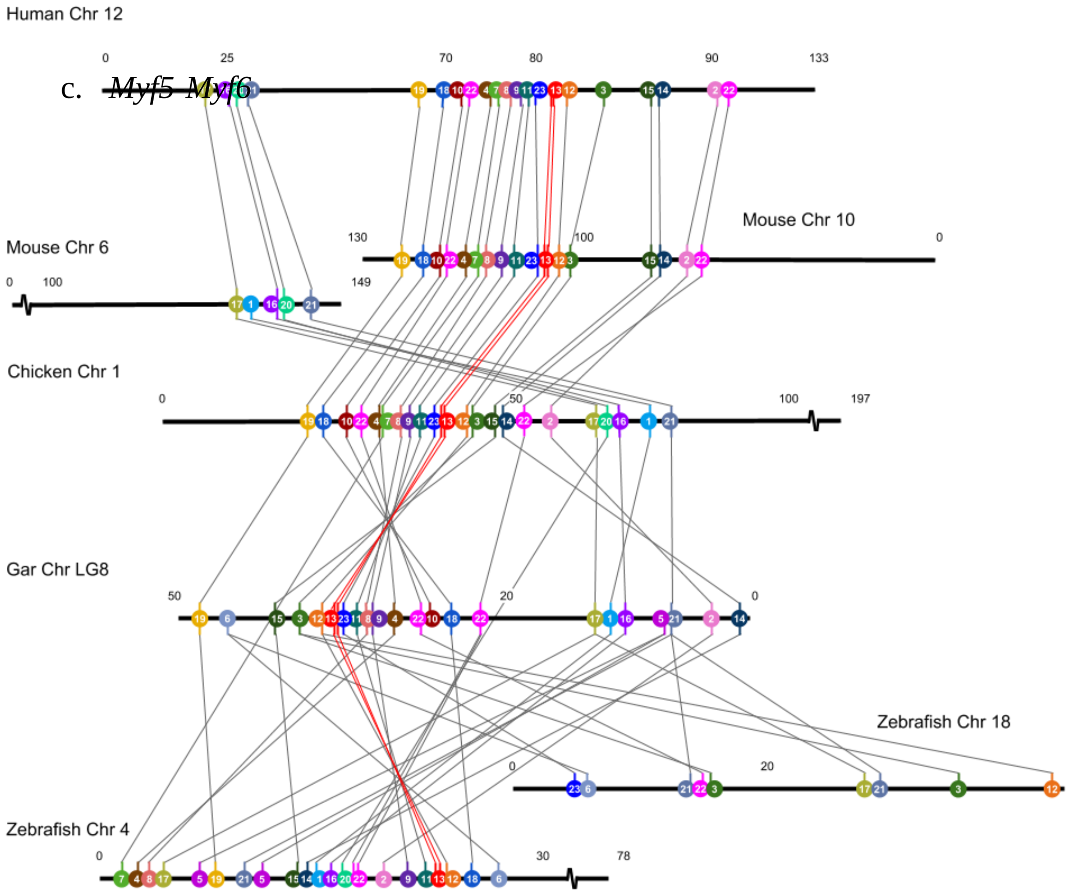


d. ghost *MRF*

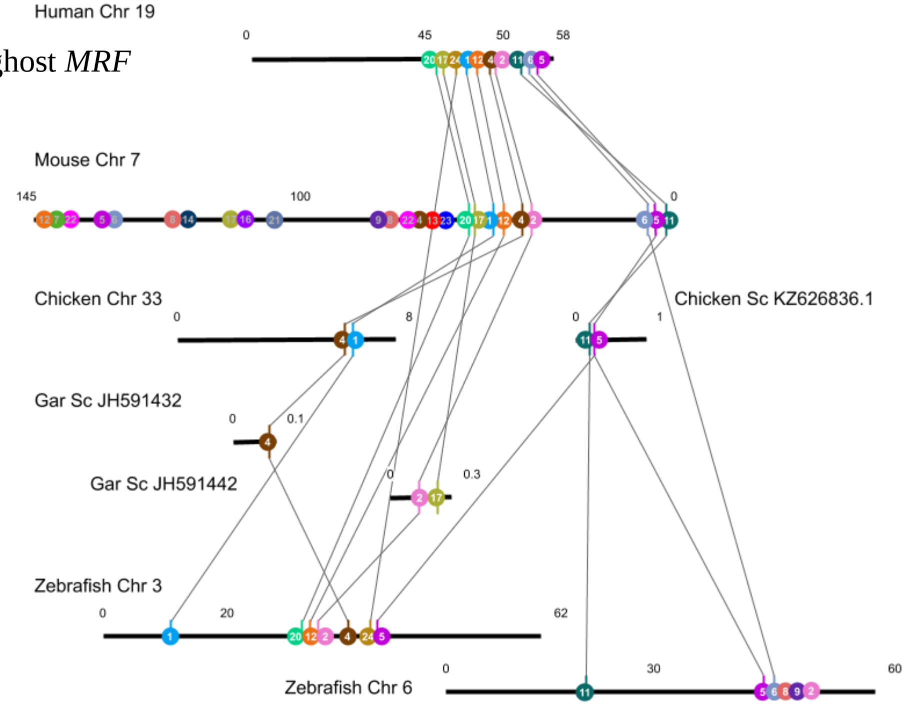

6. Synteny between human and amphioxus

a. Human *MRF*-linked orthologues of amphioxus *MRF*-linked genes peaks at the *MRF* locus

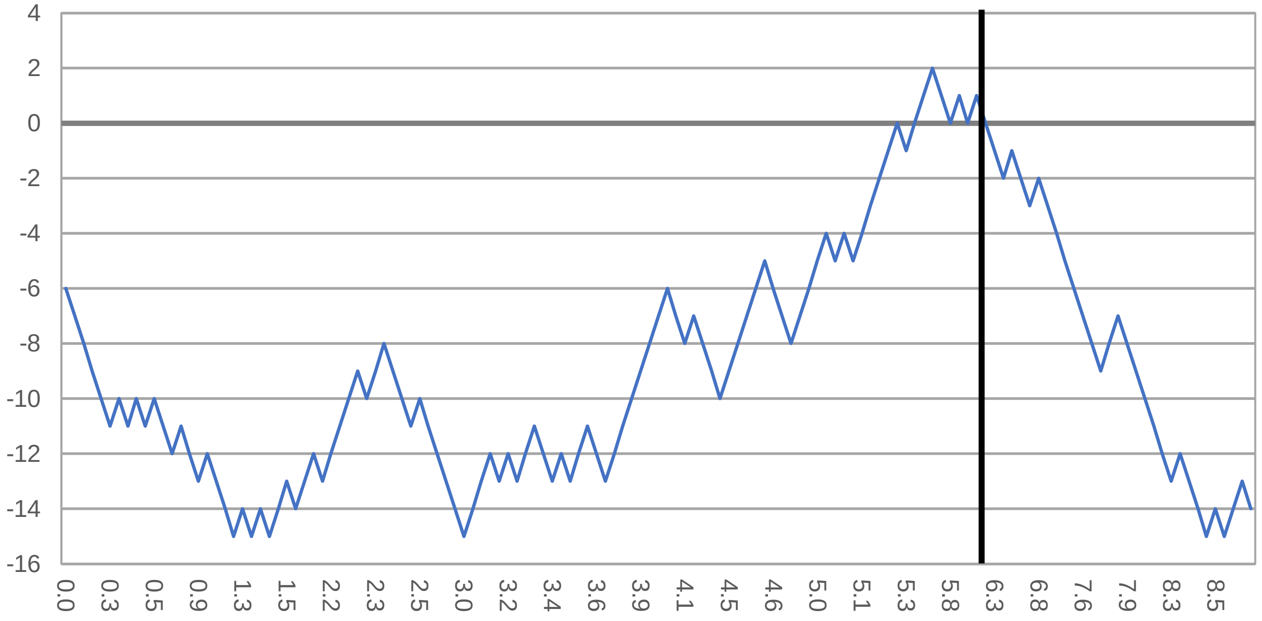


Starting from the MRF locus (~6.2 Mb, black line, 0), the number of human orthologues of *B. lanceolatum* MRF-linked genes that are linked to human MRF loci were added, while the number of genes elsewhere in the human genome were subtracted. The signal of synteny decreases with distance from the MRF locus.

b. Chromosome locations of 1:1 orthologues of amphioxus *MRF*-linked genes

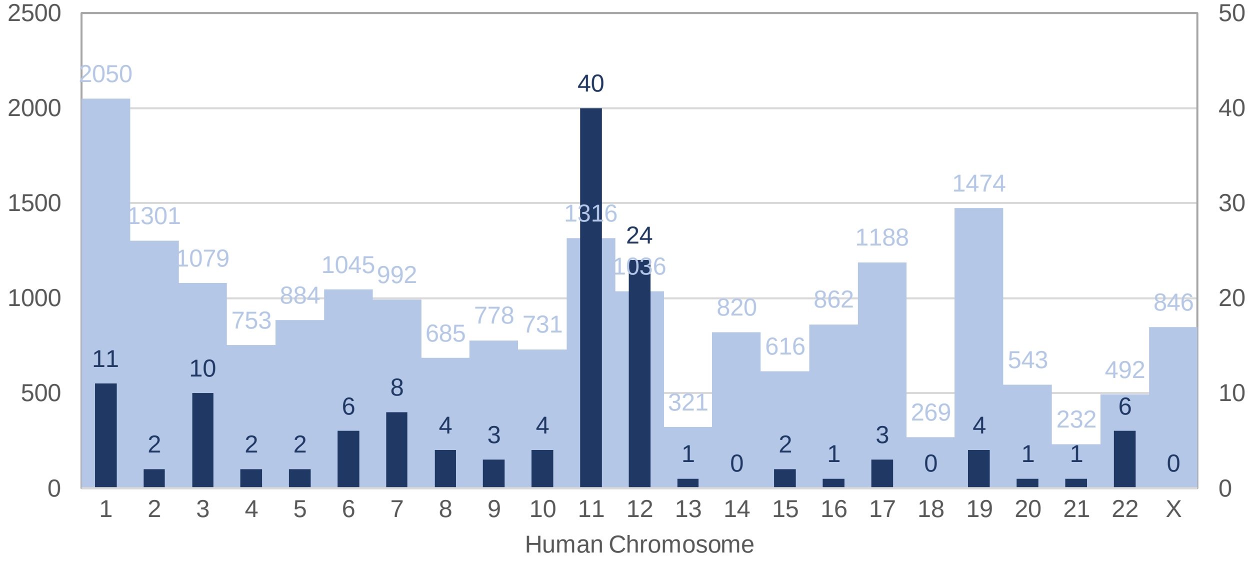


The total number of PCGs on each human chromosome (pale blue) and the number of genes on that chromosome with 1:1 orthology to the *B. lanceolatum* MRF-bearing scaffold #3 (dark blue).

c. Barnard's test

Is there a higher than expected proportion of orthologues of human genes linked to human MRF loci also linked to the amphioxus MRF locus?

This tests probability without replacement based on a contingency table. There are 135 human PCGs with 1:1 orthology to a gene on *B. lanceolatum* scaffold #3. Of those, 79 are linked to a human MRF locus, 56 are elsewhere in the genome. Of the genes without 1:1 orthology to a gene on *B. lanceolatum* scaffold #3 (20,174), 5,793 are linked to a human MRF locus (5,876-4; # on chromosomes 1, 11, 12, and 19 minus four MRFs) while 14,437 are elsewhere in the genome.

| **Observed** | Human MRF-linked genes | Human non-MRF-linked genes | Row total |
|---|---|---|---|
| With orthology | 79 | 56 | 135 |
| Without orthology | 5793 | 14381 | 20174 |
| Column total | 5872 | 14437 | 20309 |

| **Expected** | Human MRF-linked genes | Human non-MRF-linked genes | Row total |
|---|---|---|---|
| With orthology | 39.03 | 95.97 | 135 |
| Without orthology | 5832.97 | 14341.03 | 20174 |
| Column total | 5872 | 14437 | 20309 |

| **Chi-square** | | | |
|---|---|---|---|
| **(Observed-Expected)^2 / Expected** | Human MRF-linked genes | Human non-MRF-linked genes | |
| With orthology | 40.92 | 16.64 | |
| Without orthology | 0.27 | 0.11 | |
| Sum total | 57.95 | | |
| Pearson X2 | **57.954** | | |
| df | 1 | | |
| critical value (a=0.05) | 3.841 | critical value (a=0.01) | 6.635 |
| p | **(57.954 > critical value)** | **p < 0.05** | **p < 0.01** |
| **Fisher's exact test** | | | |
| One-tailed | **p=6.511E-13** | **p < 0.05** | **p < 0.01** |
| **Barnard's test** | | | |
| Nuisance parameter | 0.0001 | | |
| One-tailed | **p=5.435E-03** | **p < 0.05** | **p < 0.01** |
| Two-tailed | **p=1.087E-02** | **p < 0.05** | *p > 0.01* |

| **CHROMOSOME 1** | | | | |
|---|---|---|---|---|
| | On Chr1 | Elsewhere | | |
| Orthos | 11 | 124 | 135 | |
| Others | 2039 | 18139 | 20178 | |
| | 2050 | 18263 | 20313 | |
| Barnard's | Nuisance | 0.9898 | | |
| | One-tailed p | 0.25059 | *p > 0.05* | *p > 0.01* |
| | Two-tailed p | 0.50118 | *p > 0.05* | *p > 0.01* |

| **CHROMOSOME 12** | | | | |
|---|---|---|---|---|
| | On Chr12 | Elsewhere | | |
| Orthos | 24 | 111 | 135 | |
| Others | 1012 | 19166 | 20178 | |
| | 1036 | 19277 | 20313 | |
| Barnard's | Nuisance | 0.0001 | | |
| | One-tailed p | 0.009039 | **p < 0.05** | **p < 0.01** |
| | Two-tailed p | 0.018078 | **p < 0.05** | *p > 0.01* |

| **CHROMOSOME 11** | | | | |
|---|---|---|---|---|
| | On Chr11 | Elsewhere | | |
| Orthos | 40 | 95 | 135 | |
| Others | 1276 | 18902 | 20178 | |
| | 1316 | 18997 | 20313 | |
| Barnard's | Nuisance | 0.0001 | | |
| | One-tailed p | 0.0018311 | **p < 0.01** | **p < 0.01** |
| | Two-tailed p | 0.0036622 | **p < 0.01** | **p < 0.01** |

| **CHROMOSOME 19** | | | | |
|---|---|---|---|---|
| | On Chr19 | Elsewhere | | |
| Orthos | 4 | 131 | 135 | |
| Others | 1470 | 18708 | 20178 | |
| | 1474 | 18839 | 20313 | |
| Barnard's | Nuisance | 0.010199 | | |
| | One-tailed p | 0.049481 | **p < 0.05** | *p > 0.01* |
| | Two-tailed p | 0.098962 | *p > 0.05* | *p > 0.01* |

d. Exact binomial test

Is the proportion of human orthologues of MRF-linked amphioxus genes distributed among human MRF-linked and other chromosomes? This tests probability with replacement, based on two frequencies and the number of 'successes' or the number of human genes with 1:1 orthology to genes on *B. lanceolatum* scaffold #3.

The probability of a gene being on a certain chromosome in the genome (the number of genes on that chromosome divided by the number of genes in the genome) is compared to the probability of human genes with 1:1 orthology to genes on *B. lanceolatum* scaffold #3 being on a certain chromosome (the number of human orthologues of *B. lanceolatum* scaffold #3 genes on that chromosome divided by the total number of human orthologues of *B. lanceolatum* scaffold #3 genes). The MRF row combines all MRF-linked chromosomes (1, 11, 12, and 19).

| Chr | Genes | Probability | 1:1 Orthos | P fewer 1:1 Orthos | P more 1:1 Orthos | a=0.05 | a=0.01 |
|---|---|---|---|---|---|---|---|
| 1 | 2050 | 0.10092 | 11 | 0.2802 | 0.7198 | *p > a* | *p > a* |
| 2 | 1301 | 0.06405 | 2 | 0.0069 | 0.9931 | *p > a* | *p > a* |
| 3 | 1079 | 0.05312 | 10 | 0.8948 | 0.1052 | *p > a* | *p > a* |
| 4 | 753 | 0.03707 | 2 | 0.1196 | 0.8804 | *p > a* | *p > a* |
| 5 | 884 | 0.04352 | 2 | 0.0637 | 0.9363 | *p > a* | *p > a* |
| 6 | 1045 | 0.05144 | 6 | 0.4542 | 0.5458 | *p > a* | *p > a* |
| 7 | 992 | 0.04884 | 8 | 0.7848 | 0.2152 | *p > a* | *p > a* |
| 8 | 685 | 0.03372 | 4 | 0.5204 | 0.4796 | *p > a* | *p > a* |
| 9 | 778 | 0.03830 | 3 | 0.2364 | 0.7636 | *p > a* | *p > a* |
| 10 | 731 | 0.03599 | 4 | 0.4629 | 0.5371 | *p > a* | *p > a* |
| 11 | 1316 | 0.06479 | 40 | 1.0000 | 0.0000 | **p < 0.05** | **p < 0.05** |
| 12 | 1036 | 0.05100 | 24 | 1.0000 | 0.0000 | **p < 0.05** | **p < 0.05** |
| 13 | 321 | 0.01580 | 1 | 0.3688 | 0.6312 | *p > a* | *p > a* |
| 14 | 820 | 0.04037 | 0 | 0.0038 | 0.9962 | *p > a* | *p > a* |
| 15 | 616 | 0.03033 | 2 | 0.2202 | 0.7798 | *p > a* | *p > a* |
| 16 | 862 | 0.04244 | 1 | 0.0200 | 0.9800 | *p > a* | *p > a* |
| 17 | 1188 | 0.05848 | 3 | 0.0411 | 0.9589 | *p > a* | *p > a* |
| 18 | 269 | 0.01324 | 0 | 0.1653 | 0.8347 | *p > a* | *p > a* |
| 19 | 1474 | 0.07256 | 4 | 0.0289 | 0.9711 | *p > a* | *p > a* |
| 20 | 543 | 0.02673 | 1 | 0.1214 | 0.8786 | *p > a* | *p > a* |
| 21 | 232 | 0.01142 | 1 | 0.5429 | 0.4571 | *p > a* | *p > a* |
| 22 | 492 | 0.02422 | 6 | 0.9532 | 0.0468 | **p < 0.05** | *p > a* |
| X | 846 | 0.04165 | 0 | 0.0032 | 0.9968 | *p > a* | *p > a* |
| MRF | 5876 | 0.28927 | 79 | 0.9999 | 0.0000 | **p < 0.05** | **p < 0.05** |

e. Ancestral linkage group 2

The ancestral chordate linkage groups were reconstructed by Putnam et al. (2008) and later used by Strivastava et al. (2008). These paralogous groups of linked genes are present in the single copy in the chordate ancestor, retained in the single copy but rearranged in amphioxus, and duplicated twice by 2R WGD in the vertebrates. The majority of the genes linked to MRF loci in both human and amphioxus (n=79) are located in areas orthologous to ancestral linkage group 2.

| SC3 (Mb) | B.lan gene | Human ID | Chr | LG |
|---|---|---|---|---|
| 0.349 | ACP2 | NP_001601 | 11 | 2 |
| 0.369 | PSMA5 | NP_001186701 | 1 | 4 |
| 0.449 | ACACA | XP_011536567 | 12 | 12 |
| 0.563 | DUSP16 | NP_085143 | 12 | 6 |
| 0.936 | UTP20 | NP_055318 | 12 | 2 |
| 1.219 | PSMD13 | NP_002808 | 11 | 2 |
| 1.318 | ADAMTS15 | NP_620686 | 11 | 15 |
| 1.422 | KIAA0467 | XP_016856308 | 1 | 8 |
| 1.434 | PSMA1 | NP_683877 | 11 | 2 |
| 1.575 | FBXO3 | NP_208385 | 11 | 2 |
| 1.888 | IGF2 | NP_001121070 | 11 | 2 |
| 2.109 | ASCL1 | NP_004307 | 12 | 2 |
| 2.232 | CAPS2 | XP_011537180 | 12 | 2 |
| 2.247 | SNRPE | NP_001315566 | 1 | 4 |
| 2.270 | C11orf96 | NP_001138505 | 11 | 2 |
| 2.310 | ZFC3H1 | NP_659419 | 12 | 2 |
| 2.338 | IGF1 | XP_016874752 | 12 | 2 |
| 2.455 | C1orf69 | NP_001010867 | 1 | 15 |
| 2.960 | AKR1A1 | NP_006057 | 1 | 8 |
| 3.031 | NOTCH1 | XP_016875213 | 12 | 2 |
| 3.097 | AC009509.2 | NP_001139693 | 12 | 2 |
| 3.146 | MAPK8IP1 | NP_005447 | 11 | 2 |
| 3.158 | ATP5F1 | NP_001679 | 1 | |
| 3.166 | WDR77 | NP_077007 | 1 | |
| 3.358 | EFCAB4B | XP_011519339 | 12 | 2 |
| 3.483 | DKK3 | NP_001317149 | 11 | 2 |
| 3.523 | CMAS | NP_061156 | 12 | 2 |
| 3.647 | HAO1 | NP_057611 | 1 | 2 |
| 3.809 | STRAP | NP_009109 | 12 | 6 |
| 3.900 | MRPL23 | NP_066957 | 11 | 2 |
| 3.904 | NUP37 | NP_076962 | 12 | 2 |
| 3.929 | TBK1 | NP_037386 | 12 | 4 |
| 4.035 | PPP6R3 | NP_001339283 | 11 | 2 |
| 4.074 | FAM60A | NP_067061 | 12 | 2 |
| 4.136 | CAT | NP_001743 | 11 | 2 |
| 4.480 | HSD17B14 | NP_057330 | 19 | 15 |
| 4.496 | LOH12CR1 | NP_477517 | 12 | 6 |
| 4.499 | ALKBH3 | NP_631917 | 11 | 2 |
| 4.526 | C12orf5 | NP_065108 | 12 | 2 |
| 4.544 | EXT2 | XP_024304151 | 11 | 2 |
| 4.894 | OR6C68 | NP_001004740 | 11 | 2 |
| 4.904 | MTCH2 | NP_001304161 | 11 | 2 |
| 4.945 | PRMT3 | NP_005779 | 11 | 2 |
| 4.963 | CSDE1 | NP_001229820 | 1 | 2 |
| 4.990 | HORMAD1 | XP_011508356 | 1 | 11 |
| 5.041 | ARFGAP2 | XP_005253225 | 11 | 2 |
| 5.058 | TCP11L1 | NP_001139013 | 11 | 2 |
| 5.065 | ACTR6 | NP_071941 | 12 | 2 |
| 5.070 | C11orf10 | NP_055021 | 11 | 2 |
| 5.082 | DEPDC7 | NP_001070710 | 11 | 2 |
| 5.317 | ENSG00000262217 | NP_666018 | 12 | 2 |
| 5.508 | AMN1 | NP_001106873 | 12 | 2 |
| 5.539 | DHDH | NP_055290 | 19 | 15 |
| 5.751 | C12orf26 | NP_001306604 | 12 | 2 |
| 6.012 | Hypp2660 | NP_001303962 | 19 | 8 |
| 6.224 | MYOD1 | NP_002469 | 11 | 2 |
| 6.323 | C14orf149 | NP_079405 | 19 | 15 |
| 6.339 | PATL1 | NP_689929 | 11 | 2 |
| 6.418 | TSG101 | NP_006283 | 11 | 2 |
| 6.671 | SYT12 | NP_001305702 | 11 | 12 |
| 6.709 | CREB3L1 | XP_006718443 | 11 | 2 |
| 6.913 | KCNQ1 | NP_861463 | 11 | 2 |
| 7.015 | CDKN1B | NP_004055 | 12 | 6 |
| 7.262 | ENSG00000265133 | NP_066403 | 1 | 11 |
| 7.374 | INSC | NP_001036001 | 11 | 2 |
| 7.572 | Hypp2695 | NP_002518 | 12 | 2 |
| 7.628 | BBOX1 | NP_003977 | 11 | 2 |
| 7.698 | ANO4 | XP_011536217 | 12 | 2 |
| 7.934 | WEE1 | NP_003381 | 11 | 2 |
| 7.948 | ODF3 | NP_444510 | 11 | 2 |
| 7.970 | MS4A4A | NP_068769 | 11 | 2 |
| 8.038 | NELL2 | NP_001275642 | 11 | 2 |
| 8.201 | TMEM216 | NP_001167461 | 11 | 2 |
| 8.281 | LRRC4C | NP_065980 | 11 | 2 |
| 8.377 | COPB1 | NP_057535 | 11 | 2 |
| 8.389 | INCENP | NP_064623 | 11 | 15 |
| 8.402 | ENSG00000262489 | NP_036382 | 11 | 2 |
| 8.479 | NADSYN1 | NP_060631 | 11 | 17 |
| 8.512 | USP47 | XP_011518498 | 11 | 2 |

Locations of amphioxus genes on scaffold 3 (SC3 (Mb): location in megabases), their annotated gene names, the orthologous human protein ID, its chromosomal location in the human genome (Chr) and the corresponding ancestral linkage group (LG).

f. Ancestral linkage groups on chromosomes 1, 11, 12, and 19

These are the regions of each of the four human MRF locus-linked chromosomes assigned to various linkage groups. The start and end positions are in bases, from adaptation of supplementary table “Table S8.2: Human chromosome segment boundaries and their groupings into ancestral chordate linkage groups” from Srivastava et al. (2008) to Putnam et al. (2008) naming for *B. floridae* (Oxford grid in supplementary information; supplementary Note 8).

| Chromosome 1 | | |
|---|---|---|
| Start | End | LG |
| 1 | 23526132 | 6 |
| 23526133 | 43180611 | 17 |
| 43180613 | 85630086 | 8 |
| 85630087 | 91246779 | 10 |
| 91246780 | 102063750 | 8 |
| 102063751 | 110948178 | 4 |
| *110948179* | *112039666* | - |
| 112039667 | 114548603 | 4 |
| 114548604 | 120491741 | 2 |
| 120491742 | 155279431 | 11 |
| 155279432 | 159410831 | 15 |
| 159410832 | 199491263 | 10 |
| 199491264 | 210027080 | 4 |
| 210027081 | 248197708 | 15 |

| Chromosome 19 | | |
|---|---|---|
| Start | End | LG |
| 1 | 9866833 | 11 |
| 9866834 | 15445888 | 8 |
| 15445889 | 19486034 | 11 |
| 19486035 | 40572552 | 9 |
| 40572553 | 53188588 | 15 |
| 53188589 | 64790642 | 2 |

| Chromosome 11 | | |
|---|---|---|
| Start | End | LG |
| 1 | 3646292 | 2 |
| 3646293 | 8513570 | 7 |
| 8513571 | 61969973 | 2 |
| 61969974 | 66588478 | 15 |
| 66588479 | 67708722 | 12 |
| 67708723 | 70921654 | 2 |
| 70921655 | 111081603 | 17 |
| 111081602 | 135361180 | 15 |

| Chromosome 12 | | |
|---|---|---|
| Start | End | LG |
| 1 | 5733870 | 2 |
| 5733871 | 16622256 | 6 |
| 16622257 | 32686135 | 2 |
| 32686136 | 46754658 | 4 |
| 46754659 | 56444814 | 16 |
| 56444815 | 67572059 | 4 |
| 67572060 | 90031496 | 2 |
| 90031497 | 99176013 | 4 |
| 99176012 | 107108041 | 2 |
| 107108042 | 133278857 | 12 |

## References

Putnam NH, Butts T, Ferrier DEK, Furlong RF, Hellsten U, Kawashima T, Robinson-Rechavi M, Shoguchi E, Terry A, Yu J-K, et al. 2008. The amphioxus genome and the evolution of the chordate karyotype. Nature. 453(7198):1064–1071. doi:10.1038/nature06967.

Srivastava M, Begovic E, Chapman J, Putnam NH, Hellsten U, Kawashima T, Kuo A, Mitros T, Salamov A, Carpenter ML, et al. 2008. The Trichoplax genome and the nature of placozoans. Nature. 454(7207):955–960. doi:10.1038/nature07191.

7. WMISH of amphioxus *MRFs* in *B. floridae* and *B. lanceolatum* embryos
    a. Gastrulae

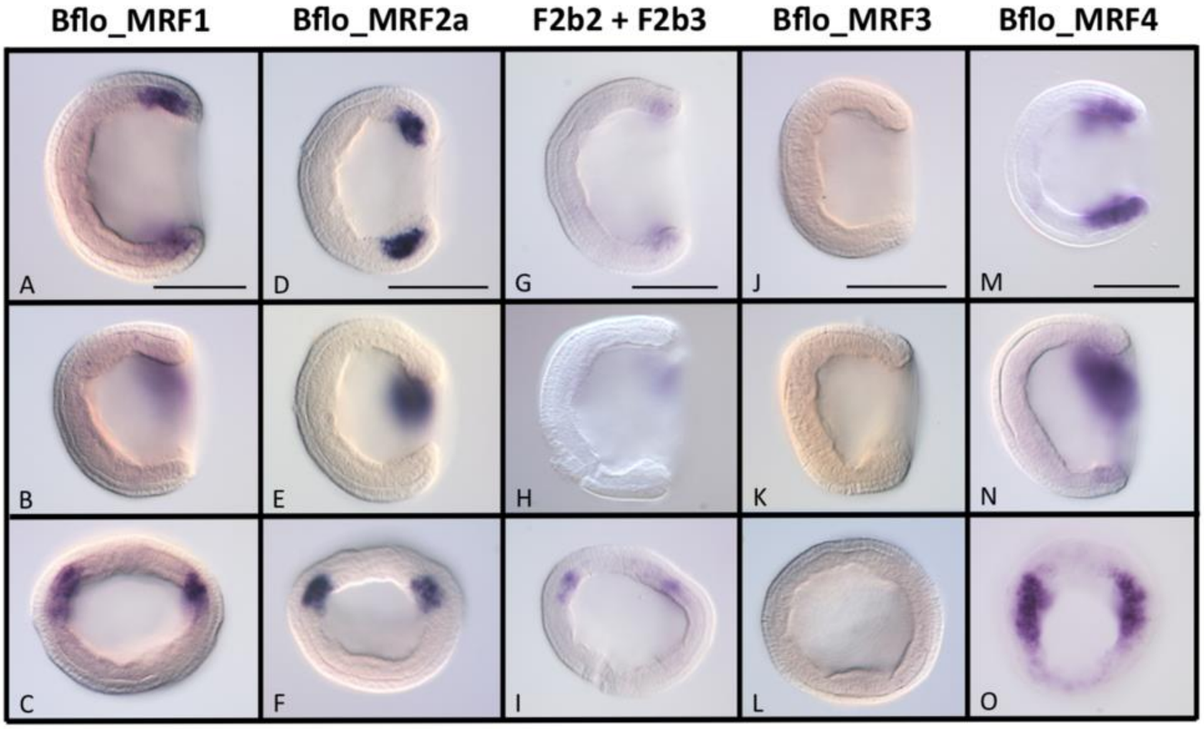


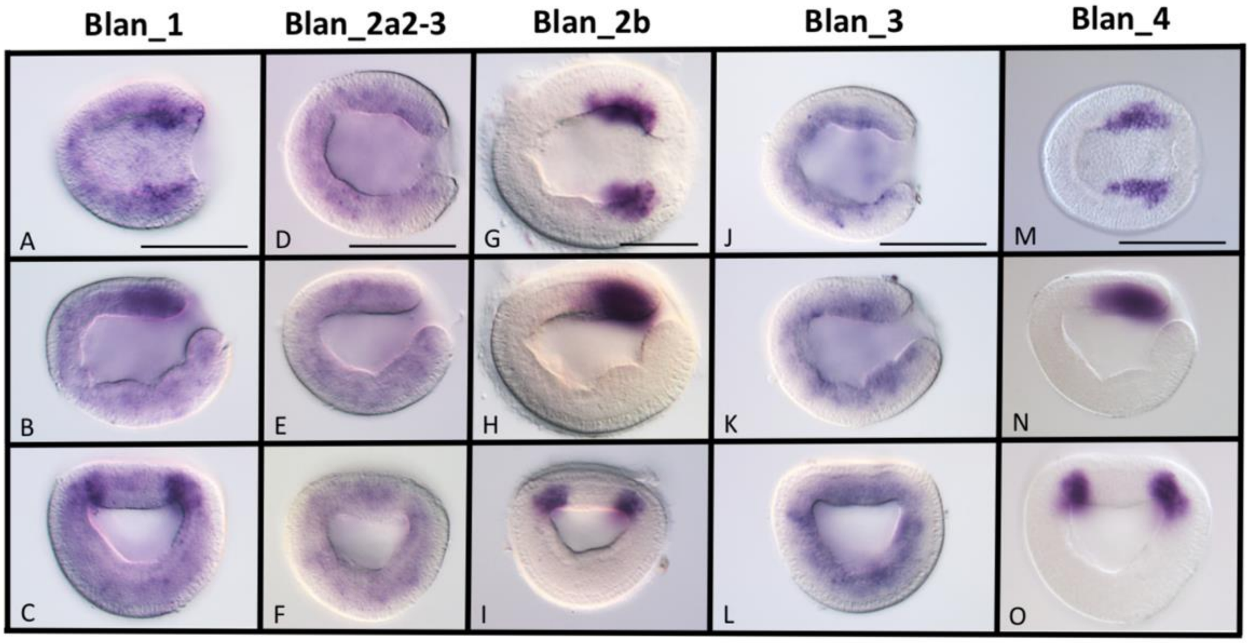

b. Early-mid neurulae

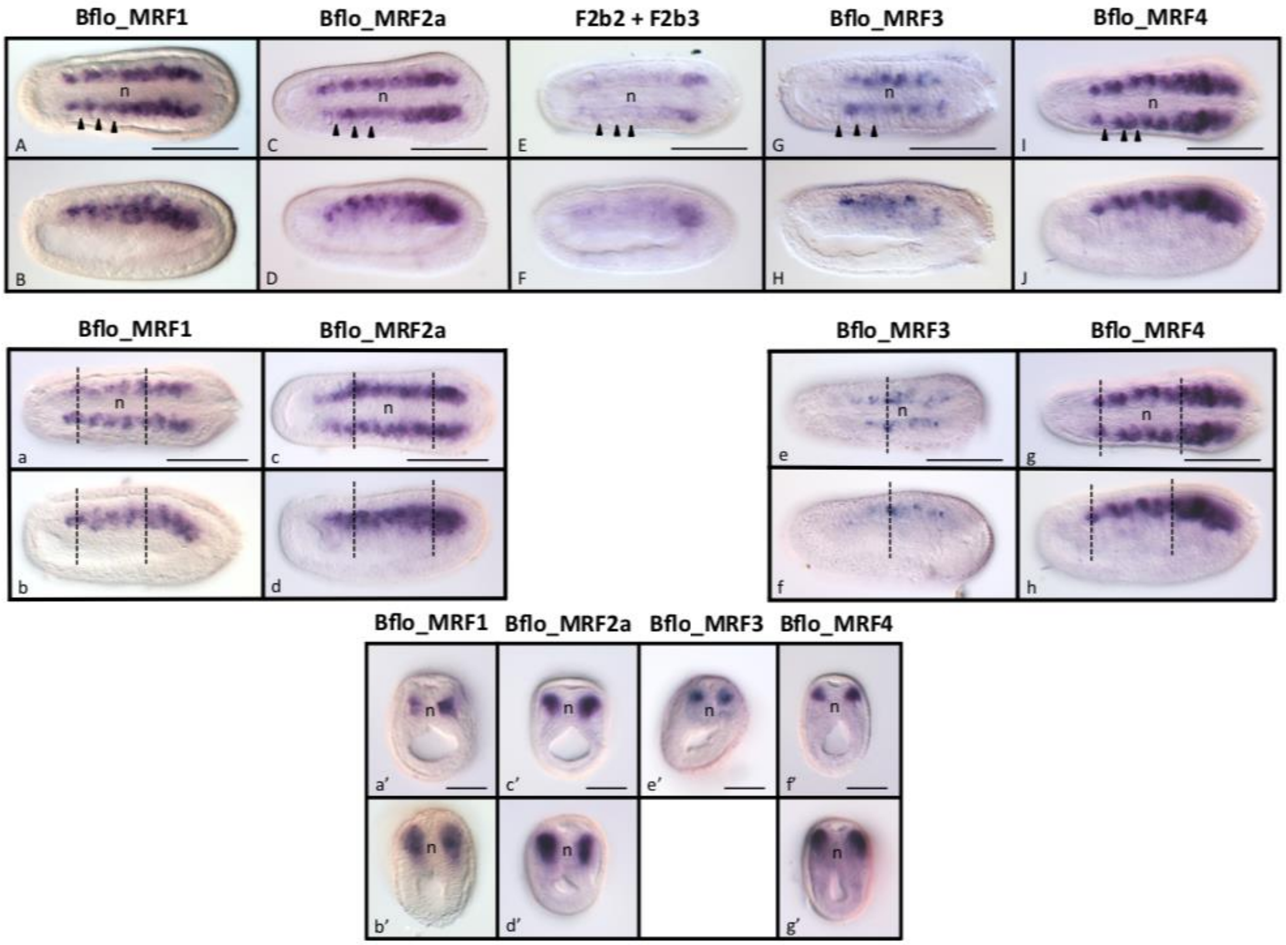


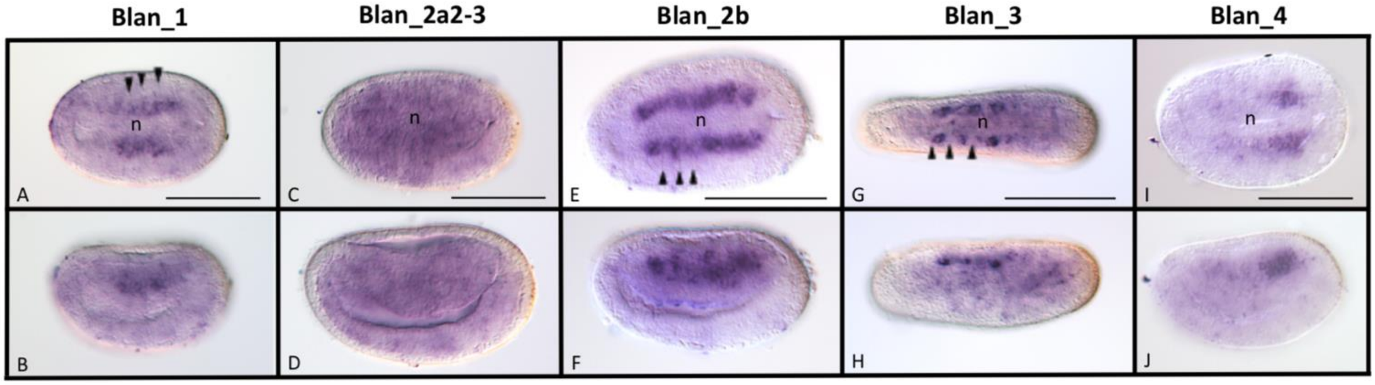

c. Late neurulae

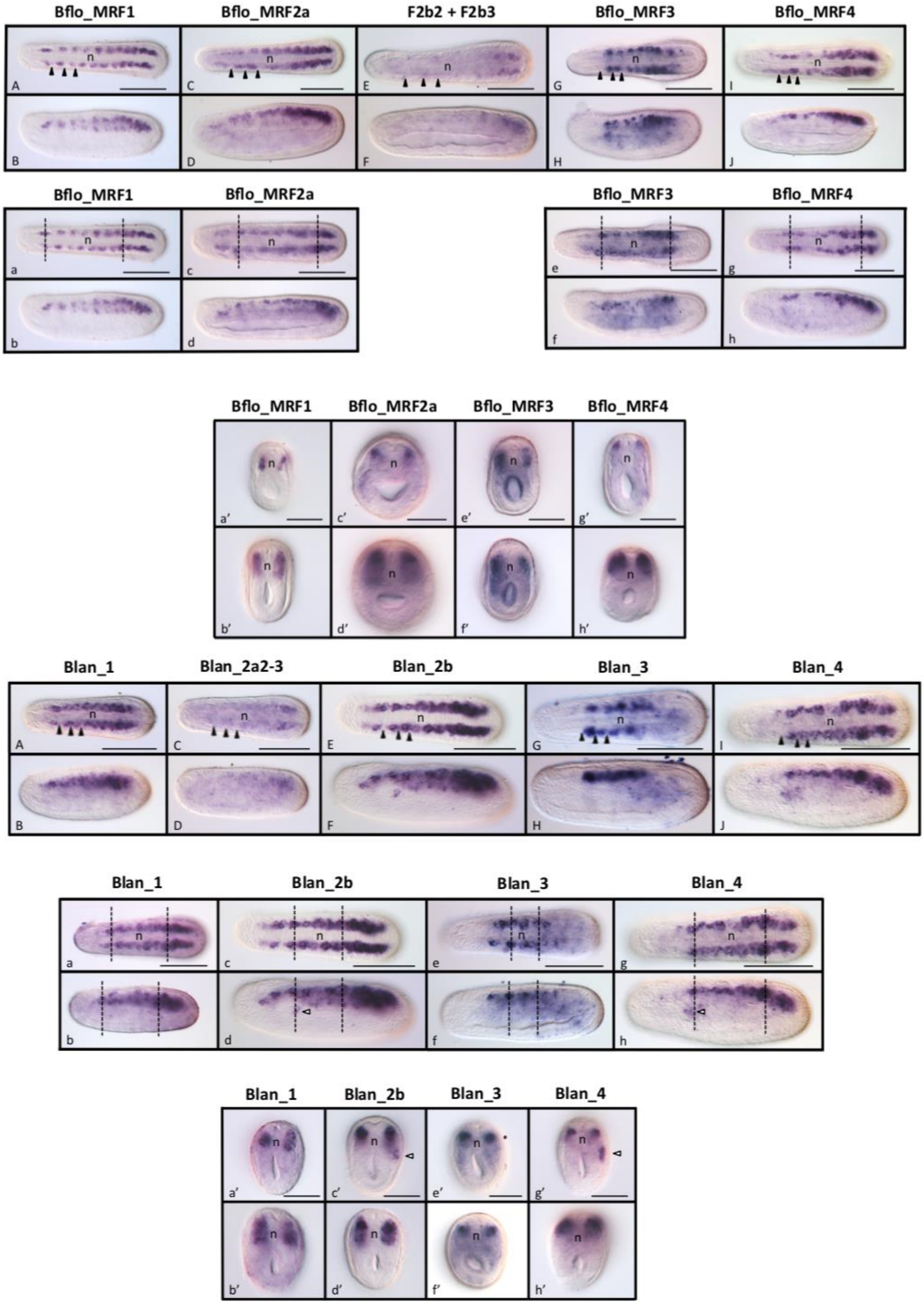

d. Early larvae

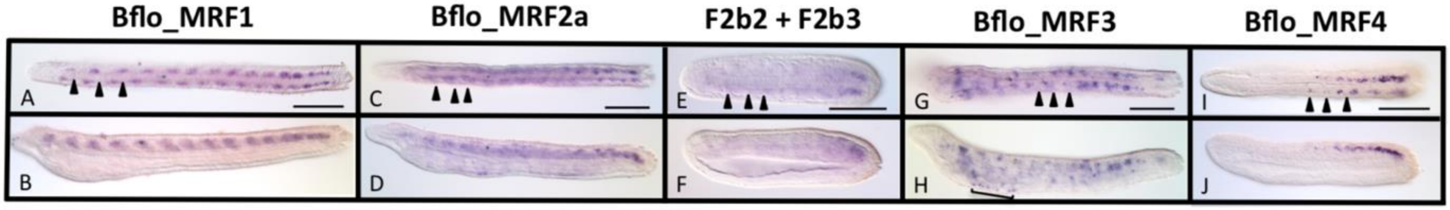


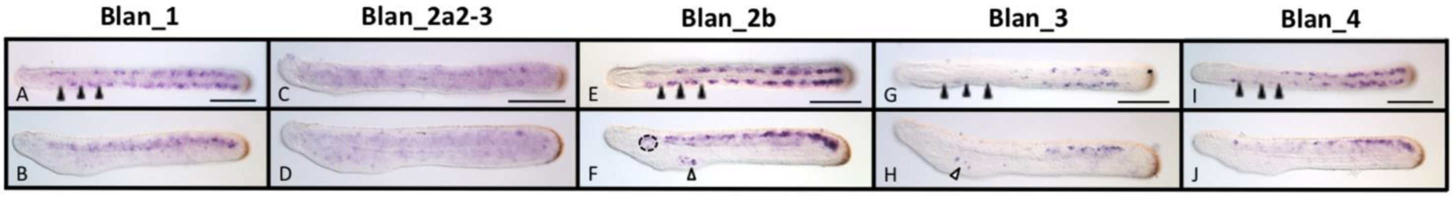


e. Late larvae

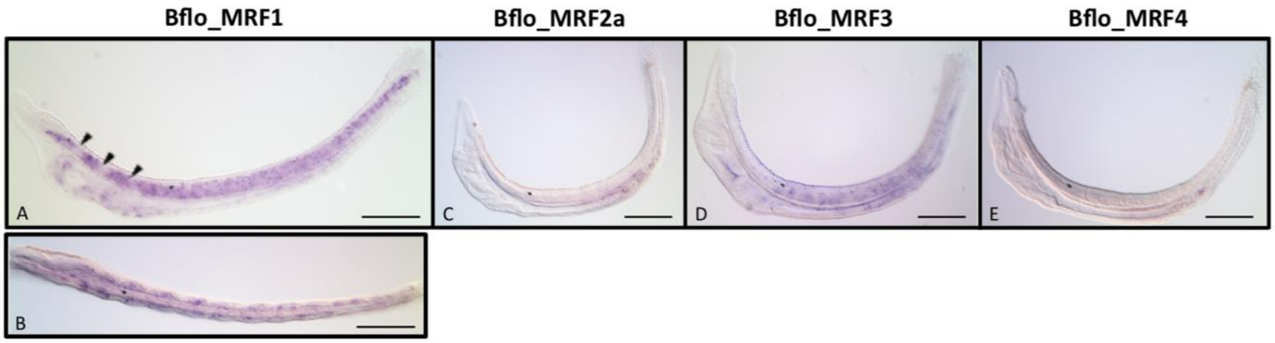


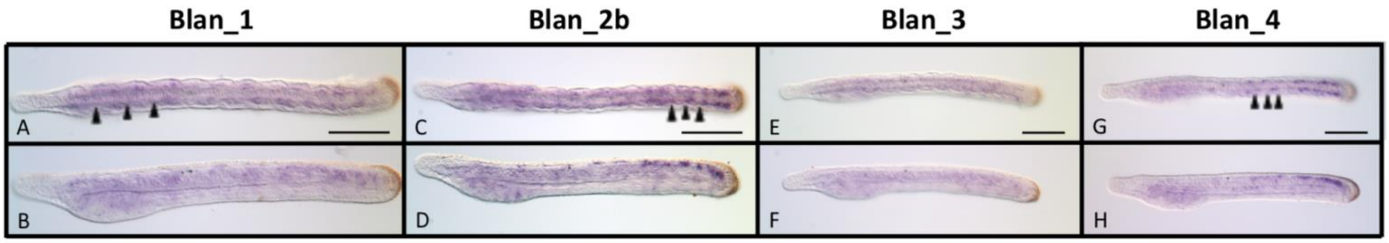

f. Probes

| *B. floridae* | Primer sequence (5'-3') | DNA template | Ta ˚C | PCR product |
|---|---|---|---|---|
| Bflo_MRF1 | F: GTAGGGGCGACTTTGAAGACAC<br>R: TCATCTTGGCGGGTTGCT | Total adult cDNA | 54.6 | 684bp, complete cds (715bp, including partial 5'UTR) |
| Bflo_MRF2a | F: ATGATGAACTACACAGAGCTGAGCA<br>R: TTATGATCTTAGTGGGGTGTAGCC | Total adult cDNA | 55.1 | 690bp, complete cds |
| F2a2 | F: ATCGGTAACTGATTGTTTCACTGC<br>R: ATGACGCCATGTTGTCTTCC | Genomic DNA | 58 | Bflo_MRF2a_exon2: 201bp, partial sequence |
| F2a3 | F: TGTTGTTTTGATGATATTTATAAGTTGTTG<br>R: CCCTTATGATCTTAGTGGGGTGTA | Genomic DNA | 58 | Bflo_MRF2a_exon3: 203bp, total sequence |
| F2b2 | F: GCATCACTAAATGGTGACAAGAACT<br>R: CAATTCAATTCCGACAGAAAGATAA | Genomic DNA | 58 | Bflo_MRF2b_exon2: 213bp, total sequence |
| F2b3 | F: TAGTAGGAGGCTAGGAGGAGTAGGT<br>R: GCCATCTTGTTTCCATGACTACAT | Genomic DNA | 58 | Bflo_MRF2b_exon3: 194bp, total sequence |
| Bflo_MRF3 | F: ATGGCGGTGGTTGAGAGGAG<br>R: TCACTGCGGTGTGCTTCTTG | Total adult cDNA | 66.1 | 771bp, complete cds |
| Bflo_MRF4 | F: CGGCTGCTACAGTTACACCA<br>R: GAATGACGTAATGTTGTCCACA | Total adult cDNA | 55 | 646bp, partial cds |
| *B. lanceolatum* | Primer sequence (5'-3') | DNA template | Ta ˚C | PCR product |
| Blan_MRF1 | F: GTAGGGGCGACTTTGAAGACAC<br>R: TCATCTTGGCGGGTTGCT | Adult muscle cDNA | 54 | 684bp, complete cds (715bp including partial 5'UTR) |
| L2a2-3 | F: CTGCGAACAGTCCCGACAGT<br>R: ATCTTAGTGGGGTGTAGCCTTGGT | Adult muscle cDNA | 58 | Blan_MRF2a_exon2-3: 198bp, partial sequence |
| Blan_MRF2b | F: ATGATGAACTACACAGAGCTGAGCA<br>R: GTTGACGATGGACGACAGG | Adult muscle cDNA | 54 | Blan_MRF2b: 642bp, partial cds |
| L2b2-3 | F: CTCGTCATCAGAGTCAAGTTTCAA<br>R: GTTGACGATGGACGACAGGTTAT | Adult muscle cDNA | 58 | Blan_MRF2b_exon2-3: 136bp, partial sequence |
| Blan_MRF3 | F: ATGGCGGTGGTTGAGAGGAG<br>R: TCACTGCGGTGTGCTTCTTG | Adult muscle cDNA | 54 | 774bp, complete cds |
| Blan_MRF4 | F: CGGCTGCTACAGTTACACCA<br>R: GAATGACGTAATGTTGTCCACA | Adult gonadal cDNA | 54 | 631bp, partial cds |

Note:

Since *MRF2a* and *MRF2b* have identical first exon sequences, the whole coding sequence probes for *B. floridae MRF2b* and *B. lanceolatum MRF2a* would cross-hybridise with *B. floridae MRF2a* and *B. lanceolatum MRF2b* respectively. Therefore, the expression pattern may not represent the specific single-gene pattern of both of the *MRF2-* genes due to sequence similarity. Probes that were made against only the sequence of the second and third exons gave weaker signals with poorer signal:noise ratios, which risks underrepresenting the full expression domain

of these genes. Still, besides the issue with *MRF2a* and *MRF2b*, we see overlapping but distinct expression patterns for each of the other three genes.

g. Extended description

All of the MRFs except for MRF3 are first detected in the late gastrula stage in the dorsolateral mesodermal precursor cells on either side of the dorsal lip of the blastopore in *B. floridae*. MRF4 was more broadly expressed throughout the paraxial mesoderm than the other MRFs, and MRF2b was only weakly expressed, though the four MRFs expressed at this stage were generally overlapping. In *B. lanceolatum*, the pattern was the same, except neither MRF3 nor MRF2a were detected at this stage.

In the early-mid neurula, MRFs were expressed in the somites, though *B. lanceolatum* MRF2a showed no distinct expression pattern. MRF1 was expressed in the medioventral section of the somites while MRF2b was expressed in the medial area of the anterior somites, as well as the entire posterior somites. MRF3 was first detected at this stage and was expressed in the dorsal somitic area of just the central somites. MRF4 was restricted to the newly formed somites. Like in *B. lanceolatum*, in *B. floridae*, MRF1 was expressed in the medioventral half of all somites, while and MRF3 was only expressed in the central somites. *B. floridae* MRF2a, however, was strongly detected in the posterior-most somite pair, similar to MRF4, though MRF2a was dorsomedially expressed while MRF4 was dorsolateral, so that these two genes seemed to mirror each other. MRF2b was only weakly detected in the newly formed somites.

In the late neurula, in both species, MRF1 was expressed in all mature somites and throughout the myotome, with a stronger signal from posterior somites. MRF3 was detected only in central somites, but in *B. floridae*, expression was shifted ventrolaterally while it remained in the dorsal region in *B. lanceolatum*. *B. floridae* MRF2a and *B. lanceolatum* MRF2b were expressed in the dorsomedial domain of anterior somites and had a wider expression pattern throughout the posterior somites. *B. floridae* MRF2b and *B. lanceolatum* MRF2a were only weakly detected and only in the newly formed somites. MRF4 was expressed dorsolaterally in all somites in *B. lanceolatum*, with a wider expression pattern in the posterior somites, and was also detected as non-myotomal expression in a somatic layer of mesoderm under the second and third somite of the left body side. In *B. floridae*, MRF4 was expressed in the dorsal region of the anterior and posterior, but not in central somites, with a more intense and dorsolateral expression in the posterior somites.

In the early larval stage, MRF1 was still expressed in all somites and expression was stronger in the posterior somites in *B. floridae*, though the signal was reduced and not homogeneous in *B. lanceolatum*. The pattern of expression of *B. lanceolatum* MRF2a and *B. floridae* MRF2b persisted, and these were only expressed weakly in the newly formed somites. *B. lanceolatum* MRF2b and *B. floridae* MRF2a were both still weakly expressed in anterior and central somites,

and stronger in more posterior somites. *B. lanceolatum* MRF2b also was detected in the first gill slit and possibly in the pre-oral pit. In both species, MRF3 was absent from the anterior-most and newly formed somites, though the signal was more reduced and dorsolateral in *B. lanceolatum,* while in *B. floridae,* it was expressed more dorsally to the anterior and more ventrally to the posterior. In some *B. floridae* embryos, MRF3 was detected in the left-side gill slits, while in *B. lanceolatum,* expression was also detected in the mouth rudiment. In both species, MRF4 expression was mainly dorsolateral and restricted to posterior somites.

By the late larval stage, only MRF1 was detected in *B. floridae*, mostly localised in the central part of the muscle fibres within the myomeres. In *B. lanceolatum*, MRF1 was weakly detected in all somites, while MRF2b was only detected in the dorsal area of the posterior somites. MRF4 expression was further reduced dorsomedially to just the posterior and newly formed somites, while MRF2a and MRF3 were not detected at this stage.
